# Development of Inhibitors, Probes, and PROTAC Provides a Complete Toolbox to Study PARK7 in the Living Cell

**DOI:** 10.1021/acs.jmedchem.3c02410

**Published:** 2024-05-07

**Authors:** Yuqing Jia, Merve Oyken, Robbert Q. Kim, Rayman T.N. Tjokrodirijo, Arnoud H. de Ru, Antonius P. A. Janssen, Stephan M. Hacker, Peter A. van Veelen, Paul P. Geurink, Aysegul Sapmaz

**Affiliations:** † Department of Cell and Chemical Biology, Division of Chemical Biology and Drug Discovery, Leiden University Medical Center, Einthovenweg 20, Leiden 2333 ZC, The Netherlands; ‡ Laboratory for Organic Chemistry, Department of Chemistry and Applied Biosciences, ETH Zürich, Vladimir-Prelog-Weg 3, Zürich CH-8093, Switzerland; § Center for Proteomics and Metabolomics, Leiden University Medical Center, Albinusdreef 2, Leiden 2333 ZA, The Netherlands; ∥ Department of Molecular Physiology, Leiden Institute of Chemistry, Leiden University, Einsteinweg 55, Leiden 2333 CC, The Netherlands

## Abstract

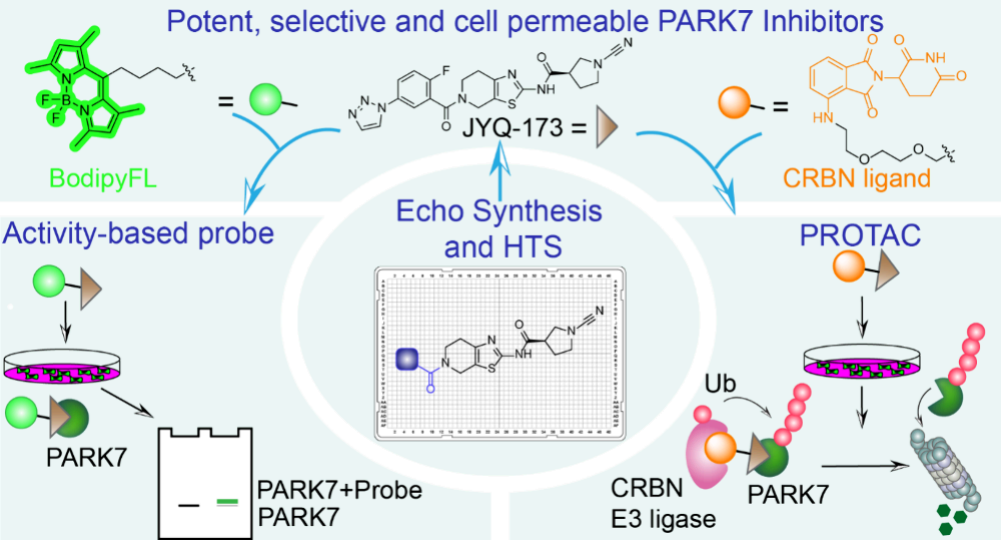

The integration of diverse chemical tools like small-molecule inhibitors, activity-based probes (ABPs), and proteolysis targeting chimeras (PROTACs) advances clinical drug discovery and facilitates the exploration of various biological facets of targeted proteins. Here, we report the development of such a chemical toolbox for the human Parkinson disease protein 7 (PARK7/DJ-1) implicated in Parkinson’s disease and cancers. By combining structure-guided design, miniaturized library synthesis, and high-throughput screening, we identified two potent compounds, **JYQ-164** and **JYQ-173**, inhibiting PARK7 *invitro* and in cells by covalently and selectively targeting its critical residue, Cys106. Leveraging **JYQ-173**, we further developed a cell-permeable Bodipy probe, **JYQ-196**, for covalent labeling of PARK7 in living cells and a first-in-class PARK7 degrader **JYQ-194** that selectively induces its proteasomal degradation in human cells. Our study provides a valuable toolbox to enhance the understanding of PARK7 biology in cellular contexts and opens new opportunities for therapeutic interventions.

## Introduction

Human Parkinson disease protein 7 (PARK7), also known as DJ-1, is a small (∼20 kDa) multifunctional protein,^1,2^ which is associated with various types of cancer and Parkinson’s disease.^3−5^ Throughout the years, PARK7 has been found to play a major role in protecting cells from stress conditions, especially oxidative stress, via its enzymatic and nonenzymatic functions.^6^ The key element for PARK7 functioning is the highly conserved cysteine residue at position 106. Cys106 serves as the active-site residue for the enzymatic glyoxalase activity of PARK7, while oxidation of Cys106 is essential for PARK7 to accomplish nonenzymatic functions, including antioxidant, chaperone, cotranscription factor, and antiapoptotic/ferroptotic functions.^7^ On the other hand, the excessive oxidation of the Cys106 residue leads to the loss of its neuroprotective activity and the development of neurodegenerative diseases.^8^

Its critical roles in a plethora of biological processes, including cell protective/survival activities and promoting tumorigenesis, demonstrate PARK7 to be an attractive therapeutic target. However, exactly how PARK7 fulfills its multifarious functions remains to be explored. This highlights the need for potent inhibitors and degraders that specifically target PARK7 in cells to explore PARK7 biology and advance drug discovery. A few small-molecule inhibitors were initially identified to bind PARK7,^9,10^ and this was later extended by a group of inhibitors based on the endogenous metabolite isatin, which bound PARK7 and reacted with Cys106 in a covalent manner.^11−13^ Amino-epoxycyclohexanones were also reported to covalently modify Cys106, and the inclusion of an alkyne moiety in these compounds allowed for *in situ* profiling of PARK7 by two-step labeling.^14^ We recently reported the development of cyanimide-containing inhibitor **JYQ-88**,^15^ along with two fluorescent probe variants, which covalently react with PARK7 Cys106, and demonstrated the successful application of these compounds in cell lysate. However, only a few compounds developed so far have been shown to engage with PARK7 in the context of live cells,^11,12,14^ which encouraged us to develop highly potent, cell-permeable small-molecule compounds specifically targeting the Cys106 residue of PARK7.

Taking **JYQ-88** as a starting point, we here applied combined strategies of structure-guided design, miniaturized synthesis, and high-throughput screening to obtain improved PARK7 inhibitors **JYQ-164** and **JYQ-173** having submicromolar potency in cells. Both compounds specifically bind PARK7 and react to the Cys106 residue in cells as evidenced from a streamlined cysteine activity-based protein profiling (SLC-ABPP) experiment,^16^ with **JYQ-173** being the most potent one. Moreover, we report a cell-permeable fluorescent probe **JYQ-196** with a Bodipy dye, which was shown to covalently label PARK7 activity in both HEK293T and A549 cells. Finally, we report a first-in-class PARK7 degrader **JYQ-194**, which induced PARK7 degradation in different tumor cell lines. Altogether, we have developed a complete chemical toolbox of PARK7 to boost further studies on its diverse functions and future drug development.

## Results and Discussion

### Combining Structure-Guided Design and High-Throughput Synthesis to Discover Improved PARK7 Inhibitors

**Figure 1:**
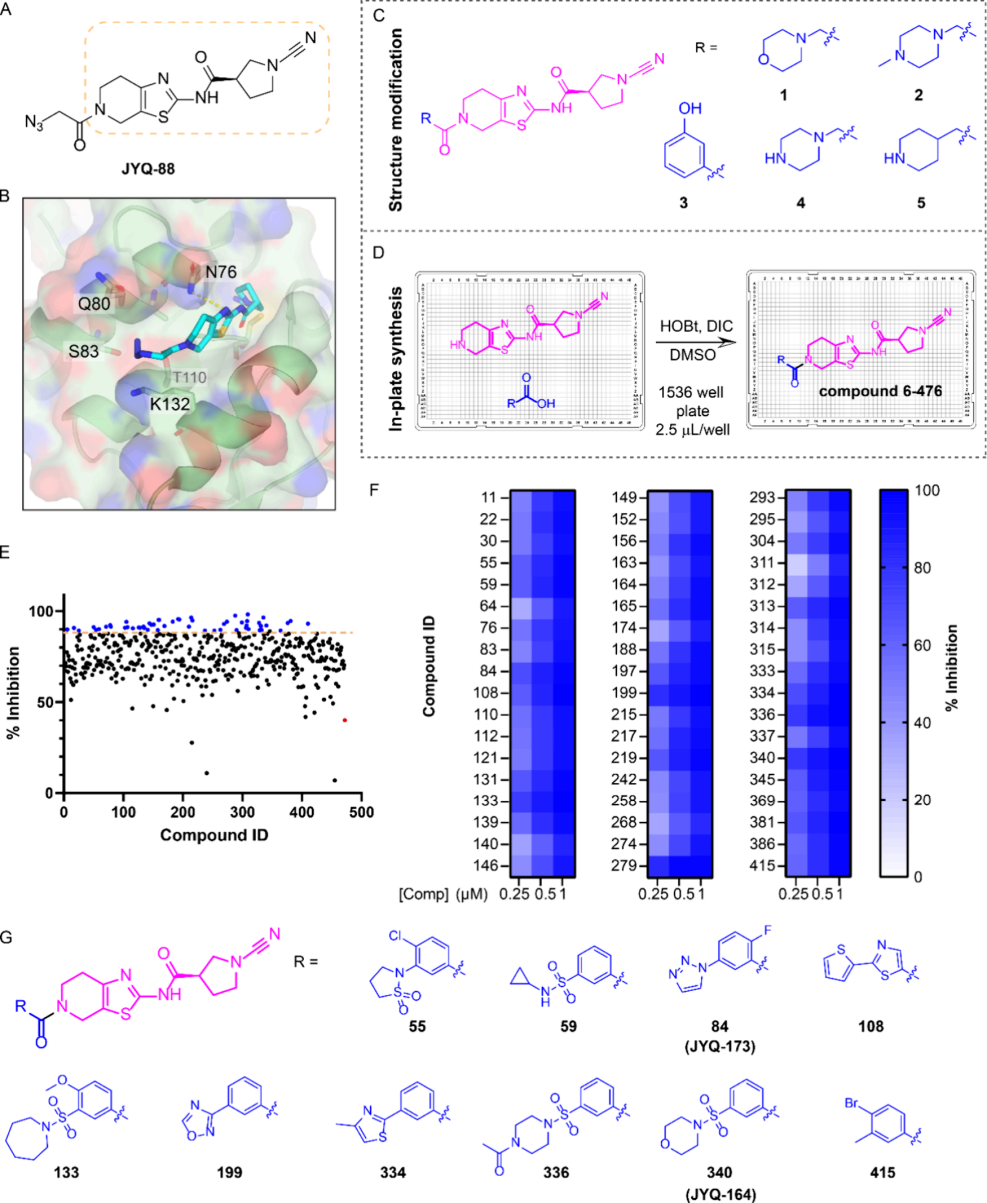
Discovery of improved PARK7 inhibitors. (A) Structure of PARK7 inhibitor **JYQ-88**. (B) Crystal structure of PARK7-**JYQ-88** complex (PDB 7PA3) showing an unoccupied pocket around the azidoacetyl moiety. (C) Chemical structures of designed compounds with a hydrophilic substituent. (D) Schematic illustration of in-plate synthesis to build a compound library. (E) Screening results using an FP assay at 1 μM compound concentration. Blue color represents compounds showing over 90% inhibition. Red color shows inhibition data for the amine precursor compound. (F) Heatmap displaying validation of the screening hits at 0.25, 0.5, and 1 μM using the FP assay. (G) Chemical structures of the resynthesized top 10 compounds.

**Scheme 1:**
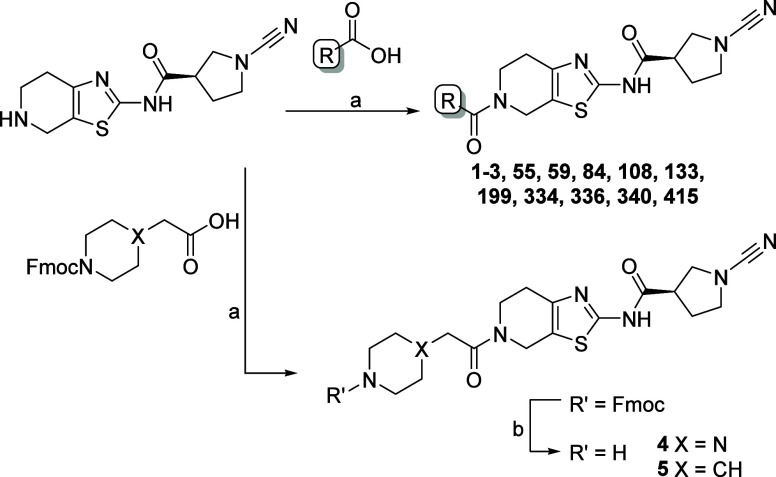
Synthesis of PARK7 Inhibitors Reagents and conditions: (a) carboxylic acid, HCTU, DIPEA, DCM; (b) DBU, DCM.

The PARK7 inhibitor **JYQ-88** (Figure 1A) that we recently reported potently inhibits PARK7 in cell lysates.^15^ However, administering the compound to intact HEK293T cells revealed that it showed poor cellular engagement (Supporting Information Figure S1). Therefore, taking **JYQ-88** as a starting point, we conducted an optimization to further improve the molecule in terms of inhibitory potency, specificity, and cellular uptake. Our previously generated crystal structure of the PARK7-**JYQ-88** complex (PDB 7PA3) showed that the azidoacetyl moiety of **JYQ-88** does not interact with PARK7, thereby leaving the mainly hydrophilic pocket surrounding this moiety largely unoccupied (Figure 1B). We therefore opted to improve the inhibitor by introducing different substituents replacing the azidomethyl moiety using two strategies. First, a small tailored library was designed and synthesized to investigate whether the introduction of larger, hydrophilic substituents on **JYQ-88** would enhance the interaction with the PARK7 pocket. We introduced five different hydrophilic moieties, including morpholine, 4-methyl-piperazine, 3-hydroxyphenyl, piperazine, and piperidine replacing the azidomethyl moiety (compounds **1**–**5** in Figure 1C, Scheme 1). The second approach involved the generation of a compound library containing diverse substituents at the azidomethyl position. The crude compound library was prepared using Echo acoustic dispensing and in-plate synthesis, where we performed an amidation reaction in DMSO with the cyanimide amine moiety of **JYQ-88** (Figure 1A, yellow box) and 471 diverse carboxylic acids using *N*,*N*′-diisopropylcarbodiimide (DIC) and hydroxybenzotriazole (HOBt) in a 1536-well plate (compound **6-476** in Figure 1D, Supplementary Data S1). We next performed a high-throughput screen of this crude compound library using our in-house developed PARK7 fluorescence polarization (FP) assay.^15^ The compounds, at 1 μM final concentration, were incubated with recombinant PARK7, followed by incubation with carboxyrhodamine-tagged FP reagent **JYQ-107**, which labels all residual active PARK7, and monitoring of the FP signal. Overall, most of the compounds showed over 50% inhibition, and we specifically selected the 54 hits showing over 90% inhibition (Figure 1E). All of these hits could be validated at 1, 0.5, and 0.25 μM final concentrations in the same FP assay (Figure 1F, Supplementary Data S1). As all compounds exist as crude mixtures in the library plate, we selected the top 10 hits, which were resynthesized and purified for further biochemical characterization (Figure 1G, Scheme 1, Supplementary Data S1). Of note here is that the cyanimide amine precursor for all compounds (Figure 1D, left) also inhibits PARK7. An inhibition of ∼40% at 1 μM of crude mixture was found in the HTS (Figure 1E), but inhibition potency of the pure compound was strongly reduced to 3 and 20% at 0.25 and 0.5 μM, respectively (Supporting Information Figure S2).

### **JYQ-164** and **JYQ-173** Potently Bind with PARK7 in Cells

**Figure 2:**
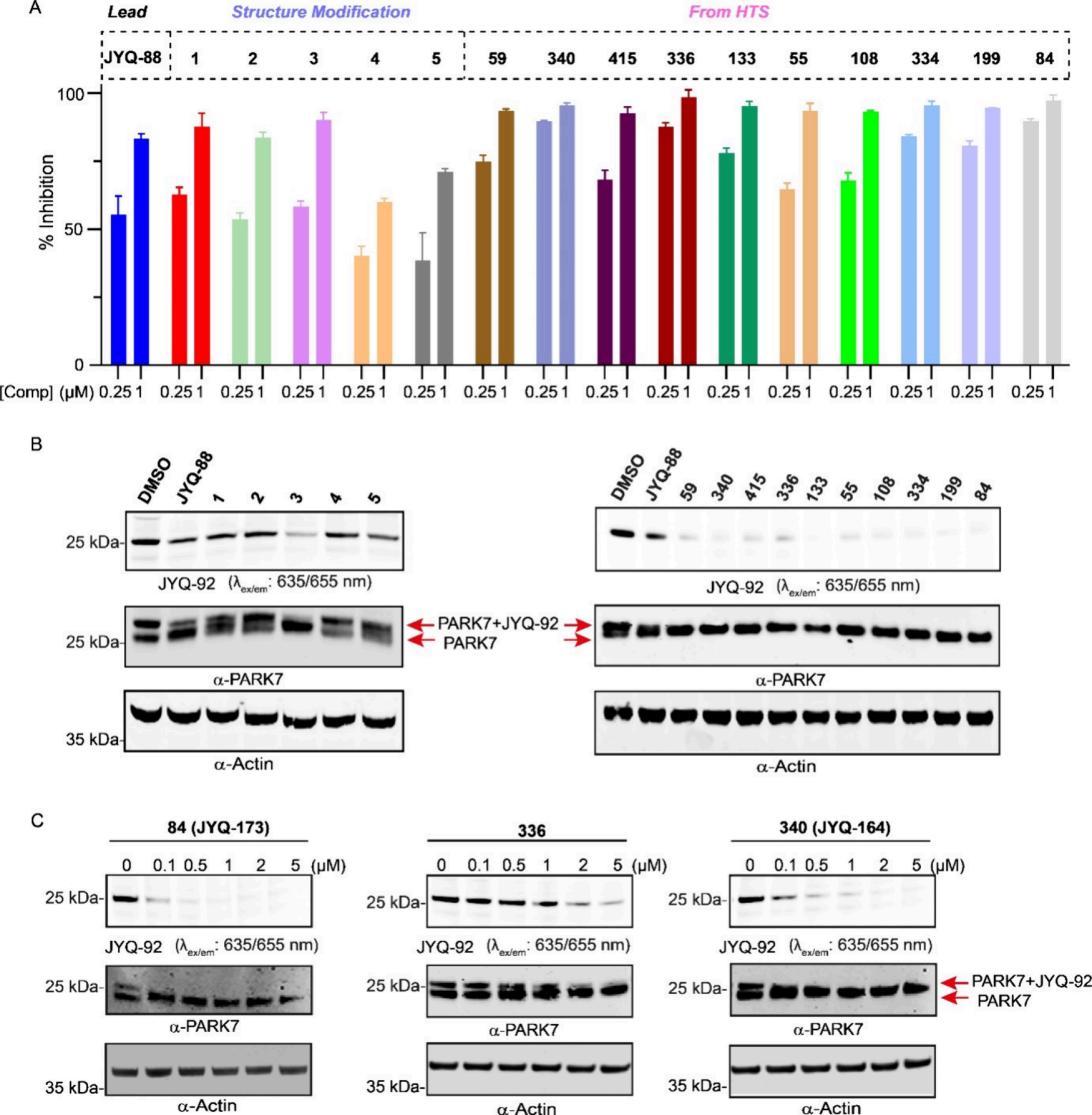
Assessment of potency and cell permeability of the selected compounds. (A) Inhibition of PARK7 at 0.25 and 1 μM final concentration of 5 compounds designed from structural modification and top 10 compounds from HTS, determined using the PARK7 FP assay.^15^ (B) Fluorescent probe labeling of PARK7 remaining activity after inhibitor treatment to investigate cellular engagement. HEK293T cells were treated with the indicated compounds for 24 h. After cell lysis and incubation with the fluorescent PARK7 probe **JYQ-92** for 1 h, the samples were analyzed by SDS-PAGE, fluorescence scanning, and immunoblot against PARK7 and β-actin. β-Actin was used as a loading control. (C) Target engagement of compounds **84**, **336**, and **340** in HEK293T cells in a dose–response manner. HEK293T cells were incubated with the compounds at the indicated final concentrations for 24 h, prior to cell lysis and incubation with PARK7 probe **JYQ-92**. The samples were analyzed by SDS-PAGE, fluorescence scanning, and immunoblot against PARK7 and β-actin. β-Actin was used as a loading control.

The 15 pure compounds from the two different approaches (5 compounds from structure modification and 10 compounds from HTS) were assessed for their potency to inhibit PARK7 at 0.25 and 1 μM final concentrations using the FP assay (Figure 2A). Overall, 11 compounds showed improved potency compared to **JYQ-88**, all of which contain an aromatic substituent. Among these, compounds **84**, **336**, and **340**, which were discovered via HTS, displayed the highest potency with close to 100% inhibition at 0.25 μM, while compound **3**, the only compound from the designed structural modification, showed a slight improvement in potency compared to **JYQ-88**. By contrast, the compounds with polar groups, such as 4-methyl-piperazine, piperazine, and piperidine, showed decreased potency compared to **JYQ-88**.

Since **JYQ-88** is a potent inhibitor *in vitro* but did not show inhibition of PARK7 in cells (Supporting Information Figure S1), we next evaluated the cellular target engagement of all 15 compounds in a cell-based competition assay. Live HEK293T cells were treated with 5 μM of compounds for 24 h, followed by cell lysis and incubation with our previously developed SulfoCy5 PARK7 probe **JYQ-92**.^15^ Samples were resolved by SDS-PAGE and analyzed by fluorescence scanning and Western blotting (Figure 2B). The target engagement of cellular PARK7 is reflected by the disappearance of the PARK7 labeling band in the fluorescence gel scan and by the loss of the shifted probe-labeled PARK7 band in the Western blot. This revealed that all of the 11 aforementioned compounds with improved potency were able to bind to PARK7 in cells, indicating improved cellular target engagement. Remarkably, the remaining 4 compounds (**1**, **2**, **4**, **5**) with decreased potency did not bind with PARK7 in cells.

Cyanimide-based compounds are reported to potently inhibit deubiquitinating enzymes (DUBs).^17−19^ We therefore investigated the inhibitory potential of our compounds against DUBs in cells using the fluorescent activity-based DUB probe Rho-Ub-PA (Supporting Information Figure S3).^20^ For this purpose, we used the HEK293T cell line for which the activity-based DUB profiling has been well-established and used to assess DUB inhibitors.^17−19,21^ Only 4 out of the 11 compounds (**108**, **199**, **334**, and **415**) showed inhibition of one or more DUBs, mainly UCHL1, while the others, including our most potent compounds **84**, **336**, and **340**, did not show interference with the activity of any DUB. Based on the potency and selectivity results, we selected compounds **84**, **336**, and **340** and further assessed their binding to cellular PARK7 in a dose-dependent manner. HEK293T cells were treated with a 0.1–5 μM dilution series of these inhibitors for 24 h, followed by cell lysis and incubation with probe **JYQ-92**. Fluorescence scanning and Western blotting revealed that compounds **84** and **340** engaged PARK7 from 0.1 μM, while compound **336** was less potent and engaged PARK7 activity from 1 μM (Figure 2C). Hence, it was decided to continue with the most potent, cell-permeable compounds **84** and **340**, which were renamed **JYQ-173** and **JYQ-164**, respectively, for further experiments and to ease future reference (Figure 1G). As expected, based on our previous data for **JYQ-88**, both **JYQ-164** and **JYQ-173** bind covalently with PARK7 as evidenced by intact protein mass spectrometry (Supporting Information Figure S4). The ability of **JYQ-164** and **JYQ-173** to inhibit PARK7 enzymatic activity was assessed using the DiFMUAc assay reagent,^13^ along with **JYQ-88** and the previously reported isatin-based PARK7 inhibitor **STK793590**.^11,13^ This assay relies on the PARK7 active-site cysteine106-dependent deacetylation of the fluorogenic substrate 6,8-difluoro-4-methylumbelliferyl, thereby mimicking the glyoxalase activity of PARK7. **JYQ-164** and **JYQ-173** potently inhibited PARK7 activity with IC_50_ values of 21 and 19 nM, respectively (Supporting Information Figure S5A), showing a 5-fold improved potency compared with **JYQ-88** (IC_50_ 120 nM) and **STK793590** (IC_50_ 130 nM). These inhibitors also showed increased selectivity toward PARK7 compared to UCHL1 with a nearly 1000-fold difference (Supporting Information Figure S5B and Table S1). After showing the selectivities and potencies of **JYQ-164** and **JYQ-173** toward PARK7 in HEK293T cells, we conducted further experiments in A549 cells, a model cell line of lung adenocarcinoma and used as a model in previous PARK7 studies.^14,22−26^ Both compounds did not show any cytotoxicity in A549 cells up to 5 μM after 72 h incubation but still showed complete inhibiton of PARK7 engagement with the **JYQ-92** probe (Supporting Information Figure S6).

### **JYQ-164** and **JYQ-173** Are Highly Selective Inhibitors for PARK7

**Figure 3:**
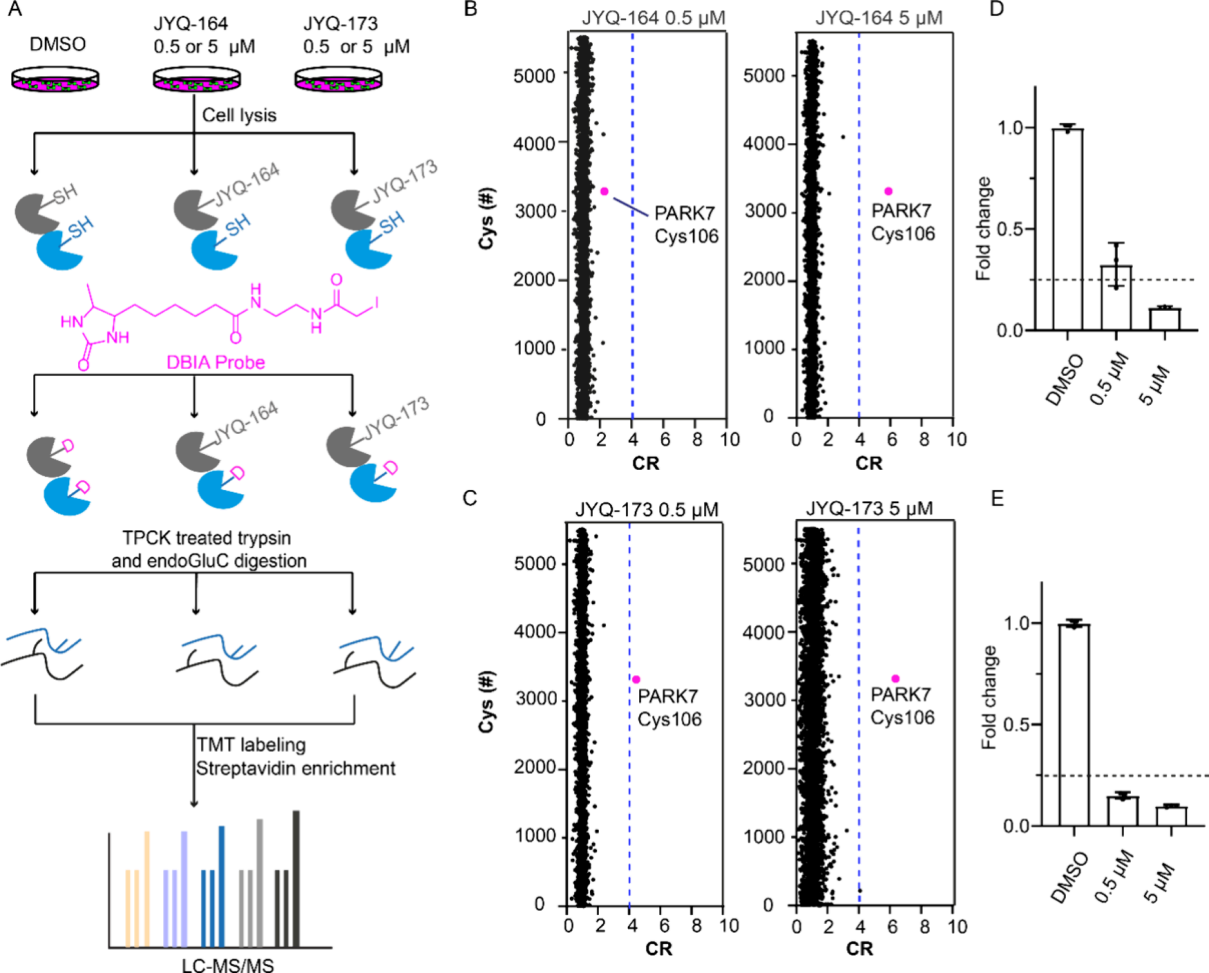
Investigation of the cellular selectivities of **JYQ-164** and **JYQ-173**. (A) Schematic illustration of the workflow for the SLC-ABPP experiment. (B, C) SLC-ABPP profiling of PARK7 inhibitor **JYQ-164** or **JYQ-173** in A549 cells using two different concentrations (0.5 and 5 μM) quantified >5500 cysteine sites. All experiments were performed in triplicates. Data are represented as means ± s.d. Dotted lines represent a CR threshold of 4 (75% reduction in DBIA probe binding). (D, E) Fold changes of annotated peptide for PARK7 Cys106 residue in inhibitor-treated samples compared to a DMSO control are represented as a column graphic where the 0.25 fold change corresponding to 75% reduction in DBIA probe binding is highlighted with dotted lines.

To investigate the selectivities of **JYQ-164** and **JYQ-173** in cells, we performed a streamlined cysteine activity-based protein profiling (SLC-ABPP) that can be used to profile and quantify the reactive cysteines binding to covalent inhibitors.^16^ A549 cells were treated with 0.5 or 5 μM of each inhibitor for 4 h along with a DMSO control, followed by cell lysis and incubation with a desthiobiotin iodoacetamide (DBIA) probe that is used to differentiate and enrich reactive cysteine sites that are not bound to **JYQ-164** or **JYQ-173**. Further, the samples were digested with tosylsulfonyl phenylalanyl chloromethyl ketone (TPCK)-treated trypsin and endoGluC to increase the coverage of protein sequences, especially the PARK7 peptide containing Cys106 residue. Peptides generated by digestion from replicates of each sample were labeled using TMT16-plex to perform tandem mass tag (TMT)-based quantitative proteomics profiling. Following the TMT labeling, all samples were pooled and the peptides conjugated to the DBIA probe were enriched by streptavidin beads and analyzed by LC-MS/MS (Figure 3A). A total of 13,492 peptides containing cysteine sites were detected and listed according to their abundance ratios for all the conditions (Supplementary Data S2). With the aim of representing our data, competition ratio (CR) values for each modified peptide were first calculated as the abundance ratios between each replicate of different concentrations of **JYQ-164** and **JYQ-173** and the average abundance ratios of the DMSO samples for the same peptide (DMSO/inhibitor ratio). All cysteines were represented with the shortest peptide by using the average CR values of all the peptides with the same modified cysteine from the same protein in the same condition.^16,27,28^ A total of 5512 unique cysteine sites were quantified in this analysis (Supplementary Data S3). Only the cysteines with a competitive ratio ≥4 (named CR threshold), corresponding to ≥75% reduction of DBIA probe alkylation at the cysteine by **JYQ-164** or **JYQ-173**, were considered as targeted (Figure 3B–E). Following treatment with 5 μM **JYQ-164**, PARK7 Cys106 was identified as the only targeted site, while no targeted site that showed ≥75% reduction of DBIA probe alkylation was identified with the treatment of 0.5 μM **JYQ-164** (Figure 3B,D). In addition, we identified PARK7 Cys106 as the only targeted cysteine that showed ≥75% reduction of DBIA probe alkylation in **JYQ-173**-treated cells (at 0.5 and 5 μM) (Figure 3C,E). Together, these data demonstrate that **JYQ-164** and **JYQ-173** are highly selective to PARK7 Cys106, and **JYQ-173** has a higher potency than **JYQ-164** toward PARK7 in cells.

### **JYQ-164** and **JYQ-173** Turn into Cell-Permeable Activity-Based Probes

**Figure 4:**
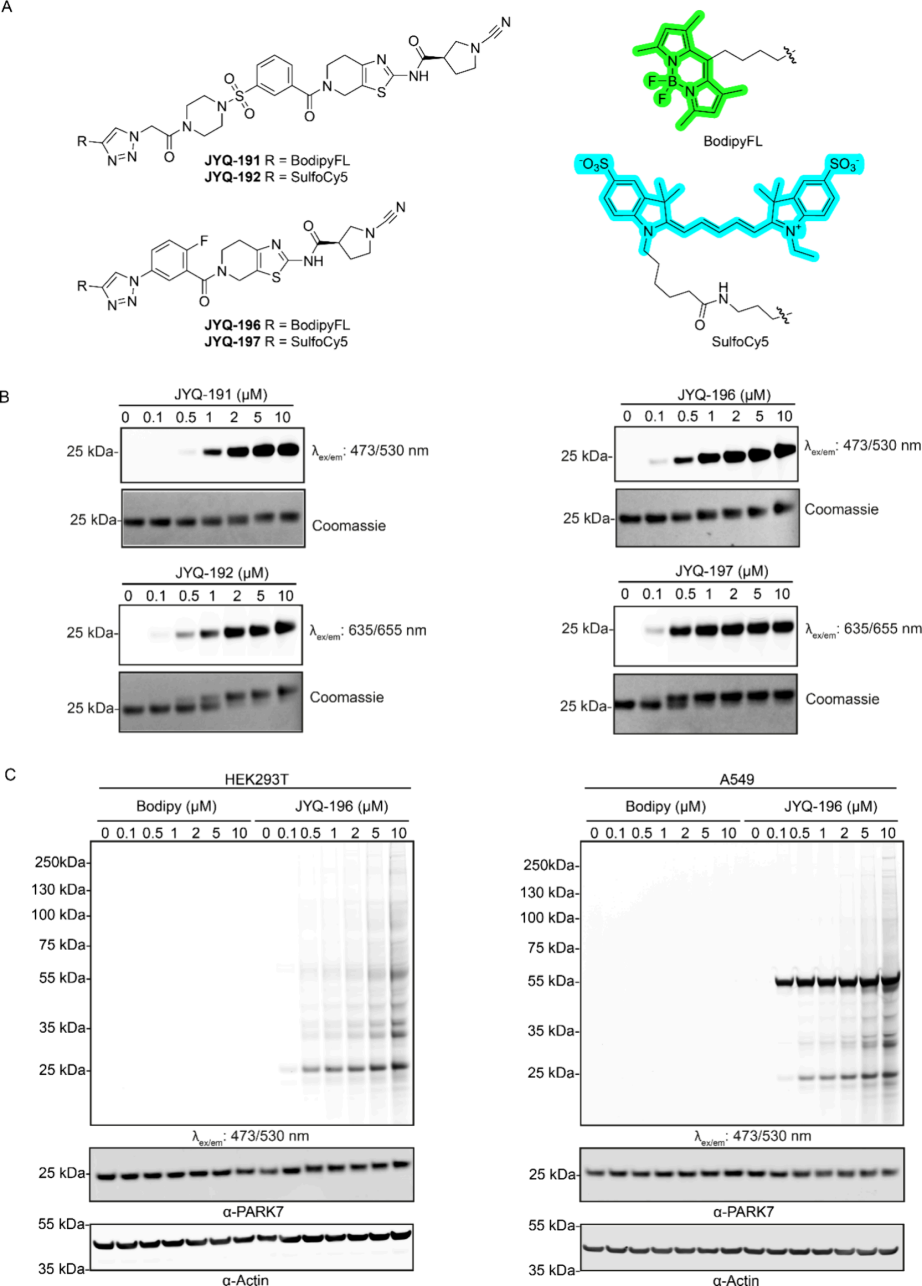
(A) Chemical structures of activity-based probes **JYQ-191**, **JYQ-192**, **JYQ-196**, and **JYQ-197**. (B) Labeling of purified recombinant human PARK7 with the four probes. Recombinant human PARK7 was incubated with indicated concentrations of the probes for 1 h followed by SDS-PAGE, fluorescence scanning, and coomassie staining. (C) Fluorescence labeling of PARK7 activity in HEK293T and A549 cells with **JYQ-196**. HEK293T and A549 cells were incubated with indicated final concentration of **JYQ-196** for 4 h, followed by cell lysis, SDS-PAGE, fluorescence scanning, and immunoblotting against PARK7 and β-actin. β-Actin was used as a loading control.

**Scheme 2:**
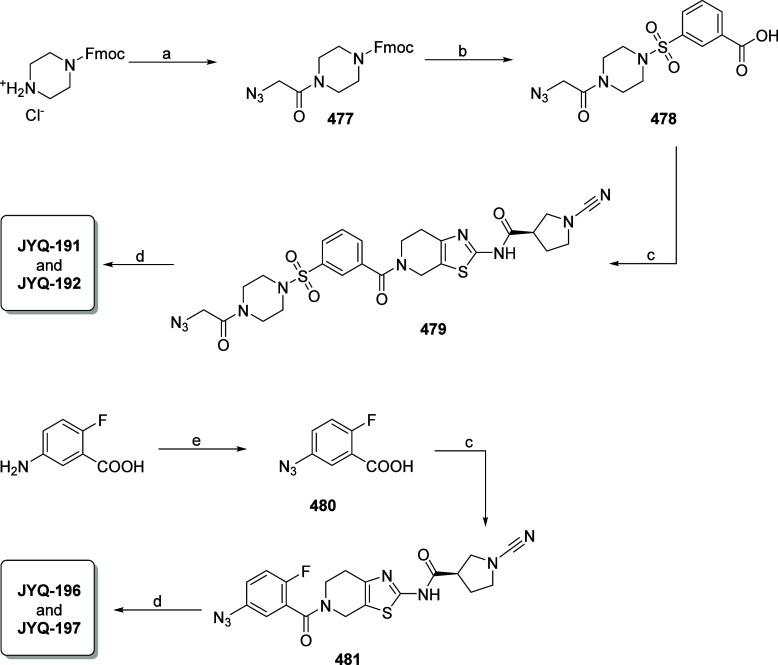
Synthesis of Fluorescent PARK7 Probes Reagents and conditions: (a) azidoacetic acid, HCTU, DIPEA, DCM; (b) (i) DBU, DCM; (ii) 3-(chlorosulfonyl)benzoic acid, DIPEA, DCM; (c) (*R*)-1-cyano-*N*-(4,5,6,7-tetrahydrothiazolo[5,4-*c*]pyridin-2-yl)pyrrolidine-3-carboxamide,^15^ HCTU, DIPEA, DCM; (d) CuSO_4_·5H_2_O, sodium ascorbate, BodipyFL-alkyne or sulfoCy5-alkyne, DMF; (e) NaNO_2_, NaN_3_, H_2_SO_4_, H_2_O, 0 °C.

With the confirmation that **JYQ-164** and **JYQ-173** are highly potent and selective for cellular PARK7, we opted to convert our inhibitors into fluorescent, cell-permeable activity-based probes (ABPs), by installing a fluorescent dye. Because of our successful experience previously, we chose to install SulfoCy5-alkyne and BodipyFL-alkyne via the copper(I)-catalyzed azide–alkyne cycloaddition (or click) reaction. We took advantage of the triazole group already present in **JYQ-173**. A new compound was synthesized, where we replaced the triazole moiety in **JYQ-173** with an azide, which was converted into the corresponding triazole after the click reaction with both alkynes, resulting in probes **JYQ-196** and **JYQ-197** (Figure 4A, Scheme 2). A convenient point of attachment was not readily available for inhibitor **JYQ-164**. We therefore substituted its morpholine moiety with 4-azidoacetyl piperazine and reacted this with both alkynes to obtain probes **JYQ-191** and **JYQ-192** (Figure 4A, Scheme 2). The ability of these probes to label and visualize PARK7 activity *in vitro* was evaluated by treating recombinant human PARK7 protein (1 μM) with a concentration series of the probes for 1 h at 37 °C, followed by SDS-PAGE analysis under nondenaturing conditions (without boiling and β-mercaptoethanol). Fluorescence scanning of the gel clearly showed that all four probes could label recombinant PARK7 in a dose-dependent manner. PARK7 labeling with probes **JYQ-196** and **JYQ-197** based on inhibitor **JYQ-173** was more efficient than the probes **JYQ-191** and **JYQ-192** based on inhibitor **JYQ-164** (Figure 4B). Taking the advantage of a band shift caused by the Bodipy and SulfoCy5 probes, we could also follow the complete labeling of recombinant PARK7 by these probes. Coomassie staining showed that **JYQ-196** and **JYQ-197** completely labeled recombinant PARK7 protein at 2 and 1 μM concentration, respectively. On the other hand, **JYQ-191** reached complete labeling at 5 μM and **JYQ-192** at 2 μM concentration, which implies a higher labeling efficiency of the **JYQ-173**-based probes than the **JYQ-164**-based probes (Figure 4B).

To further investigate the ability of these probes to visualize PARK7 in cells, HEK293T and A549 cells were incubated with 5 μM of these probes for 24 h, followed by cell lysis, SDS-PAGE, fluorescence scanning, and immunostaining (Supporting Information Figure S7). A clear band around 25 kDa was observed for both SulfoCy5 probes (**JYQ-192** and **JYQ-197**) and BodipyFL probe **JYQ-196**, corresponding to the expected mass of ABP-labeled PARK7. We also observed a band shift of the PARK7 protein in the samples treated with **JYQ-192, JYQ-196**, and **JYQ-197** by immunoblot against PARK7 in A549 cells. On the other hand, we did not observe any shift of the PARK7 protein in the sample treated with Bodipy probe **JYQ-191**, indicating inefficient labeling of cellular PARK7 by this probe. Therefore, we further investigated **JYQ-192, JYQ-196**, and **JYQ-197**. To characterize their cell permeability, A549 cells were incubated with 5 μM final concentration of these probes for the indicated time points, followed by confocal microscopy and by cell lysis, SDS-PAGE, fluorescence scanning, and immunostaining. All three probes already entered the cells after 1 h incubation (Supporting Information Figure S8A). However, only Bodipy probe **JYQ-196** showed complete labeling of cellular PARK7 after 4 h incubation, while SulfoCy5 probes **JYQ-192** and **JYQ-197** labeled a small portion of cellular PARK7 even after 24 h incubation (Supporting Information Figure S8B), suggesting poor target engagement of **JYQ-192** and **JYQ-197** in intact cells. Besides PARK7, we observed extra bands in the fluorescence scan for both HEK293T and A549 cells and a very strong band labeling between 55 and 75 kDa in A549 cells with a 5 μM final concentration of all probes.

Despite the fact that probe **JYQ-196** outperformed SulfoCy5 probes **JYQ-192** and **JYQ-197** in terms of the cellular PARK7 labeling efficiency, this probe still lacked specificity toward PARK7. PARK7 is known to form dimers, trimers, and even high-molecular-weight complexes under nonreducing conditions.^29,30^ Hence, to examine whether these extra bands are labeling of different forms of PARK7, PARK7 was depleted in HEK293T and A549 cells via siRNA transfection for 48 h and PARK7-depleted and control cells were subsequently incubated with 5 μM final concentration of **JYQ-196** for an adiditional 4 h. Disapparence of a clear band corresponding to PARK7 was observed in the PARK7-depleted samples, while other bands did not show any changes compared to control samples (Supporting Information Figure S9), suggesting unspecific labeling of other proteins with **JYQ-196**. To investigate further whether lower concentrations of **JYQ-196** can decrease unspecific labeling, HEK293T and A549 cells were incubated with increased concentrations (0–10 μM) of **JYQ-196** for 4 h, followed by cell lysis, SDS-PAGE, fluorescence scanning, and immunostaining (Figure 4C). This showed that a lower concentration of **JYQ-196** can still efficiently label PARK7 while showing less unspecific labeling.

### PROTAC Derived from **JYQ-173** Provides a Functional Cellular PARK7 Degrader

**Figure 5:**
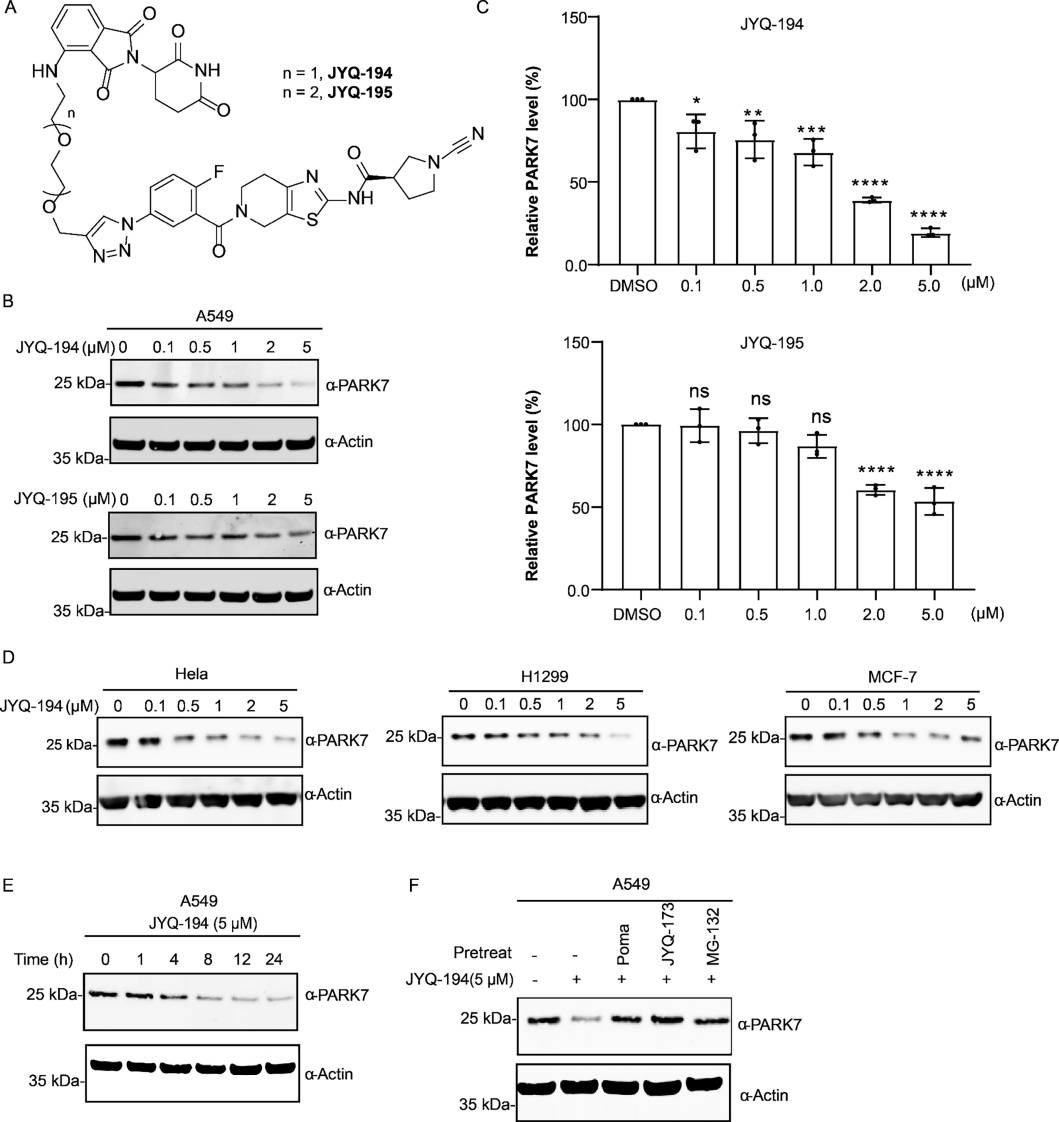
(A) Structures of PROTACs **JYQ-194**, **JYQ-195**. (B) PARK7 degradation efficacy with PROTACs **JYQ-194**, **JYQ-195**. A549 cells were incubated with the indicated concentration of PROTACs **JYQ-194** and **JYQ-195** for 8 h, followed by cell lysis, SDS-PAGE, and immunoblot analysis. (C) Quantification of Western blot of **JYQ-194**, **JYQ-195** in panel B. Total PARK7 protein levels at each concentration of **JYQ-194** were normalized to a DMSO control. Quantified data represent mean ± SD for three independent biological replicates. All significance was calculated using standard Student’s *t* test: *p <0.05, **p <0.01, ***p <0.001. (D) **JYQ-194** induces PARK7 degradation in multiple tumor cell lines. Cells were treated with indicated concentrations of **JYQ-194** for 8 h, followed by cell lysis and immunoblot analysis. (E) Time-course experiment for **JYQ-194** (5 μM). A549 cells were treated with **JYQ-194** (5 μM) for the indicated time, followed by cell lysis, running SDS-PAGE gel, and immunoblot analysis. (F) PARK7 degradation relies on ternary complex formation and proteasomal degradation. A549 cells were pretreated with 5 μM CRBN binder pomalidomide (POMA), 5 μM PARK7 inhibitor **JYQ-173**, or proteasome inhibitor 10 μM MG-132 for 4 h, followed by treatment with **JYQ-194** (5 μM) for additional 8 h. Following cell lysis, the samples were run in SDS-PAGE gel, and immunoblot analysis was performed. All immunoblots performed in this figure were against PARK7 and β-actin. β-Actin was used as a loading control.

**Scheme 3:**
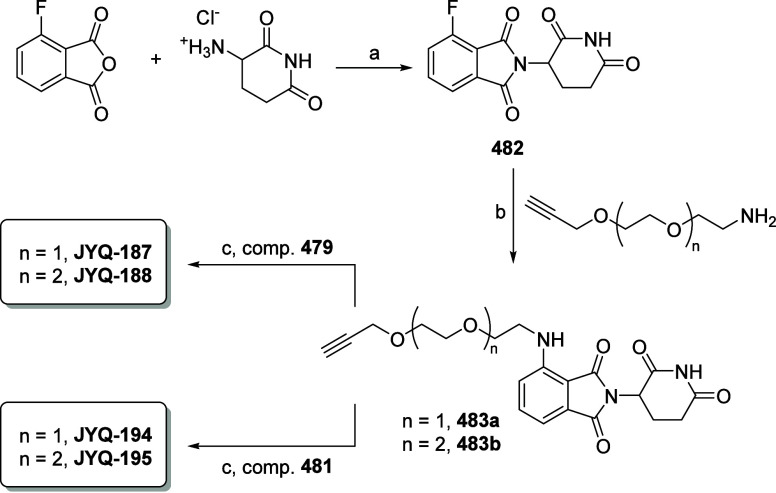
Synthesis of PARK7 PROTACs Reagents and conditions: (a) NaOAc, AcOH, reflux; (b) TEA, DMF; (c) CuSO_4_·5H_2_O, sodium ascorbate, DMF.

After having successfully converted our inhibitors into cell-permeable ABPs, we investigated the option of generating proteolysis targeting chimeras (PROTACs). PROTACs are heterobifunctional molecules that bind with an E3 ligase and protein of interest (POI) to form a ternary complex, enabling the degradation of the POI by targeting it to the proteasome.^31,32^ As such, we designed and synthesized a small set of PROTACs, **JYQ-187** and **JYQ-188** (based on inhibitor **JYQ-164**), and **JYQ-194** and **JYQ-195** (based on inhibitor **JYQ-173**), in a similar approach as for the above-described ABPs, yet this time conjugating the cereblon ligand pomalidomide, via two PEG linkers of different length (Figure 5A, Scheme 3, Supporting Information Figure S10A). We assessed their ability to induce PARK7 degradation in cells by treating A549 cells with increasing concentrations (from 0.1 to 5 μM) of the PROTACs for 8 h. Of them, **JYQ-194** stood out as the most potent PARK7 degrader by inducing degradation of PARK7 starting at 0.1 μM and reaching a *D*_max_ of ∼80% at a concentration of 5 μM (Figure 5B,C). **JYQ-195** was less efficient than **JYQ-194** as it degraded PARK7 starting at 2 μM. No degradation of PARK7 was observed for **JYQ-187** and **JYQ-188**, both of which are based on **JYQ-164** (Supporting Information Figure S10B). Therefore, **JYQ-194** was selected for further evaluation.

Next, we determined the efficiency of **JYQ-194**-induced PARK7 degradation in multiple tumor cell lines, including Hela, H1299, and MCF7. PARK7 degradation was observed in all these cells in a dose-dependent manner (Figure 5D). To explore **JYQ-194**-induced PARK7 degradation kinetics in cells, we performed a time-course experiment in A549 cells using a fixed concentration of 5 μM **JYQ-194**, which had shown to induce maximal degradation among the tested concentrations in all cell lines we tested. Degradation of PARK7 was observed starting from 4 h, and the maximal degradation of PARK7 was reached by 8 h and remained until 24 h (Figure 5E). To test the functionality of each component of the **JYQ-194** PROTAC and to investigate whether PARK7 degradation indeed depends on the ubiquitin-proteasome system, we pretreated A549 cells with the CRBN binder pomalidomide,^33^ the PARK7 inhibitor **JYQ-173**, or the proteasome inhibitor MG-132 for 4 h,^34^ followed by treatment with **JYQ-194** for additional 8 h. The degradation of PARK7 is abolished by each inhibitor targeting an individual component of the functional PROTAC and the proteasome (Figure 5F), suggesting ubiquitin/proteasome system-dependent PARK7 degradation via **JYQ-194**.

### **JYQ-194** Selectively Induces PARK7 Degradation

**Figure 6:**
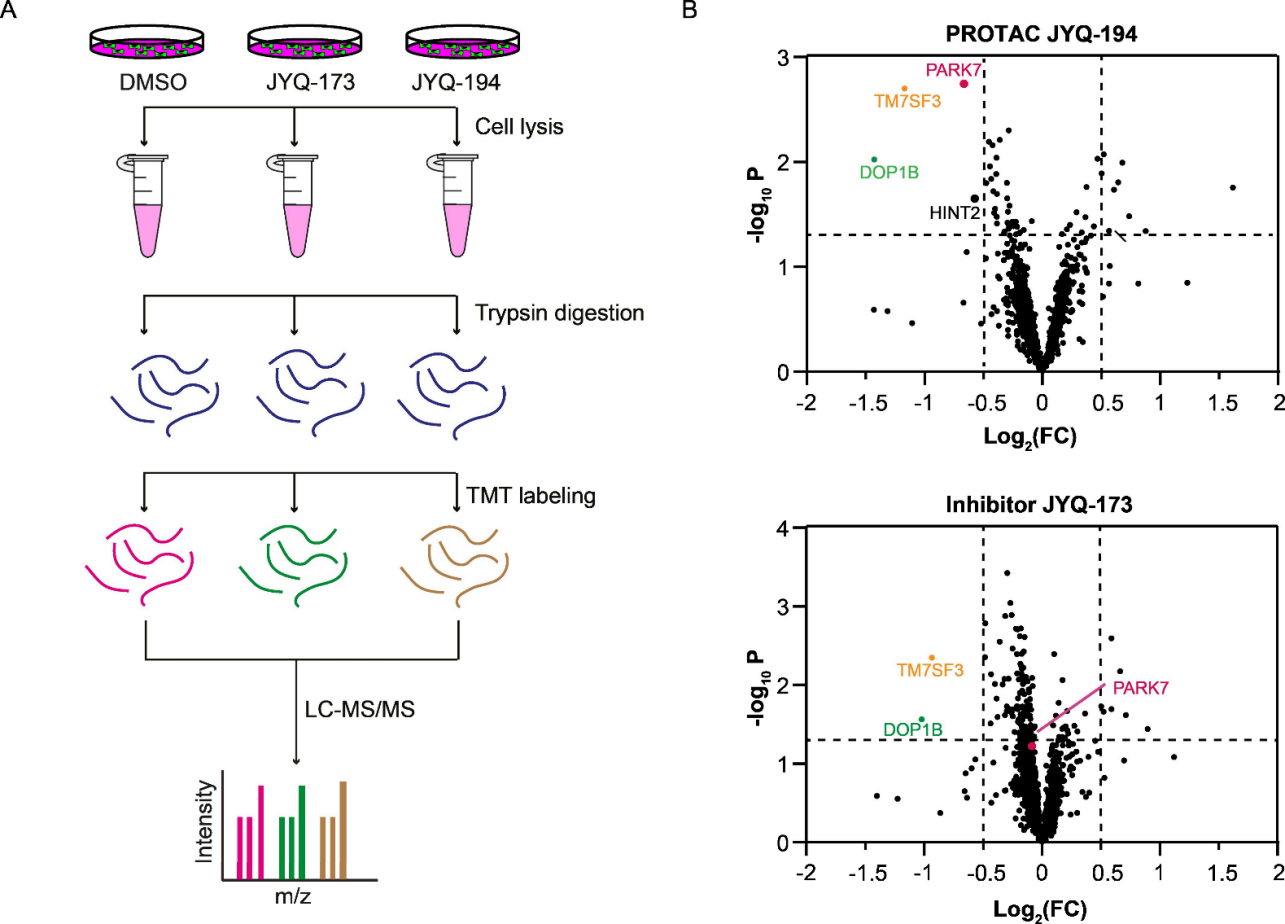
(A) Schematic illustration of the workflow of TMT-based total proteomics experiments. A549 cells were incubated with DMSO, **JYQ-173**, or **JYQ-164** for 8 h. Upon cell lysis and trypsin digestion, the peptides were labeled with TMTpro label reagents, and samples were pooled for LC-MS/MS analysis. (B) Proteomics analysis of proteins differentially expressed in PROTAC **JYQ-194** and inhibitor **JYQ-173**-treated A549 cells compared to DMSO-treated A549 cells. Volcano plots of the −log_10_ (*p-*value) versus the log_2_ fold change (FC) for **JYQ-194** versus DMSO (top panel) and **JYQ-173** versus DMSO (bottom panel). All the samples were prepared as *n* = 3 biological replicates. Proteins with −log_10_ (*p-*value) > 1.3 and log_2_ FC < −0.5 or log_2_ FC > 0.5 were considered to be significantly changed in abundance. *p*-Values were calculated using standard Student’s *t*-test.

To explore the degradation selectivity of PROTAC **JYQ-194** toward PARK7, we performed a quantitative TMT-based total proteome profiling by incubating A549 cells with 5 μM **JYQ-194** for 8 h, along with 5 μM **JYQ-173** and DMSO as a control (Figure 6A). Over 6000 proteins were identified in the samples (Supplementary Data S4). Notably, PROTAC **JYQ-194** induced the degradation of PARK7. As expected, **JYQ-173** did not change the PARK7 protein level (Figure 6B). The proteomic data also revealed TM7SF3 (transmembrane 7 superfamily member 3) and DOP1B (DOP1 leucine zipper-like protein B) to be decreased by PROTAC **JYQ-194**, while these proteins were also downregulated by inhibitor **JYQ-173** (Figure 6B), suggesting that downregulation of these two proteins is associated with Cys106-dependent functions of PARK7. In addition, PROTAC **JYQ-194**, unlike inhibitor **JYQ-173**, partly decreased HINT2 (histidine triad nucleotide-binding protein 2), implying that HINT2 might be an off-target of PROTAC **JYQ-194** or might be regulated by scaffold function of PARK7. PARK7 still remains the major target of **JYQ-194**. Collectively, these findings revealed that **JYQ-194** is a selective degrader of PARK7.

## Conclusions

Besides serving as potential therapeutic agents, small-molecule inhibitors are great tools to investigate the biological function of specific proteins, especially enzymes, by binding to them via catalytic or allosteric mechanisms and affecting their cellular functions.^34^ These inhibitors have also provided a foundation to design and extend chemical tools such as activity-based probes (ABPs) and protein degraders (PROTACs). Combining these chemical tools derived from similar structures can help study diverse perspectives of target proteins *in vitro*, in living cells, and even in complex animal models.^35−37^ By using our recently developed high-throughput screening compatible FP assay,^15^ herein, we report the development of highly potent and selective inhibitors **JYQ-164** and **JYQ-173** for an attractive therapeutic target, PARK7.^1,38−42^ Both compounds potently inhibited PARK7 enzymatic activity with IC_50_ values of 19 and 21 nM, respectively, showing a 5-fold improved potency compared to our previously reported inhibitor **JYQ-88**. We identified these compounds in a chemical optimization study, which involved the replacement of the 2-azidoacetyl moiety in **JYQ-88** by a large variety of substituents with the goal to fill the largely unoccupied protein pocket surrounding this moiety. The *in vitro* FP assay on our panel of 15 purified compounds showed that the introduction of an aromatic substituent was beneficial for the inhibitory potency, whereas a nonaromatic, hydrophilic moiety did not improve (compounds **1** and **2**) or even lowered (compounds **4** and **5**) the potency. Interestingly, all compounds that showed improved inhibition contain a *meta*-substituted aromatic moiety, which provides some possible insights into the binding mode within the protein pocket of these compounds. Notably, we showed direct target engagement of inhibitors **JYQ-164** and **JYQ-173** in live cells in a cell-based competition assay. In-cell SLC-ABP profiling revealed that these compounds specifically target PARK7’s highly conserved Cys106 residue that is essential for the biological functions of PARK7 and sensitive to oxidative stress.^2^

With these potent and selective inhibitors in hand, we designed and synthesized four activity-based probes, **JYQ-191**, **JYQ-192**, **JYQ-196**, and **JYQ-197**, by installment of Bodipy or a SulfoCy5 dyes onto them. Probe **JYQ-196**, derived from **JYQ-173** by installing Bodipy dye, outperformed other probes by presenting efficient labeling of PARK7 activity in living cells. However, **JYQ-196** also showed unspecific labeling of other proteins in the labeling experiment, while the original compound, **JYQ-173**, showed high selectivity toward PARK7 in the SLC-ABPP experiment. The reason for the disagreement between the fluorescent labeling and SLC-ABPP experiments could be due to the changes in the chemical composition of **JYQ-173** by installment of dye as the similar effect observed in the previous studies for UCHL1-selective probes.^17,18,43^ Previously, Sieber and co-workers reported an alkyne-equipped probe to monitor PARK7 in different cell lines after a postlysis click reaction with rhodamine- or biotin-azide.^14^ The presented probes here provide great advantages because of the highly efficient one-step labeling of cellular PARK7 without a need for an intermediate click chemistry reaction, thus omitting all disadvantages associated with two-step labeling. Therefore, our probes could potentially serve as diagnostic tools with further endeavors, as PARK7 is a potential biomarker for various cancers and Parkinson’s diseases.^44−47^ In addition, we further developed a first-in-class selective PARK7 degrader **JYQ-194** that induced PARK7 degradation in different tumor cell lines. It should be noted that the covalent, irreversible nature of **JYQ-194** may limit its potency as it cannot be recycled upon PARK7 degradation, which possibly lowers its efficacy in an in *vivo* setting.^31^ Despite its limitation, the creation of **JYQ-194** PROTAC provides new opportunities for targeting PARK7 in cancer therapy since PARK7 is overexpressed in various types of cancer.^1,5,48^

In summary, we have largely expanded the chemical toolbox for PARK7 by the development of two cell-permeable, potent, and specific PARK7 inhibitors, two activity-based probes to monitor or visualize PARK7 activity in cells, and a selective degrader to induce PARK7 degradation. The potent and selective inhibitors, ABPs, and PROTAC provided by our study can be further investigated for their therapeutic potential, and these chemical tools would be helpful to shed light on the biological function of PARK7 in health and diseases and to explore possible future therapeutic agents for PARK7.

## Experimental Section

### Chemistry

All reagents and solvents were purchased from commercially available sources and used as received unless indicated otherwise. All reaction progress was monitored by thin-layer chromatography (TLC) under UV light or by using a solution of KMnO_4_ (7.5 g L^–1^) and K_2_CO_3_ (50 g L^–1^) in H_2_O and liquid chromatography–mass spectrometry (LC-MS). Compounds were purified by Büchi flash column chromatography (unless indicated otherwise) using GraceResolv Davisil silica with indicated eluents. NMR spectra (^1^H, ^13^C) were recorded on a Bruker Ultrashield 300 MHz spectrometer at 298 K. Resonances are indicated with symbols “d” (doublet), “s” (singlet), “t” (triplet), and “m” (multiplet). Chemical shifts (δ) are given in ppm relative to CDCl_3_, DMSO-*d*_6_, or CD_3_OD as an internal standard, and coupling constants (*J*) are quoted in hertz (Hz). LC-MS measurements were performed on an LC-MS system equipped with a Waters 2795 Separation Module (Alliance HT), a Waters 2996 Photodiode Array Detector (190–750 nm), an Xbridge C18 column (2.1 × 100 mm, 3.5 μm), and an LCT ESI-Orthogonal Acceleration Time of Flight Mass Spectrometer. Samples were run using 2 mobile phases: A = 1% CH_3_CN and 0.1% formic acid in H_2_O and B = 1% H_2_O and 0.1% formic acid in CH_3_CN. Data processing was performed using Waters MassLynx Mass Spectrometry Software 4.1. High-resolution mass spectra (HR-MS) were recorded on a Waters Acquity H-class UPLC with a UPLC BEH C18 column (1.7 μm, 2.1 × 50 mm) coupled to a Xevo G2-XS Qtof mass spectrometer with ESI. Preparative HPLC was performed on a Waters preparative automated HPLC with mass detection. Samples were run on an Xbridge PREP C18 column (5 μm, 19 × 150 mm) using base (NH_4_OH)-modified CH_3_CN/H_2_O gradients. Gradient: 0–2.5 min: 95% H_2_O, 5% CH_3_CN; 2.5–17.5 min: 5–40% CH_3_CN; 17.5–20.90 min: 40–95% CH_3_CN; 20.90–21.00 min: 95–5% CH_3_CN; 1 mL min^–1^ CH_3_CN (with 1% 4 M NH_4_OH) mixed through the eluent. All final compounds had a purity of >95% as confirmed by LC-MS and NMR.

### General Procedure A

To a solution of (*R*)-1-cyano-*N*-(4,5,6,7-tetrahydrothiazolo[5,4-*c*]pyridin-2-yl)pyrrolidine-3-carboxamide^15^ (1.0 equiv) in DCM were added HCTU (1.2 equiv) and DIPEA (3.6 equiv). The reaction mixture was stirred for 10 min. The indicated carboxylic acid (1.2 equiv) was then added, and the stirring was continued for 2 h at rt. The solvents were evaporated under reduced pressure, and the resulting residue was taken up in EtOAc. The organic layer was washed with 1 M HCl, sat. aq. NaHCO_3_, and brine, followed by drying with Na_2_SO_4_ and evaporating under reduced pressure. The resulting residue was purified by Büchi flash chromatography to obtain the desired product.

### General Procedure B

The first step was performed as general procedure A. The obtained product from general procedure A and DBU (0.5 equiv) were dissolved in DCM, and the reaction mixture was stirred at rt for 2 h. After the complete removal of the Fmoc group, the organic layer was evaporated under reduced pressure. The resulting residue was purified by Büchi flash chromatography.

#### (*R*)-1-Cyano-*N*-(5-(2-morpholinoacetyl)-4,5,6,7-tetrahydrothiazolo[5,4-*c*]pyridin-2-yl)pyrrolidine-3-carboxamide (**1**)

This compound was prepared according to the general procedure A using 2-morpholinoacetic acid (17.4 mg, 120 μmol, 1.2 equiv) as starting material. Purification after Büchi flash chromatography (DCM to 8% CH_3_OH/DCM) yielded **1** as a white solid (30 mg, 74.2 μmol, 74%). ^1^H NMR (300 MHz, DMSO-*d*_6_) δ 12.13 (d, *J* = 2.9 Hz, 1H), 4.58 (d, *J* = 44.9 Hz, 2H), 3.71 (dt, *J* = 11.4, 5.9 Hz, 2H), 3.58–3.28 (m, 9H), 3.20 (d, *J* = 7.3 Hz, 2H), 2.73–2.48 (m, 3H), 2.34 (s, 3H), 2.21–1.87 (m, 2H). ^13^C NMR (75 MHz, DMSO-*d*_6_) δ 170.8, 156.4, 143.3, 118.9, 117.5, 66.5, 53.4, 53.2, 52.5, 50.4, 43.5, 43.4, 29.7, 27.6, 26.4. HR-MS calculated for C_18_H_24_N_6_O_3_S [M + H]^+^ 405.1709, found 405.1703.

#### (*R*)-1-Cyano-*N*-(5-(2-(4-methylpiperazin-1-yl)acetyl)-4,5,6,7-tetrahydrothiazolo[5,4-*c*]pyridin-2-yl)pyrrolidine-3-carboxamide (**2**)

This compound was prepared according to the general procedure A using 4-methylpiperazine-1-carboxylic acid (18.9 mg, 120 μmol, 1.2 equiv) as starting material. Purification after Büchi flash chromatography (DCM to 10% CH_3_OH/DCM) yielded **2** as a white solid (20 mg, 47.9 μmol, 48%). ^1^H NMR (300 MHz, DMSO-*d*_6_) δ 4.65 (d, *J* = 43.7 Hz, 2H), 3.79–3.75 (m, 3H), 3.52–3.47 (m, 2H), 3.42 (ddt, *J* = 9.2, 5.9, 2.8 Hz, 3H), 3.36–3.22 (m, 3H), 2.67 (dt, *J* = 44.7, 5.9 Hz, 2H), 2.44 (d, *J* = 12.9 Hz, 6H), 2.23 (d, *J* = 11.5 Hz, 3H), 2.19–1.98 (m, 2H). ^13^C NMR (75 MHz, DMSO) δ 170.9, 168.7, 156.5, 143.4, 119.3, 119.0, 117.6, 60.9, 54.6, 52.5, 52.2, 50.4, 45.4, 43.1, 29.7, 27.5. HR-MS calculated for C_19_H_27_N_7_O_2_S [M + H]^+^ 418.2025, found 418.2019.

#### (*R*)-1-Cyano-*N*-(5-(4-hydroxybenzoyl)-4,5,6,7-tetrahydrothiazolo[5,4-*c*]pyridin-2-yl)pyrrolidine-3-carboxamide (**3**)

This compound was prepared according to the general procedure A using 3-hydroxybenzoic acid (16.6 mg, 120 μmol, 1.2 equiv) as starting material. Purification after Büchi flash chromatography (DCM to 6% CH_3_OH/DCM) yielded **3** as a white solid (22 mg, 55.3 μmol, 55%). ^1^H NMR (300 MHz, DMSO-*d*_6_) δ 12.24 (s, 1H), 9.91 (s, 1H), 7.38–7.23 (m, 2H), 6.92–6.76 (m, 2H), 4.66 (s, 2H), 3.75 (s, 2H), 3.56 (ddd, *J* = 29.7, 9.5, 6.9 Hz, 2H), 3.43 (td, *J* = 7.4, 5.0 Hz, 2H), 3.29 (d, *J* = 7.1 Hz, 1H), 2.73 (d, *J* = 7.5 Hz, 2H), 2.11 (ddt, *J* = 37.2, 12.8, 6.6 Hz, 2H). ^13^C NMR (75 MHz, DMSO) δ 170.8, 170.4, 159.5, 156.6, 143.5, 129.7, 126.5, 118.9, 117.6, 115.5, 52.5, 50.4, 43.5, 29.7. HR-MS calculated for C_19_H_19_N_5_O_3_S [M + H]^+^ 398.1287, found 398.1283.

#### (*R*)-1-Cyano-*N*-(5-(2-(piperazin-1-yl)acetyl)-4,5,6,7-tetrahydrothiazolo[5,4-*c*]pyridin-2-yl)pyrrolidine-3-carboxamide (**4**)

This compound was prepared according to the general procedure B using 4-(((9*H*-fluoren-9-yl)methoxy)carbonyl)piperazine-1-carboxylic acid (44.0 mg, 120 μmol, 1.2 equiv) as starting material. Purification after Büchi flash chromatography yielded **4** as a white solid (5.1 mg, 12.6 μmol, 13% over 2-steps). ^1^H NMR (300 MHz, CD_3_OD) δ 4.68 (d, *J* = 30.1 Hz, 2H), 3.87–3.77 (m, 2H), 3.62–3.55 (m, 2H), 3.53–3.38 (m, 3H), 3.31 (d, *J* = 12.7 Hz, 3H), 2.93 (d, *J* = 19.4 Hz, 3H), 2.77 (s, 1H), 2.65 (s, 1H), 2.61–2.49 (m, 4H), 2.16 (ddt, *J* = 20.1, 13.0, 6.3 Hz, 2H). ^13^C NMR (75 MHz, CD_3_OD) δ 187.5, 142.8, 119.0, 60.1, 52.2, 51.5, 51.2, 50.0, 44.2, 44.1, 43.6, 43.1, 42.8, 39.9, 39.6, 29.4, 26.9, 25.8. HR-MS calculated for C_18_H_25_N_7_O_2_S [M + H]^+^ 404.1869, found 404.1863.

#### (*R*)-1-Cyano-*N*-(5-(2-(piperidin-4-yl)acetyl)-4,5,6,7-tetrahydrothiazolo[5,4-*c*]pyridin-2-yl)pyrrolidine-3-carboxamide (**5**)

This compound was prepared according to the general procedure B using 1-(((9*H*-fluoren-9-yl)methoxy)carbonyl)piperidine-4-carboxylic acid (43.8 mg, 120 μmol, 1.2 equiv) as starting material. Purification after Büchi flash chromatography yielded **5** as white solid (4.8 mg, 11.9 μmol, 12% over 2-steps). ^1^H NMR (300 MHz, CD_3_OD) δ 4.64 (d, *J* = 2.2 Hz, 2H), 3.81 (dt, *J* = 16.5, 5.9 Hz, 2H), 3.59 (d, *J* = 7.0 Hz, 2H), 3.56–3.37 (m, 2H), 3.15 (s, 1H), 2.86–2.62 (m, 4H), 2.40 (dd, *J* = 11.2, 6.9 Hz, 2H), 2.26–2.05 (m, 2H), 1.98 (s, 1H), 1.84 (d, *J* = 13.2 Hz, 2H), 1.30 (s, 2H), 1.21 (s, 1H). HR-MS calculated for C_19_H_26_N_6_O_2_S [M + H]^+^ 403.1916, found 403.1911.

#### (*R*)-1-Cyano-*N*-(5-(3-(*N*-cyclopropylsulfamoyl)benzoyl)-4,5,6,7-tetrahydrothiazolo[5,4-*c*]pyridin-2-yl)pyrrolidine-3-carboxamide (**59**)

This compound was prepared according to the general procedure A using 3-(*N*-cyclopropyl sulfamoyl)benzoic acid (17.4 mg, 72 μmol, 1.2 equiv) as starting material. Purification after Büchi flash chromatography (DCM to 5% CH_3_OH/DCM) yielded **59** as a white solid (15.1 mg, 30.2 μmol, 50%). ^1^H NMR (300 MHz, CD_3_OD) δ 8.11–7.92 (m, 2H), 7.75 (d, *J* = 7.4 Hz, 2H), 4.61 (s, 2H), 4.12 (s, 1H), 3.80–3.41 (m, 6H), 2.85 (d, *J* = 18.3 Hz, 2H), 2.41–2.12 (m, 3H), 1.48–1.29 (m, 1H), 0.54 (dt, *J* = 12.1, 3.7 Hz, 4H). ^13^C NMR (75 MHz, DMSO-*d*_6_) δ 170.9, 168.8, 156.7, 141.1, 137.1, 131.2, 130.3, 128.5, 125.5, 118.6, 117.6, 52.5, 50.4, 43.5, 29.7, 24.6, 5.6. HR-MS calculated for C_22_H_24_N_6_O_4_S_2_ [M + H]^+^ 501.1379, found 501.1373.

#### (*R*)-1-Cyano-*N*-(5-(3-(morpholinosulfonyl)benzoyl)-4,5,6,7-tetrahydrothiazolo[5,4-*c*]pyridin-2-yl)pyrrolidine-3-carboxamide (**340**, **JYQ-164**)

This compound was prepared according to the general procedure A using 3-(morpholino sulfonyl)benzoic acid (19.5 mg, 72 μmol, 1.2 equiv) as starting material. Purification after Büchi flash chromatography (DCM to 5% CH_3_OH/DCM) yielded **340** as a light yellow solid (6.5 mg, 12.3 μmol, 20%). ^1^H NMR (300 MHz, DMSO-*d*_6_) δ 12.26 (s, 1H), 7.83 (dd, *J* = 21.7, 7.5 Hz, 4H), 4.79 (s, 1H), 4.57 (s, 1H), 3.63 (ddd, *J* = 9.7, 5.9, 3.5 Hz, 6H), 3.55–3.40 (m, 3H), 3.15 (qd, *J* = 7.3, 4.2 Hz, 2H), 2.95–2.88 (m, 4H), 2.80–2.68 (m, 2H), 2.30–2.00 (m, 2H). ^13^C NMR (75 MHz, DMSO-*d*_6_) δ 170.9, 156.7, 137.5, 135.4, 131.8, 130.5, 129.3, 126.3, 118.5, 117.5, 65.7, 52.5, 50.4, 46.4, 43.5, 42.3, 29.7. HR-MS calculated for C_23_H_26_N_6_O_5_S_2_ [M + H]^+^ 531.1484, found 531.1478.

#### (*R*)-*N*-(5-(4-Bromo-3-methylbenzoyl)-4,5,6,7-tetrahydrothiazolo[5,4-*c*]pyridin-2-yl)-1-cyanopyrrolidine-3-carboxamide (**415**)

This compound was prepared according to the general procedure A using 4-bromo-3-methylbenzoic acid (15.5 mg, 72 μmol, 1.2 equiv) as starting material. Purification after Büchi flash chromatography (DCM to 5% CH_3_OH/DCM) yielded **415** as a white solid (18.2 mg, 38.4 μmol, 64%). ^1^H NMR (300 MHz, DMSO-*d*_6_) δ 12.25 (s, 1H), 7.67 (d, *J* = 8.1 Hz, 1H), 7.46 (s, 1H), 7.21 (s, 1H), 4.66 (d, *J* = 52.4 Hz, 2H), 3.96–3.38 (m, 6H), 3.27–3.10 (m, 1H), 2.81–2.68 (m, 2H), 2.39 (s, 3H), 2.12 (ddd, *J* = 44.5, 12.9, 6.5 Hz, 2H). ^13^C NMR (75 MHz, DMSO-*d*_6_) δ 170.8, 156.6, 138.3, 136.0, 132.7, 129.9, 126.6, 126.1, 118.6, 117.5, 52.5, 50.4, 43.5, 42.3, 38.1, 29.7, 23.8, 22.8. HR-MS calculated for C_20_H_20_BrN_5_O_2_S [M + H]^+^ 474.0599, found 474.0595.

#### (*R*)-*N*-(5-(3-((4-Acetylpiperazin-1-yl)sulfonyl)benzoyl)-4,5,6,7-tetrahydrothiazolo[5,4-*c*]pyridin-2-yl)-1-cyanopyrrolidine-3-carboxamide (**336**)

This compound was prepared according to the general procedure A using 3-((4-acetylpiperazin-1-yl)sulfonyl)benzoic acid (20.3 mg, 65 μmol, 1.2 equiv) as starting material. Purification after Büchi flash chromatography (DCM to 5% CH_3_OH/DCM) yielded **336** as a white solid (11.7 mg, 20.4 μmol, 38%). ^1^H NMR (300 MHz, DMSO-*d*_6_) δ 12.25 (s, 1H), 7.84 (s, 2H), 7.78 (d, *J* = 7.0 Hz, 2H), 4.67 (d, *J* = 66.5 Hz, 2H), 3.97 (s, 1H), 3.67–3.39 (m, 9H), 2.94 (d, *J* = 15.8 Hz, 4H), 2.83–2.67 (m, 2H), 2.29–2.01 (m, 2H), 1.96 (s, 3H). ^13^C NMR (75 MHz, DMSO-*d*_6_) δ 170.9, 168.8, 166.0, 137.6, 135.8, 132.1, 130.6, 129.2, 126.2, 118.5, 117.6, 52.6, 50.4, 46.4, 46.2, 45.4, 43.5, 29.7, 21.6.HR-MS calculated for C_25_H_29_N7O_5_S_2_ [M + H]^+^ 572.1750, found 572.1754.

#### (*R*)-*N*-(5-(3-(Azepan-1-ylsulfonyl)-4-methoxybenzoyl)-4,5,6,7-tetrahydrothiazolo[5,4-*c*]pyridin-2-yl)-1-cyanopyrrolidine-3-carboxamid (**133**)

This compound was prepared according to the general procedure A using 3-(azepan-1-ylsulfonyl)-4-methoxybenzoic acid (20.4 mg, 65 μmol, 1.2 equiv) as starting material. Purification after Büchi flash chromatography (DCM to 5% CH_3_OH/DCM) yielded **133** as a white solid (10.5 mg, 18.4 μmol, 34%). ^1^H NMR (300 MHz, DMSO-*d*_6_) δ 12.26 (s, 1H), 7.82 (d, *J* = 1.6 Hz, 1H), 7.79–7.68 (m, 1H), 7.32 (d, *J* = 8.6 Hz, 1H), 4.69 (s, 2H), 3.96 (s, 3H), 3.91–3.36 (m, 6H), 3.26 (t, *J* = 5.7 Hz, 5H), 2.81–2.69 (m, 2H), 2.30–1.97 (m, 2H), 1.72–1.51 (m, 8H). ^13^C NMR (75 MHz, DMSO-*d*_6_) δ 170.8, 168.7, 157.9, 156.7, 133.9, 130.2, 127.7, 118.7, 117.6, 113.5, 56.9, 52.5, 50.4, 48.3, 43.5, 29.7, 29.4, 27.0. HR-MS calculated for C_26_H_32_N_6_O_5_S_2_ [M + H]^+^ 573.1954, found 573.1958.

#### (*R*)-*N*-(5-(3-(Azepan-1-ylsulfonyl)-4-methoxybenzoyl)-4,5,6,7-tetrahydrothiazolo[5,4-*c*]pyridin-2-yl)-1-cyanopyrrolidine-3-carboxamide (**55**)

This compound was prepared according to the general procedure A using 4-chloro-3-(1,1-dioxidoisothiazolidin-2-yl)benzoic acid (17.9 mg, 65 μmol, 1.2 equiv) as starting material. Purification after Büchi flash chromatography (DCM to 5% CH_3_OH/DCM) yielded **55** as a white solid (6.5 mg, 12.2 μmol, 23%). ^1^H NMR (300 MHz, DMSO-*d*_6_) δ 11.95 (s, 1H), 7.38 (t, *J* = 7.6 Hz, 2H), 7.24–7.10 (m, 1H), 4.45 (s, 1H), 4.28 (s, 1H), 3.48–3.38 (m, 6H), 3.12 (t, *J* = 7.3 Hz, 4H), 2.51 (s, 1H), 2.47–2.39 (m, 2H), 2.18–2.10 (m, 2H), 1.97–1.69 (m, 2H). ^13^C NMR (75 MHz, DMSO-*d*_6_) δ 171.0, 156.7, 135.7, 131.3, 128.5, 118.5, 117.6, 52.5, 50.3, 49.7, 47.3, 43.5, 40.6, 40.3, 29.7, 19.6. HR-MS calculated for C_22_H_23_ClN_6_O_4_S_2_ [M + H]^+^ 535.0989, found 535.0987.

#### (*R*)-1-Cyano-*N*-(5-(2-(thiophen-2-yl)thiazole-5-carbonyl)-4,5,6,7-tetrahydrothiazolo[5,4-*c*]pyridin-2-yl)pyrrolidine-3-carboxamide (**108**)

This compound was prepared according to the general procedure A using 2-(thiophen-2-yl)thiazole-5-carboxylic acid (13.7 mg, 65 μmol, 1.2 equiv) as starting material. Purification after Büchi flash chromatography (DCM to 5% CH_3_OH/DCM) yielded **108** as a white solid (5.7 mg, 12.1 μmol, 22%). ^1^H NMR (300 MHz, DMSO-*d*_6_) δ 12.27 (s, 1H), 8.19 (s, 1H), 7.83–7.78 (m, 2H), 7.22 (dd, *J* = 5.0, 3.8 Hz, 1H), 4.86 (s, 2H), 3.96 (t, *J* = 5.7 Hz, 2H), 3.65–3.40 (m, 4H), 3.31–3.11 (m, 1H), 2.78 (d, *J* = 27.1 Hz, 2H), 2.29–1.98 (m, 2H). ^13^C NMR (75 MHz, DMSO-*d*_6_) δ 170.9, 163.7, 161.0, 156.8, 143.4, 136.3, 130.7, 129.2, 118.5, 117.6, 52.5, 50.4, 43.5, 42.3, 29.7. HR-MS calculated for C_20_H_18_N_6_O_2_S_3_ [M + H]^+^ 471.0732, found 471.0718.

#### (*R*)-1-Cyano-*N*-(5-(3-(4-methylthiazol-2-yl)benzoyl)-4,5,6,7-tetrahydrothiazolo[5,4-*c*]pyridine-2-yl)pyrrolidine-3-carboxamide (**334**)

This compound was prepared according to the general procedure A using 3-(4-methylthiazol-2-yl) benzoic acid (14.2 mg, 65 μmol, 1.2 equiv) as starting material. Purification after Büchi flash chromatography (DCM to 5% CH_3_OH/DCM) yielded **334** as a white solid (6.3 mg, 13.2 μmol, 24%). ^1^H NMR (300 MHz, DMSO-*d*_6_) δ 12.25 (s, 1H), 8.02 (dt, *J* = 7.4, 1.7 Hz, 1H), 7.94 (s, 1H), 7.60 (t, *J* = 7.5 Hz, 2H), 7.39 (d, *J* = 1.1 Hz, 1H), 4.70 (d, *J* = 55.5 Hz, 2H), 3.97 (s, 1H), 3.72–3.39 (m, 6H), 2.84–2.67 (m, 2H), 2.44 (dd, *J* = 3.2, 1.0 Hz, 3H), 2.29–1.96 (m, 2H). ^13^C NMR (75 MHz, DMSO-*d*_6_) δ 170.9, 165.8, 153.9, 137.3, 133.9, 130.1, 127.6, 124.7, 118.7, 117.6, 115.8, 52.5, 50.4, 45.3, 43.5, 29.7, 17.4. HR-MS calculated for C_23_H_22_N_6_O_2_S_2_ [M + H]^+^ 479.1324, found 479.1319.

#### (*R*)-*N*-(5-(3-(1,2,4-Oxadiazol-3-yl)benzoyl)-4,5,6,7-tetrahydrothiazolo[5,4-*c*]pyridin-2-yl)-1-cyanop**y**rrolidine-3-carboxamide (**199**)

This compound was prepared according to the general procedure A using 3-(1,2,4-oxadiazol-3-yl)benzoic acid (13.7 mg, 72 μmol, 1.2 equiv) as starting material. Purification after Büchi flash chromatography (DCM to 5% CH_3_OH/DCM) yielded **199** as a white solid (5.9 mg, 13.2 μmol, 22%). ^1^H NMR (300 MHz, DMSO-*d*_6_) δ 12.26 (s, 1H), 9.77 (s, 1H), 8.19–8.11 (m, 1H), 8.07 (s, 1H), 7.71 (d, *J* = 6.0 Hz, 2H), 4.70 (d, *J* = 56.2 Hz, 2H), 3.98 (s, 1H), 3.68–3.38 (m, 5H), 3.30–3.13 (m, 1H), 2.83–2.65 (m, 2H), 2.31–1.95 (m, 2H). ^13^C NMR (75 MHz, DMSO-*d*_6_) δ 170.8, 168.2, 166.8, 156.7, 137.4, 130.3, 128.9, 126.7, 126.0, 118.6, 117.6, 52.5, 50.4, 43.5, 29.7. HR-MS calculated for C_21_H_19_N_7_O_3_S [M+H^]+^ 450.1348, found 450.1345.

#### (*R*)-1-Cyano-*N*-(5-(2-fluoro-5-(1*H*-1,2,3-triazol-1-yl)benzoyl)-4,5,6,7-tetrahydrothiazolo[5,4-*c*]pyridin-2-yl)pyrrolidine-3-carboxamide (**84**, **JYQ-173**)

This compound was prepared according to the general procedure A using 2-fluoro-5-(1*H*-1,2,3-triazol-1-yl)benzoic acid (14.9 mg, 72 μmol, 1.2 equiv) as starting material. Purification after Büchi flash chromatography (DCM to 5% CH_3_OH/DCM) yielded **84** as a white solid (7.8 mg, 16.7 μmol, 28%). ^1^H NMR (300 MHz, DMSO-*d*_6_) δ 12.26 (s, 1H), 8.86 (dd, *J* = 6.7, 1.2 Hz, 1H), 8.09 (dddd, *J* = 11.4, 5.6, 4.4, 2.8 Hz, 2H), 7.99 (dd, *J* = 3.3, 1.2 Hz, 1H), 7.70–7.51 (m, 1H), 4.70 (d, *J* = 92.9 Hz, 2H), 4.01 (t, *J* = 5.8 Hz, 1H), 3.68–3.49 (m, 5H), 3.35–3.25 (m, 1H), 2.73 (d, *J* = 25.2 Hz, 2H), 2.29–1.95 (m, 2H). ^13^C NMR (75 MHz, DMSO-*d*_6_) δ 170.9, 163.8, 157.4 (d, *J* = 247.3 Hz), 156.7, 143.5, 143.1, 135.1, 133.9, 125.7 (d, *J* = 20.5 Hz), 124.0, 123.7, 120.8 (d, *J* = 12.9 Hz), 118.3, 118.0, 117.6, 52.5, 50.3, 44.9, 43.5, 29.7, 27.3, 26.5. HR-MS calculated for C_21_H_19_FN_8_O_2_S [M + H]^+^ 467.1414, found 467.1417.

#### (9*H*-Fluoren-9-yl)methyl 4-(2-azidoacetyl)piperazine-1-carboxylate (**477**)

To a solution of *N*-(9-fluorenylmethoxycarbonyl)piperazine hydrochloride (1 g, 2.90 mmol, 1.0 equiv) in DCM were added HCTU (1.44 g, 3.48 mmol, 1.2 equiv) and DIPEA (1.72 mL, 10.44 mmol, 3.6 equiv). The reaction mixture was stirred for 10 min. Azidoacetic acid (262 μL, 3.48 mmol, 1.2 equiv) was then added, and stirring was continued for 2 h at rt. The solvents were evaporated under reduced pressure, and the resulting residue was taken up in EtOAc. The organic layer was washed with 1 M HCl, sat. aq. NaHCO_3_, and brine (50 mL), followed by drying with Na_2_SO_4_ and evaporating under reduced pressure. The resulting residue was purified by Büchi flash chromatography (DCM to 2% MeOH/DCM) to yield the title compound as a white solid (1.02 g, 2.61 mmol, 90%). ^1^H NMR (300 MHz, CDCl_3_) δ 7.80 (dt, *J* = 7.6, 1.0 Hz, 2H), 7.58 (dd, *J* = 7.4, 1.1 Hz, 2H), 7.48–7.40 (m, 2H), 7.39–7.32 (m, 2H), 4.56 (d, *J* = 6.2 Hz, 2H), 4.26 (t, *J* = 6.2 Hz, 1H), 3.96 (s, 2H), 3.64–3.18 (m, 8H). ^13^C NMR (75 MHz, CDCl_3_) δ 143.8, 141.4, 127.8, 127.1, 124.8, 120.1, 67.3, 50.8, 47.4, 45.0, 43.4, 41.7.

#### 3-((4-(2-Azidoacetyl)piperazin-1-yl)sulfonyl)benzoic Acid (**478**)

To a solution of compound **477** (1.0 g, 2.55 mmol, 1.0 equiv) in DCM (5 mL) was added DBU (190 μL, 1.23 mmol, 0.5 equiv), and the reaction mixture was stirred at rt for 2 h. After complete removal of the Fmoc group, 3-(chlorosulfonyl) benzoic acid (563.4 mg, 2.55 mmol, 1.0 equiv) and DIPEA (920 μL, 5.78 mmol, 2.2 equiv) were added. The resulting reaction mixture was stirred at rt for 0.5 h, followed by removing the solvents under reduced pressure. The crude material was taken up in EtOAc (50 mL), and the organic layer was washed with 1 M HCl (3 × 25 mL). The organic layer was dried over Na_2_SO_4_ and evaporated under reduced pressure. The resulting residue was used as such without further purification.

#### (*R*)-*N*-(5-(3-((4-(2-Azidoacetyl)piperazin-1-yl)sulfonyl)benzoyl)-4,5,6,7-tetrahydrothiazolo[5,4-*c*]pyridin-2-yl)-1-cyanopyrrolidine-3-carboxamide (**479**)

To a solution of (*R*)-1-cyano-*N*-(4,5,6,7-tetrahydrothiazolo[5,4-*c*]pyridin-2-yl) pyrrolidine-3-carboxamide (138.5 mg, 0.5 mmol, 1.0 equiv) in DCM were added HCTU (247.6 mg, 0.6 mmol, 1.2 equiv) and DIPEA (297 μL, 1.8 mmol, 3.6 equiv). The reaction mixture was stirred for 10 min. Compound **478** (212 mg, 0.6 mM, 1.2 equiv) was then added, and stirring was continued for 2 h at rt. The solvents were evaporated under reduced pressure, and the resulting residue was taken up in EtOAc (20 mL). The organic layer was washed with 1 M HCl (2 × 10 mL), sat. aq. NaHCO_3_ (3 × 10 mL), and brin**e** (10 mL), followed by drying with Na_2_SO_4_ and evaporating under reduced pressure. The resulting residue was purified by Büchi flash chromatography (DCM to 5% MeOH/DCM) to yield the title compound as a white solid (90 mg, 0.15 mmol, 25%). ^1^H NMR (300 MHz, DMSO-*d*_6_) δ 7.86 (d, *J* = 8.5 Hz, 2H), 7.78 (d, *J* = 7.0 Hz, 2H), 4.66 (d, *J* = 70.8 Hz, 2H), 4.10 (s, 3H), 3.57 (q, *J* = 11.5, 10.1 Hz, 8H), 3.35–3.21 (m, 2H), 2.96 (t, *J* = 4.9 Hz, 4H), 2.86–2.62 (m, 2H), 2.31–1.94 (m, 2H). ^13^C NMR (75 MHz, DMSO-*d*_6_) δ 171.0, 168.2, 166.5, 156.7, 137.5, 135.7, 132.1, 130.6, 129.2, 126.2, 118.5, 117.6, 52.6, 50.4, 50.1, 46.0, 43.7, 43.5, 29.7.

#### BodipyFL Probe **JYQ-191**

A solution of compound **479** (20.0 mg, 32.6 μmol, 1.0 equiv) and BodipyFL-alkyne (12.9 mg, 39.1 μmol, 1.2 equiv) in dry DMF (2 mL) was degassed by argon for 30 min. Aqueous solutions of sodium ascorbate (0.5 M) and CuSO_4_·5H_2_O (0.5 M) were prepared in 1 mL volume and degassed for 30 min with argon bubbling. The degassed sodium ascorbate (131 μL, 39.2 μmol, 1.2 equiv) and CuSO_4_·5H_2_O (112 μL, 32.6 μmol, 1.0 equiv) solutions were added to the reaction mixture, followed by stirring for 2 h. The resulting crude material was purified by preparative HPLC to yield **JYQ-191** as a red solid (7.5 mg, 8.0 μmol, 24%). ^1^H NMR (300 MHz, DMSO-*d*_6_) δ 12.25 (s, 1H), 7.82 (dt, *J* = 26.2, 7.6 Hz, 5H), 7.67 (s, 1H), 6.22 (s, 2H), 5.36 (s, 2H), 4.68 (d, *J* = 66.5 Hz, 2H), 3.97 (s, 1H), 3.56 (d, *J* = 17.6 Hz, 7H), 3.14–2.89 (m, 7H), 2.83–2.60 (m, 4H), 2.39 (s, 6H), 2.37 (s, 6H), 2.29–1.92 (m, 3H), 1.86–1.72 (m, 2H), 1.68–1.51 (m, 2H). ^13^C NMR (75 MHz, DMSO-*d*_6_) δ 170.9, 165.0, 153.5, 147.1, 146.6, 141.4, 137.6, 135.8, 132.1, 131.1, 130.6, 129.2, 126.2, 124.1, 122.1, 117.6, 52.5, 50.9, 50.4, 46.1, 43.9, 43.5, 31.2, 29.9, 29.6, 28.1, 25.0, 16.3, 14.5. HR-MS calculated for C_44_H_51_BF_2_N_12_O_5_S_2_ [M + H]^+^ 941.3694, found 941.3714.

#### SulfoCy5 Probe **JYQ-192**

This compound was prepared according to the procedure of **JYQ-191** using compound **479** (10.0 mg, 16.3 μmol, 1.0 equiv) and SulfoCy5-alkyne (14.2 mg, 19.6 μmol, 1.2 equiv) as starting materials. Purification by preparative HPLC yielded **JYQ-192** as a blue solid (6.2 mg, 4.7 μmol, 28.5%). ^1^H NMR (300 MHz, DMSO-*d*_6_) δ 12.25 (s, 1H), 8.35 (t, *J* = 13.1 Hz, 2H), 7.91–7.75 (m, 7H), 7.70–7.60 (m, 3H), 7.37–7.23 (m, 3H), 7.10 (s, 1H), 6.93 (s, 1H), 6.58 (t, *J* = 12.3 Hz, 1H), 6.30 (dd, *J* = 13.7, 2.3 Hz, 2H), 5.37 (s, 2H), 4.79 (s, 1H), 4.57 (s, 1H), 4.10 (q, *J* = 7.7 Hz, 4H), 3.66–3.50 (m, 7H), 3.10–2.96 (m, 6H), 2.80–2.70 (m, 2H), 2.60–2.54 (m, 2H), 2.17 (s, 1H), 2.08–1.98 (m, 3H), 1.73–1.65 (m, 15H), 1.54 (t, *J* = 7.3 Hz, 2H), 1.25 (t, *J* = 7.2 Hz, 4H). ^13^C NMR (75 MHz, DMSO-*d*_6_) δ 173.5, 173.1, 172.3, 170.9, 165.0, 156.7, 154.8, 145.7, 142.5, 142.0, 137.6, 135.8, 130.6, 129.2, 126.6, 126.2, 120.5, 117.6, 110.6, 110.4, 103.9, 103.6, 52.5, 50.9, 50.4, 49.4, 46.1, 43.5, 38.4, 35.6, 29.7, 29.5, 27.5, 27.4, 26.2, 25.4, 23.1, 12.6. HR-MS calculated for C_63_H_75_N_13_O_12_S_4_ [M + H]^2+^ 667.7349, found 667.7340.

#### 5-Azido-2-fluorobenzoic Acid (**480**)

To a solution of 5-amino-2-fluorobenzoic acid (300 mg, 1.93 mmol, 1.0 equiv) in a mixture of H_2_SO_4_ (2 mL) and H_2_O (10 mL) was added NaNO_2_ (133.4 mg, 1.93 mmol, 1.0 equiv) solution in H_2_O (1 mL) at 0 °C. The reaction mixture was stirred for 15 min. NaN_3_ (150.9 mg, 2.32 mmol, 1.2 equiv) solution in H_2_O (1 mL) was then added dropwise, and stirring was continued for 2 h at 0 °C. After the reaction was complete, the reaction mixture was extracted with EtOAc. The combined organic layer was washed with brine, followed by drying with Na_2_SO_4_ and evaporating under reduced pressure to yield the title compound as a white solid (285 mg, 1.57 mmol, 81%). ^1^H NMR (300 MHz, DMSO-*d*_6_) δ 7.25–7.21 (m, 1H), 7.14 (d, *J* = 1.7 Hz, 1H), 7.12 (d, *J* = 2.7 Hz, 1H).

#### (*R*)-*N*-(5-(5-Azido-2-fluorobenzoyl)-4,5,6,7-tetrahydrothiazolo[5,4-*c*]pyridin-2-yl)-1-cyanopyrrolidine-3-carboxamide (**481**)

To a solution of (*R*)-1-cyano-*N*-(4,5,6,7-tetrahydrothiazolo[5,4-*c*]pyridin-2-yl) pyrrolidine-3-carboxamide (138.5 mg, 0.5 mmol, 1.0 equiv) in DCM were added HCTU (247.6 mg, 0.6 mmol, 1.2 equiv) and DIPEA (297 μL, 1.8 mmol, 3.6 equiv). The reaction mixture was stirred for 10 min. Compound **480** (108.7 mg, 0.6 mmol, 1.2 equiv) was then added, and stirring was continued for 2 h at rt. The solvents were evaporated under reduced pressure, and the resulting residue was taken up in EtOAc (20 mL). The organic layer was washed with 1 M HCl (2 × 10 mL), sat. aq. NaHCO_3_ (3 × 10 mL), and brine (10 mL), followed by drying with Na_2_SO_4_ and evaporating under reduced pressure. The resulting residue was purified by Büchi flash chromatography (DCM to 5% MeOH/DCM) to yield the title compound as a white solid (180 mg, 0.40 mmol, 81%). ^1^H NMR (300 MHz, DMSO-*d*_6_) δ 12.26 (s, 1H), 7.43–7.32 (m, 1H), 7.32–7.12 (m, 2H), 4.85–4.43 (m, 2H), 3.97 (t, *J* = 5.9 Hz, 1H), 3.66–3.58 (m, 2H), 3.58–3.54 (m, 2H), 3.42 (d, *J* = 2.0 Hz, 1H), 3.36–3.07 (m, 1H), 2.82–2.59 (m, 2H), 2.28–1.97 (m, 2H). ^13^C NMR (75 MHz, DMSO-*d*_6_) δ 170.9, 164.1, 155.2 (d, *J* = 243.2 Hz), 143.6, 143.1, 136.8, 125.8 (d, *J* = 20.5 Hz), 122.7, 119.5, 118.3, 118.0 (d, *J* = 23.5 Hz), 117.6, 52.5, 50.4, 44.7, 43.5, 42.3, 29.7, 27.2.

#### BodipyFL Probe **JYQ-196**

This compound was prepared according to the procedure of **JYQ-191** using compound **481** (20 mg, 45.4 μmol, 1.0 equiv) and BodipyFL-alkyne (17.9 mg, 54.5 μmol, 1.2 equiv) as starting materials. Purification by preparative HPLC yielded **JYQ-196** as a red solid (8.2 mg, 10.7 μmol, 24%). ^1^H NMR (300 MHz, DMSO-*d*_6_) δ 12.17 (s, 1H), 8.66 (d, *J* = 7.8 Hz, 1H), 8.11–7.92 (m, 2H), 7.65–7.53 (m, 1H), 6.23 (d, *J* = 2.9 Hz, 2H), 4.85 (s, 1H), 4.54 (s, 1H), 4.00 (d, *J* = 5.9 Hz, 1H), 3.67–3.59 (m, 2H), 3.58–3.39 (m, 4H), 3.00 (d, *J* = 15.3 Hz, 2H), 2.91–2.63 (m, 5H), 2.55 (s, 1H), 2.40 (s, 12H), 2.30–1.97 (m, 3H), 1.90 (t, *J* = 7.3 Hz, 2H), 1.67 (s, 2H). ^13^C NMR (75 MHz, DMSO-*d*_6_) δ 170.9, 163.9, 157.3 (d, *J* = 247.1 Hz), 153.6, 148.4, 147.1, 141.3, 134.0, 131.2, 125.7 (d, *J* = 20.3 Hz), 122.2, 121.1, 118.3, 117.6, 52.6, 50.4, 44.8, 31.2, 29.6, 28.1, 27.3, 26.5, 25.0, 16.3, 14.5. HR-MS calculated for C_38_H_40_BF_3_N_10_O_2_S [M + H]^+^ 769.3187, found 769.3191.

#### SulfoCy5 Probe **JYQ-197**

This compound was prepared according to the procedure of **JYQ-191** using compound **481** (10 mg, 22.7 μmol, 1.0 equiv) and SulfoCy5-alkyne (19.7 mg, 27.2 μmol, 1.2 equiv) as starting materials. Purification by preparative HPLC yielded **JYQ-197** as a blue solid (6.1 mg, 5.3 μmol, 23%). ^1^H NMR (300 MHz, DMSO-*d*_6_) δ 12.24 (d, *J* = 13.9 Hz, 1H), 8.63 (d, *J* = 5.9 Hz, 1H), 8.35 (t, *J* = 13.1 Hz, 2H), 8.08–7.90 (m, 2H), 7.90–7.78 (m, 3H), 7.69–7.51 (m, 3H), 7.32 (dd, *J* = 8.2, 1.3 Hz, 2H), 7.28 (s, 1H), 7.11 (s, 1H), 6.94 (s, 1H), 6.58 (t, *J* = 12.4 Hz, 1H), 6.30 (d, *J* = 13.7 Hz, 2H), 4.84 (s, 1H), 4.53 (s, 1H), 4.19–3.97 (m, 5H), 3.67–3.58 (m, 2H), 3.57–3.46 (m, 3H), 3.10 (q, *J* = 6.6 Hz, 2H), 2.81–2.64 (m, 4H), 2.28–2.13 (m, 1H), 2.08–1.96 (m, 3H), 1.76 (dd, *J* = 5.9, 3.6 Hz, 2H), 1.68 (s, 12H), 1.55 (p, *J* = 7.3 Hz, 2H), 1.39–1.29 (m, 2H), 1.25 (t, *J* = 7.1 Hz, 3H). ^13^C NMR (75 MHz, DMSO-*d*_6_) δ 173.4, 173.1, 172.4, 170.9, 163.9, 163.7, 157.3 (d, *J* = 247.4 Hz), 156.9, 156.7, 154.8, 148.3, 145.7, 145.6, 143.6, 143.1, 142.5, 142.0, 141.1, 141.0, 134.0, 126.6, 126.3, 125.7 (d, *J* = 20.5 Hz), 121.0, 120.4, 118.4, 117.6, 110.6, 110.4, 103.9, 103.6, 52.5, 50.4, 49.3, 44.9, 43.9, 43.5, 38.4, 35.6, 29.7, 29.2, 27.4, 27.1, 26.2, 25.4, 23.0, 12.5. HR-MS calculated for C_57_H_64_FN_11_O_9_S_3_ [M + H]^2+^ 581.7095 found 581.7094.

#### 2-(2,6-Dioxopiperidin-3-yl)-4-fluoroisoindoline-1,3-dione (**482**)

To a solution of 4-fluoroisobenzofuran-1,3-dione (1.0 g, 6.02 mmol, 1.0 equiv) and 2,6-dioxopiperidine-3-ammonium chloride (0.99 g, 6.02 mmol, 1.0 equiv) in AcOH (50 mL), NaOAc (0.59 g, 7.22 mmol, 1.2 equiv) was added. The resulting reaction mixture was refluxed and stirred for 3 h, followed by pouring into water (300 mL) and extraction with EtOAc (3 × 150 mL). The organic layers were combined, dried with Na_2_SO_4_, and evaporated under reduced pressure. The resulting residue was purified by Büchi flash chromatography (DCM to 3.5% MeOH/DCM) to yield the title compound as a white solid (1.3 g, 4.76 mmol, 78%). ^1^H NMR (300 MHz, DMSO-*d*_6_) δ 11.12 (s, 1H), 7.92 (ddd, *J* = 8.3, 7.3, 4.6 Hz, 1H), 7.80–7.66 (m, 2H), 5.13 (dd, *J* = 13.0, 5.4 Hz, 1H), 2.94–2.77 (m, 1H), 2.63–2.49 (m, 2H), 2.10–1.98 (m, 1H). ^13^C NMR (75 MHz, DMSO-*d*_6_) δ 173.2, 170.2, 166.6, 164.4, 156.8 (d, *J* = 262.2 Hz), 138.1 (d, *J* = 8.0 Hz), 133.9, 123.0 (d, *J* = 19.6 Hz), 120.5, 117.1 (d, *J* = 12.5 Hz), 49.5, 31.4, 22.3.

#### 2-(2,6-Dioxopiperidin-3-yl)-4-((2-(2-(prop-2-yn-1-yloxy)ethoxy)ethyl)amino)isoindoline-1,3-dione (**483a**)

To a solution of compound **482** (200 mg, 0.72 mmol, 1.0 equiv) in DMF (5 mL) were added 2-(2-(prop-2-yn-1-yloxy)ethoxy)ethan-1-amine (134.6 mg, 0.94 mmol, 1.3 equiv) and TEA (195 μL, 1.44 mmol, 2.0 equiv). The reaction mixture was stirred for 24 h at 95 °C. After the reaction was completed, the solvents were evaporated under reduced pressure and the resulting residue was taken up in EtOAc (10 mL). The organic layer was washed with brine (5 mL), followed by drying with Na_2_SO_4_ and evaporating under reduced pressure. The resulting residue was purified by Büchi flash chromatography (heptane to 70% heptane/EtOAc) to yield the title compound as a yellow solid (70 mg, 0.18 mmol, 24%). ^1^H NMR (300 MHz, CDCl_3_) δ 8.34 (s, 1H), 7.41 (dd, *J* = 8.5, 7.1 Hz, 1H), 7.02 (dd, *J* = 7.1, 0.6 Hz, 1H), 6.85 (dd, *J* = 8.5, 0.6 Hz, 1H), 4.91–4.78 (m, 1H), 4.13 (d, *J* = 2.4 Hz, 2H), 3.67–3.54 (m, 6H), 3.41 (t, *J* = 5.5 Hz, 2H), 2.85–2.63 (m, 3H), 2.36 (t, *J* = 2.4 Hz, 1H), 2.12–1.98 (m, 1H). ^13^C NMR (75 MHz, C DCl_3_) δ 171.3, 169.3, 168.5, 167.6, 146.8, 136.0, 132.5, 116.9, 111.7, 110.3, 74.6, 70.5, 69.6, 69.2, 58.5, 48.9, 42.4, 31.4, 22.8.

#### 2-(2,6-Dioxopiperidin-3-yl)-4-((2-(2-(2-(prop-2-yn-1-yloxy)ethoxy)ethoxy)ethyl)amino)isoindoline-1,3-dione (**483b**)

This compound was prepared according to the procedure for compound **483a** using **482** (200 mg, 0.72 mmol, 1.0 equiv) and 2-(2-(2-(prop-2-yn-1-yloxy)ethoxy)ethoxy)ethan-1-amine (176.2 mg, 0.94 mmol, 1.3 equiv) as the starting materials. Purification after Büchi flash chromatography (heptane to 70% heptane/EtOAc) yielded the title compound as a yellow solid (65 mg, 0.14 mol, 19%). ^1^H NMR (300 MHz, CDCl_3_) δ 7.42 (dd, *J* = 8.5, 7.1 Hz, 1H), 7.07–6.99 (m, 1H), 6.86 (d, *J* = 8.4 Hz, 1H), 4.92–4.80 (m, 1H), 4.13 (d, *J* = 2.4 Hz, 2H), 3.64 (d, *J* = 9.3 Hz, 10H), 3.41 (q, *J* = 5.5 Hz, 2H), 2.90–2.56 (m, 3H), 2.36 (t, *J* = 2.4 Hz, 1H), 2.13–1.95 (m, 1H). ^13^C NMR (75 MHz, CDCl_3_) δ 171.0, 169.3, 168.3, 167.6, 146.9, 136.1, 132.5, 116.8, 111.7, 110.3, 74.6, 70.7, 70.7, 70.5, 69.5, 69.1, 58.4, 48.9, 42.4, 31.4, 22.8.

#### PROTAC **JYQ-187**

This compound was prepared according to the procedure of **JYQ-191** using azide **479** (10 mg, 22.7 μmol, 1.0 equiv) and alkyne **483a** (19.7 mg, 27.2 μmol, 1.2 equiv) as starting materials. After the reaction was complete, the reaction mixture was poured into water (20 mL) and extracted with DCM (3 × 20 mL). The combined organic layer was washed with brine, followed by drying with Na_2_SO_4_ and evaporating under reduced pressure. The resulting residue was purified by Büchi flash chromatography (DCM to 6% MeOH/DCM) to yield **JYQ-187** as a yellow solid (8.5 mg, 8.4 μmol, 26%). ^1^H NMR (300 MHz, DMSO-*d*_6_) δ 12.27 (s, 1H), 11.10 (s, 1H), 7.94–7.65 (m, 5H), 7.62–7.55 (m, 1H), 7.13 (d, *J* = 8.6 Hz, 1H), 7.03 (d, *J* = 6.9 Hz, 1H), 6.60 (t, *J* = 5.8 Hz, 1H), 5.41 (s, 2H), 5.05 (dd, *J* = 12.9, 5.4 Hz, 1H), 4.79 (s, 1H), 4.51 (s, 3H), 3.97 (s, 1H), 3.70–3.51 (m, 14H), 3.01 (d, *J* = 22.2 Hz, 4H), 2.90–2.52 (m, 8H), 2.27–1.93 (m, 3H). ^13^C NMR (75 MHz, DMSO-*d*_6_) δ 173.3, 170.9, 170.6, 169.4, 167.8, 164.9, 146.8, 144.1, 137.6, 136.7, 135.7, 132.5, 132.2, 130.6, 129.2, 126.2, 126.0, 118.5, 117.9, 117.6, 111.1, 109.7, 70.1, 69.3, 63.9, 52.5, 51.0, 50.4, 49.0, 46.1, 44.0, 43.5, 42.1, 41.2, 31.4, 29.6, 22.6. HR-MS calculated for C_45_H_49_N_13_O_11_S_2_ [M + H]^+^ 1012.3194, found 1012.3198.

#### PROTAC **JYQ-188**

This compound was prepared according to the procedure for **JYQ-187** using azide **479** (20.0 mg, 32.6 μmol, 1.0 equiv) and alkyne **483b** (17.4 mg, 39.2 μmol, 1.2 equiv) as starting materials. Purification after Büchi flash chromatography (DCM to 6% MeOH/DCM) yielded **JYQ-188** as a yellow solid (10.8 mg, 10.2 μmol, 31%). ^1^H NMR (300 MHz, DMSO-*d*_6_) δ 12.26 (s, 1H), 11.10 (s, 1H), 7.99–7.63 (m, 5H), 7.57 (dd, *J* = 8.6, 7.1 Hz, 1H), 7.12 (d, *J* = 8.6 Hz, 1H), 7.03 (d, *J* = 7.0 Hz, 1H), 6.58 (t, *J* = 5.7 Hz, 1H), 5.40 (s, 2H), 5.04 (dd, *J* = 12.9, 5.4 Hz, 1H), 4.78 (s, 1H), 4.49 (s, 3H), 3.96 (s, 1H), 3.78–3.57 (m, 15H), 3.43–3.25 (m, 5H), 3.01 (d, *J* = 22.2 Hz, 4H), 2.90–2.60 (m, 3H), 2.60–2.54 (m, 1H), 2.31–1.94 (m, 3H). ^13^C NMR (75 MHz, DMSO-*d*_6_) δ 173.4, 171.0, 170.6, 169.4, 167.8, 164.9, 146.9, 144.1, 137.5, 136.8, 135.7, 132.5, 132.1, 117.9, 117.6, 111.2, 109.6, 70.2, 69.3, 63.9, 52.5, 51.0, 50.4, 49.0, 46.1, 45.9, 43.5, 42.1, 31.4, 29.4, 22.6. HR-MS calculated for C_47_H_53_N_13_O_12_S_2_ [M + H]^+^ 1056.3456, found 1056.3456.

#### PROTAC **JYQ-194**

This compound was prepared according to the procedure for **JYQ-187** using azide **481** (20.0 mg, 45.5 μmol, 1.0 equiv) and alkyne **483a** (21.8 mg, 54.5 μmol, 1.2 equiv) as starting materials. Purification after Büchi flash chromatography (DCM to 5% MeOH/DCM) yielded **JYQ-194** as a yellow solid (8.1 mg, 9.7 μmol, 21%). ^1^H NMR (300 MHz, DMSO-*d*_6_) δ 12.25 (d, *J* = 11.8 Hz, 1H), 11.09 (s, 1H), 8.78 (d, *J* = 8.5 Hz, 1H), 8.11–7.92 (m, 2H), 7.63–7.50 (m, 2H), 7.13 (d, *J* = 8.6 Hz, 1H), 7.03–6.98 (m, 1H), 6.59 (t, *J* = 5.7 Hz, 1H), 5.02 (dd, *J* = 12.9, 5.4 Hz, 1H), 4.84 (s, 1H), 4.61 (d, *J* = 2.4 Hz, 2H), 4.00 (t, *J* = 5.9 Hz, 1H), 3.61 (tt, *J* = 7.6, 4.3 Hz, 10H), 3.44–3.24 (m, 6H), 2.94–2.72 (m, 2H), 2.71–2.53 (m, 2H), 2.29–2.00 (m, 3H). ^13^C NMR (75 MHz, DMSO-*d*_6_) δ 173.4, 171.0, 170.6, 169.4, 167.8, 163.9, 157.5 (d, *J* = 247.7 Hz), 156.7, 146.8, 145.7, 143.5, 143.1, 136.7, 133.8, 132.5, 125.7 (d, *J* = 20.7 Hz), 123.6, 122.9, 120.7 (d, *J* = 11.3 Hz), 118.3, 117.9, 117.6, 111.2, 109.6, 70.1, 69.6, 69.4, 63.8, 52.5, 50.4, 49.0, 44.8, 43.5, 42.1, 31.4, 29.7, 22.6. HR-MS calculated for C_39_H_38_FN_11_O_8_S [M + H]^+^ 840.2688, found 840.2691.

#### PROTAC **JYQ-195**

This compound was prepared according to the procedure for **JYQ-187** using azide **481** (20.0 mg, 45.5 μmol, 1.0 equiv) and alkyne **483b** (24.2 mg, 54.5 μmol, 1.2 equiv) as starting materials. Purification after Büchi flash chromatography (DCM to 5% MeOH/DCM) yielded **JYQ-195** as a yellow solid (6.5 mg, 7.4 μmol, 16%). ^1^H NMR (300 MHz, DMSO-*d*_6_) δ 12.25 (d, *J* = 12.4 Hz, 1H), 11.10 (s, 1H), 8.80 (s, 1H), 8.11–7.93 (m, 2H), 7.65–7.52 (m, 2H), 7.11 (d, *J* = 8.6 Hz, 1H), 7.02 (d, *J* = 6.9 Hz, 1H), 6.57 (t, *J* = 5.8 Hz, 1H), 5.03 (dd, *J* = 12.8, 5.4 Hz, 1H), 4.84 (s, 1H), 4.60 (d, *J* = 2.4 Hz, 2H), 4.53 (s, 1H), 4.00 (t, *J* = 5.8 Hz, 1H), 3.71–3.56 (m, 15H), 3.46–3.25 (m, 6H), 2.96–2.54 (m, 2H), 2.30–2.10 (m, 1H), 2.10–1.91 (m, 2H). ^13^C NMR (75 MHz, DMSO-*d*_6_) δ 173.4, 171.0, 170.6, 169.4, 167.8, 163.9, 163.7, 157.1 (d, *J* = 248.1 Hz), 156.8, 146.8, 145.8, 143.6, 143.1, 136.7, 133.8, 132.5, 125.7 (d, *J* = 20.7 Hz), 123.6, 118.3, 117.9, 117.6, 111.2, 109.6, 70.2, 69.6, 69.3, 63.8, 52.5, 50.3, 49.0, 44.8, 43.5, 42.1, 31.4, 29. 7, 27.2, 26.4, 22.6. HR-MS calculated for C_41_H_42_FN_11_O_9_S [M + H]^+^ 884.2950, found 884.2953.

### Echo-Mediated High-Throughput Synthesis

Stock solutions of the cyanimide amine in DMSO (200 mM), carboxylic acids in DMSO (100 mM), and a mixture of HOBt and DIC in DMSO (111.1 mM) were prepared. The stock solutions of the amine (125 nL, final concentration 10 mM) and carboxylic acids (1250 nL, final concentration 50 mM) were transferred to an Echo-ready 1536-well LDV plate (Labcyte LP-0400) by using a Labcyte Echo550 acoustic dispenser, followed by adding the mixture of HOBt and DIC (1125 nL, final concentration 10 mM). The plate was sealed and kept overnight at room temperature. In total, 471 compounds were synthesized as crude reaction mixtures in a total volume of 2.5 μL per well with a maximum concentration of 10 mM, assuming 100% reaction conversion. LC-MS analysis of a representative number of compounds (38 in total) was used to confirm compound formation and purity (Supporting Information Figure S11). A 1 mM daughter plate was made by diluting the compounds 10x in DMSO (300 nL compound solution plus 2700 nL DMSO) in an Echo-ready 1536-well LDV plate (Labcyte LP-0400) by using a Labcyte Echo550 acoustic dispenser.

### High-Throughput Screening

The screen was performed in a buffer containing 50 mM Tris-HCl, 150 mM NaCl, 2 mM TCEP, pH 7.5, 1 mg/mL CHAPS, and conducted in a 1536-well plate (Corning 3724) with a reaction volume of 8 μL per well. Stock solutions of 0.26 μM PARK7 and 80 nM **JYQ-107** were prepared. Using a Labcyte Echo550 acoustic dispenser, 10 nL of 1 mM DMSO stock solutions of the library compounds was transferred from the source plates into the empty 1536-well screening plates to obtain a 1.25 μM final compound concentration. Next, PARK7 (6 μL, final concentration 0.2 μM) was dispensed using a Biotek MultiflowFX liquid dispenser and incubated for 2 h, followed by dispensing the probe **JYQ-107** (2 μL, final concentration 20 nM). After 2 h incubation, the FP signal was recorded on a BMG Labtech PHERAstar plate reader (λex/em 480/520 nm). The percentage inhibition of each compound was calculated from the FP values, normalized to the positive (5 mM *N*-ethylmaleimide, 100% inhibition) and negative (DMSO, 0% inhibition) controls.

### Hit Picking and Validation

The validation was performed using the conditions described above in a 384-well plate (Corning 3820) with a reaction volume of 20 μL per well in triplicate. Fifty four compounds showing inhibition above 90% were picked and checked at three different concentrations. Using a Labcyte Echo550 acoustic dispenser, 20, 10, and 5 nL of 1 mM compound stock solutions were transferred from the source plates into an empty 384-well plate to obtain 1, 0.5, 0.25 μM final concentrations. Next, PARK7 (15 μL, final concentration 0.2 μM) was dispensed with a Biotek MultiFlowFX liquid dispenser and incubated for 1 h, followed by dispensing the probe **JYQ-107** (5 μL, final concentration 20 nM). The FP signal was monitored on a BMG Labtech PHERAstar plate reader (λex/em 480/520 nm) for 2 h. The relative loss of FP signal compared with reference controls (5 mM NEM, 100% inhibition, and DMSO, 0% inhibition) was used to calculate the remaining enzyme activity. The inhibition percentage of these 54 compounds was plotted as heatmaps using Graphpad Prism 9.0.1 software.

### Cell Lines and Cell culture

HEK293T and HeLa cells were originally obtained from the American Type Culture Collection (ATCC). A549 cells and MCF7 cells were kindly provided by Prof. Dr. Peter ten Dijke (LUMC), while H1299 cells were kindly provided by Dr. A.G. Jochemsen (LUMC). All cell lines were cultured in Dulbecco’s modified Eagles’ medium (DMEM) (Gibco) supplemented with 7.5% fetal bovine serum (FBS) at 37 °C and 5% CO_2_.

### Cell Viability Assay

5 × 10^3^ A549 cells were seeded into 96-well plates. The following day, the indicated final concentrations of **JYQ-164** and **JYQ-173** were added to the cells, and DMSO was used as a control. Inhibitors were renewed at 36 h, and cells were incubated with them an additional 36 h. Cell viability was measured using the CellTiter-Blue viability assay (Promega). Relative survival was normalized to the DMSO-treated sample and corrected for background signal.

### siRNA Transfection

Oligos used to knockdown PARK7 were purchased from Dharmacon (Cat#: MQ-005984-00-0002). Silencing was performed in HEK293T and A549 cells as follows: for a 24-well plate format, 50 μL of siRNA (500 nM stock) was incubated with 1 μL of Dharmafect reagent 1 (Dharmacon) diluted in 50 μL of medium without supplements (total volume of 100 μL of transfection mix) with gentle shaking for 20 min at room temperature (RT). A total of 30 × 10^3^ HEK293T or 50 × 10^3^ cells were added to transfection mixes to a total volume of 500 μL per well and cultured for 48 h prior to incubation with 5 μM final concentration of **JYQ-196** for 4 h.

### Antibodies and Fluorescent Dyes

The following antibodies were used for the detection of endogenous protein by immunoblot analysis in a 1:1000 dilution: rabbit anti-PARK7 (Abcam, Cat# ab18257), rabbit anti-UCHL1 (Abcam, Cat# ab27053), Mouse anti-β-actin (Sigma-Aldrich, Cat# A5441) was used as a loading control in a 1:10,000 dilution for Western blot. Secondary IRDye 800CW goat antirabbit IgG (H + L) (Li-COR, Cat# 926-32211, 1:5000) and IRDye 680LT goat antimouse IgG (H + L) (Li-COR, Cat# 926-68020, 1:20,000) were used for detection using the Odyssey Classic imager (LI-COR).

### SDS-PAGE and Immunoblotting

Samples were separated by 12 or 4–12% SDS-PAGE. After proteins were transferred to a nitrocellulose membrane at 300 mA for 2.5 h, the membranes were blocked with 5% skim milk in PBS and incubated with a primary antibody diluted in 5% skim milk in 0.1% PBS-Tween 20 (PBST) for 1 h at RT. After washing with 0.1% PBST three times for 10 min, proteins were incubated with secondary antibodies diluted in 0.1% PBST for 30 min and washed three times again in 0.1% PBST. The signal was detected using direct imaging by the Odyssey Classic imager (LI-COR).

### Cell Lysate Preparation

Cell pellets were suspended in the cell lysis buffer (50 mM Tris, 150 mM NaCl, 0.5% Triton X-100, and 2 mM TCEP at pH 7.5) supplemented with protease inhibitor cocktail (11836145001, Roche). The samples were kept on ice and sonicated using 10 cycles of 30 s pulse on, 30 s pulse off (Bioruptor, Diagenode). The cell lysate was centrifuged at 14,000 rpm with Eppendorf Centrifuge 5430 R for 20 min at 4 °C, and supernatant fractions were collected.

### Cell-Based Competition Assay

HEK293T cells were treated with the indicated concentrations of indicated inhibitors for indicated incubation time. The cells were then washed with PBS and collected, followed by cell lysis. The prepared cell lysate of each sample was incubated by **JYQ-92** (1 μM) for 30 min, followed by adding NuPAGE LDS sample buffer (4X). Samples were resolved by SDS-PAGE using a 12% Bis-Tris gel with MES SDS running buffer and visualized by fluorescence scanning on a Typhoon FLA 9500 using a Cy5 channel (λ_ex/em_ 635/655 nm), followed by protein transfer to nitrocellulose membranes and immunoblot analysis.

### IC_50_ Determination

The assay was performed in PBS buffer and conducted in a 384-well plate (Corning 3820) with a reaction volume of 20 μL in triplicate. Stock solutions of compounds of 0.01, 0.1, and 1 mM were prepared. Compounds were transferred using a Labcyte Echo550 acoustic dispenser to obtain a 12-point serial dilution of 0.001–100 μM. Next, PARK7 (15 μL, final concentration 0.1 μM) was dispensed with a Biotek MultiFlowFX liquid dispenser and incubated for 1 h, followed by dispensing the substrate DiFMUAc (5 μL, final concentration 300 μM). The fluorescence intensity (FI) signal was monitored on a BMG Labtech PHERAstar plate reader (λ_ex/em_ 350/450 nm) for 1 h. All samples were normalized to the positive and negative controls and plotted against the inhibitor concentrations (in μM) using the built-in equation “[inhibitor] vs response–variable slope (four parameters), least-squares fit” with constraints “Bottom = 0” and “Top = 100” in GraphPad Prism 9.0.1 software to obtain the IC_50_ values.

### Covalent Complex Formation Mass Spectrometry Analysis

The stock concentration of 20 μM PARK7 was prepared in Tris-buffer (50 mM Tris-HCl, 150 mM NaCl, 2 mM TCEP, pH 7.5), followed by incubating with DMSO or 30 μM indicated compounds in buffer (10 μM PARK7 and 15 μM indicated compounds final concentration) for 1 h. Samples were then diluted 10-fold with acetonitrile:water (1:1), and 1 μL of sample was injected to LC-MS analysis. LC-MS analysis was performed on Waters Acquity H-class UPLC with a UPLC protein BEH C4 column (1.7 μm, 2.1 × 50 mm) coupled to a Xevo G2-XS Qtof mass spectrometer with ESI. Samples were run using CH_3_CN/H_2_O gradients equipped with 0.1% formic acid for 7 min (2–100% CH_3_CN). Deconvoluted mass was obtained from convolution spectra (50–1200 *m*/*z*) using with MaxEnt1 function of Waters MassLynx mass spectrometry software 4.1.

### Probe Labeling of Purified Recombinant PARK7

The assay was conducted in Tris-buffer (50 mM Tris-HCl, 150 mM NaCl, 2 mM TCEP, pH 7.5). PARK7 (1 μM final concentration) was incubated with different concentrations (0, 0.1, 0.5, 1, 2, 5, and 10 μM) of the indicated probes for 1 h at 37 °C. After completing the incubation time, all of the reactions were stopped by adding NuPAGE LDS sample buffer (4x). These samples were resolved by SDS-PAGE using precast 12% Bis-Tris gels (Invitrogen, NuPAGE) with MES SDS running buffer (Novex, NuPAGE). The gels were visualized by Typhoon FLA 9500 (GE Healthcare Life Sciences) fluorescence scanning: PARK7-**JYQ-191** and PARK7-**JYQ-196** adducts were visualized with a Rhodamine channel (λ_ex/em_ 473/530 nm); PARK7-**JYQ-192** and PARK7-**JYQ-197** adducts were visualized with a Cy5 channel (λ_ex/em_ 635/655 nm), followed by staining with Instant Blue Coomassie protein stain (Expedeon) and scanning on an Amersham Imager 600 (GE Healthcare Life Sciences).

### Probe Labeling of Endogenous PARK7 in Cells

HEK293T or A549 cells were incubated with indicated concentration of the probes for the indicated time points, followed by cell lysis and adding loading buffer (4x). Samples were resolved by SDS-PAGE using a 12% Bis-Tris gel with MES SDS running buffer and visualized by fluorescence scanning on a Typhoon FLA 9500 using a Cy5 channel (λ_ex/em_ 635/655 nm), followed by transferring to nitrocellulose membranes and immunoblot analysis.

### Confocal Microscopy

A549 cells were seeded into 24-well plates (Costar, Cat# 3524) containing glass coverslips (Menzel Gläser, Cat# MENZCB00130RAC) and incubated with 5 μM of final concentration of probes **JYQ-192**, **JYQ-196**, and **JYQ-197** for fluorescence confocal microscopy of fixed samples. Fixation was performed in 3.7% formaldehyde (acid-free, Merck Millipore) in phosphate-buffered saline (PBS) for 20 min. After washing 3× with PBS, cells were mounted using a ProLong Gold antifade Mounting medium with DAPI (Life Technologies, Cat# P36941). Samples were imaged using a Leica SP8 microscope equipped with appropriate solid-state lasers, HCX PL 63 times magnification oil emersion objectives and HyD detectors. Data were collected using a digital zoom in 1 in 1024 by 1024 scanning format with line averaging. Postcollection image processing was performed using the Fiji software.

### Activity Evaluation of PROTACs in Cells

A549 cells were seeded into 6-well plates. The next day after seeding, cells were incubated with the indicated concentrations of indicated PROTACs for 8 h. The cells were then washed with PBS and collected. The collected cell pellets were lysed with Tris-triton buffer as described above. The prepared cell lysate of each sample (10 μL) was added 5 μL 3× LDS + 5% β-mercaptoethanol, and boiled for 10 min. Samples were resolved by SDS-PAGE using a 4–12% Bis-Tris gel with MOPS SDS running buffer, followed by transferring to nitrocellulose membranes and immunoblot analysis.

### Streamlined Cysteine Activity-Based Protein Profiling

A549 cells were seeded into 10 cm plates in 10 mL of media. The day after seeding, cells were incubated with the indicated concentrations of **JYQ-164**, and **JYQ-173** for 4 h. The cells were then washed two times with cold PBS (1 mL per 10 cm plate) and collected by scraping them with a cell scraper. Cells were lysed by sonication in ice-cold PBS and centrifuged at 15,000 rpm for 2 min to remove cell debris. The BCA Gold protein assay (Thermo Fisher Scientific) was performed to determine protein concentration. Each sample (100 μg) was labeled with 500 μM DBIA probe for 1 h in the dark at room temperature (RT). Excess DBIA and disulfide bonds were quenched and reduced, respectively, using 5 mM dithiothreitol (DTT) for 30 min in the dark at RT. Reduced disulfide bonds were alkylated using 20 mM iodoacetamide for 30 min in the dark at RT. Proteins were precipitated using chloroform/methanol, resolubilized in 40 mM HEPES (pH 8.4), and digested using TPCK-treated trypsin and endoGluC (1:12.5 enzyme/protein ratio) overnight at 37 °C. Digested peptides were labeled with TMTpro16-plex reagents (Thermo Fisher Scientific) in a 1:4 ratio by mass (peptides/TMT reagents) for 1 h at RT. Excess TMT reagent was quenched with 5 μL 6% hydroxylamine for 15 min at RT. All samples were then pooled and lyophilized.

Lyophilized samples were reconstituted in 1 mL of PBS and enriched with Pierce streptavidin magnetic beads (Catalog number: 88816, Thermo Scientific) by rotating end-over-end for 4 h at RT. Nonspecific binding peptides were washed away using the following procedure: 3 × 1 mL of PBS pH 7.4, 2 × 1 mL of PBS with 0.1% SDS pH 7.4, and 3 × 1 mL of HPLC-grade water. DBIA probe-containing peptides were eluted using 700 μL of 50% acetonitrile in 0.1% TFA, lyophilized using a Speedvac, and desalted using a handmade mini-SPE column (10% sorbent material of a Waters, OASIS 1 cm^3^ HLB 30 mg cartridge). Column was equilibrated with 200 μL of 90% acetonitrile and 3 × 200 μL of 10 mM NH_4_HCO_3_ pH 8.4. The dried sample was dissolved in 200 μL of 10 mM NH_4_HCO_3_ pH 8.4, loaded onto the column, washed 3x with 200 μL of 10 mM NH_4_HCO_3_ pH 8.4, and eluted into 3 fractions with 150 μL of 10, 20, and 50% acetonitrile (in 10 mM NH_4_HCO_3_). Samples were then lyophilized.

### TMT-Based Global Proteomic Profiling

A549 cells were seeded into 10 cm plates in 10 mL of media. Two days after seeding, cells were incubated with 5 μM **JYQ-173** or **JYQ-194** for 8 h. The cells were then washed two times with cold PBS (1 mL per 10 cm plate) and collected by scraping them with a cell scraper. Cells were lysed using a 5% SDS lysis buffer (100 mM Tris-HCl, pH 7.6) and were incubated at 95 °C for 4 min. Protein determination was performed using the Pierce BCA Gold protein assay. 100 μg of protein of each sample was reduced and alkylated, and excess iodoacetamide was quenched using 5 mM TCEP, 15 mM iodoacetamide, and 10 mM DTT, respectively. The protein was pelleted down using chloroform/methanol precipitation, and the resulting pellets were resolubilized in 40 mM HEPES pH 8.4. Trypsin (1:12.5 enzyme/protein ratio) was added to digest protein overnight at 37 °C. The Pierce BCA Gold protein assay was used to determine the peptide concentration. The peptides were labeled with TMTpro Label Reagents in a 1:4 ratio by mass (peptides/TMT reagents) for 1 h at RT. Five μL of 6% hydroxylamine was added to quench excess TMT reagent and incubated for 15 min at RT. Samples were pooled and lyophilized. The sample was subsequently fractionated on an Agilent 1200 series HPLC system (Agilent Technologies). 75 μg of the pooled sample was dissolved in solvent A (10 mM NH_4_HCO_3,_ pH 8.4), injected onto, and eluted from an Agilent Eclipse Plus C18 2.1 × 150 mm 3.5 μm column (Agilent Technologies). The gradient was run from 2 to 90% solvent B (10 mM NH_4_HCO_3_, pH 8.4 final concentration, 20/80 water/acetonitrile v/v) in 30 min at a flow rate of 200 μL/min. Twelve fractions were made; every 30 s a fraction was collected in a vial, and the eluate of the next 30 s was collected in the next vial and so on until it loops back to the first vial (5 loops of 6 min each). Afterward, the samples were lyophilized.

### Mass Spectrometry

Lyophilized peptides were dissolved in 0.1% formic acid (FA) and analyzed by online C18 nano-HPLC MS/MS with a system consisting of an Ultimate3000nano gradient HPLC system (Thermo, Bremen, Germany), and an Exploris480 mass spectrometer (Thermo). Fractions were injected onto a cartridge precolumn (300 μm × 5 mm, C18 PepMap, 5 μm, 100 A) and eluted via a homemade analytical nano-HPLC column (50 cm × 75 μm; Reprosil-Pur C18-AQ 1.9 um, 120 A (Dr. Maisch, Ammerbuch, Germany)). The gradient was run from 2 to 36% solvent B (20/80/0.1 water/acetonitrile/FA v/v) in 120 min. The nano-HPLC column was drawn to a tip of ∼10 μm and acted as the electrospray needle of the MS source. The mass spectrometer was operated in data-dependent MS/MS mode for a cycle time of 3 s, with a HCD collision energy at 36% and recording of the MS2 spectrum in the Orbitrap, with a quadrupole isolation width of 1.2 Da. In the master scan (MS1), the resolution was 120,000, the scan range 350–1600, at standard AGC target @maximum fill time of 50 ms. A lock mass correction on the background ion *m*/*z* = 445.12 was used. Precursors were dynamically excluded after *n* = 1 with an exclusion duration of 45 s, and with a precursor range of 20 ppm. Charge states 2–5 were included. For MS2, the first mass was set to 110 Da, and the MS2 scan resolution was 45,000 at an AGC target of 200% with a maximum fill time set to auto.

### Data Analysis

For SLC-ABBP, in a postanalysis process, raw data were first converted to peak lists using Proteome Discoverer version 2.4 (Thermo Electron) and then submitted to the Uniprot *Homo sapiens* minimal database (20296 entries), using Mascot v. 2.2.04 (www.matrixscience.com) for protein identification. Mascot searches were done with 10 ppm and 0.02 Da deviation for the precursor and fragment mass, respectively, and the enzymes trypsin and EndoGluC was specified, up to three missed cleavages were allowed. Methionine oxidation, acetyl (protein N-term), and DBIA on Cys were set as a variable modification. Carbamidomethyl on Cys and TMTpro on Lys and N-term were set as a fixed modification. Peptides with an FDR <1% were accepted. Peptides were only retained, if they were quantified and if there was exactly one DBIA modification on the peptide. The values for each TMT channel were normalized to the average of the values for the DMSO samples for the same peptide, and these values were named CR. For each peptide, the first entry of the Master Protein Accessions column was chosen as the Master Protein Accession, and the residue number of the cysteine modified by DBIA within the entire protein sequence was determined. Using this information, each peptide was assigned an identifier consisting of the Master Protein Accession and the residue number of the modified cysteine to assign each peptide to the respective modified cysteine residue. Within each replicate, CR values for all peptides with the same identifier (and thus the same modified cysteine residue) were combined by averaging and only the shortest Annotated Sequence was kept as the final Annotated Sequence. The CR values of all replicates for the same identifier under the same condition were averaged to give the “Average CR” for the respective cysteine under that condition.

For TMT-based global proteomics, in a postanalysis process, raw data were first converted to peak lists using Proteome Discoverer version 2.5 (Thermo Electron) and submitted to the Uniprot database (*Homo sapiens*, 20596 entries), using Mascot v. 2.2.07 (www.matrixscience.com) for protein identification. Mascot searches were with 10 ppm and 0.02 Da deviation for precursor and fragment mass, respectively. Enzyme specificity was set to trypsin. Up to two missed cleavages were allowed. Methionine oxidation and acetyl on protein N-terminus were set as variable modifications. Carbamidomethyl on Cys and TMTpro on Lys and N-terminus were set as fixed modifications. Protein FDR of 1% was set. Normalization was on total peptide amount. The values for each TMT channel were normalized to the average of the values for the DMSO samples for the same peptide, and these values were named FC. The log_2_ FC values of all replicates for the same master protein were averaged to give the “Average log_2_ FC”. A one-sample *t* test was performed against the null hypothesis of log_2_ FC = 0, and the resulting −log_10_*p* values were reported for each protein. The volcano plot was plotted using GraphPad Prism v.9.01.

## Supporting Information Available

Assessment of the cell permeability and covalent bond formation, inhibition of PARK7, activity evaluation of inhibitors, IC_50_ determination, inhibition values and selectivity ratios, cytotoxicity assay, PARK7 labeling, cell permeability and cellular PARK7 labeling efficiency, off-target validation, degradation evaluation, and plate lay-out and LC-MS analysis (PDF)
Supplementary Data S1 including high-throughput screening and validation data (XLSX)
Supplementary Data S2 including SLC-ABPP data analysis of all cysteine sites (XLSX)
Supplementary Data S3 including SLC-ABPP data analysis of unique cysteine sites (XLSX)
Supplementary Data S4 including quantitative TMT-based total proteome profiling data analysis of PROTACs (XLSX)
Molecular formula strings for all compounds (CSV)

## Notes

The mass spectrometry proteomics data have been deposited with the ProteomeXchange Consortium via the PRIDE partner repository with data set identifier PXD047093.

## Abbreviations

ABP: activity-based probe
CHAPS: (3-((3-cholamidopropyl) dimethylammonio)-1-propanesulfonate)
CR: competition ratio
CRBN: cereblon
DBIA: desthiobiotin iodoacetamide
DIC: *N*,*N*′-diisopropylcarbodiimide
DiFMUAc: 6,8-difluoro-4-methylumbelliferyl acetate
DIPEA: *N*,*N*-diisopropylethylamine
DOP1B: DOP1 leucine zipper-like protein B
DUBs: deubiquitinating enzymes
FC: fold change
FP: fluorescence polarization
HCTU: *O*-(1*H*-6-chlorobenzotriazole-1-yl)-1,1,3,3-tetramethyluronium hexafluorophosphate
HINT2: histidine triad nucleotide-binding protein 2
HOBt: 1-hydroxybenzotriazole
LDV: low dead volume
PARK7: Parkinson Disease Protein 7
POI: protein of interest
POMA: pomalidomide
PROTACs: proteolysis targeting chimeras
SLC-ABPP: streamlined cysteine activity-based protein profiling
TCEP: tris(2-carboxyethyl)phosphine
TEA: triethylamine
TM7SF3: transmembrane 7 superfamily member 3
TMT: tandem mass tag
TPCK: tosylsulfonyl phenylalanyl chloromethyl ketone
UCHL1: ubiquitin C-terminal hydrolase L1.
