# Supplementary material for: Development of Inhibitors, Probes, and PROTAC Provides a Complete Toolbox to Study PARK7 in the Living Cell
Source: J Med Chem. 2024 May 7;67(10):7935–53. doi: 10.1021/acs.jmedchem.3c02410 (PMC11129182; doi:10.1021/acs.jmedchem.3c02410)

SUPPORTING INFORMATION

Development of Inhibitors, Probes, and PROTAC Provides a Complete Toolbox to Study PARK7 in the Living Cell

Yuqing Jia,†,‡ Merve Oyken,† Robbert Q. Kim,† Rayman T.N. Tjokrodirijo,§ Arnoud H. de Ru,§ Antonius P. A. Janssen,‖ Stephan M. Hacker,‖ Peter A. van Veelen,§ Paul P. Geurink*,† and Aysegul Sapmaz*,†

† Department of Cell and Chemical Biology, Division of Chemical Biology and Drug Discovery, Leiden University Medical Center, Leiden 2333 ZC, The Netherlands

‡ Laboratory for Organic Chemistry, Department of Chemistry and Applied Biosciences, ETH Zürich, Zürich CH-8093, Switzerland

§ Center for Proteomics and Metabolomics, Leiden University Medical Center, Leiden 2333 ZA, The Netherlands

‖ Department of Molecular Physiology, Leiden Institute of Chemistry, Leiden University, Leiden 2333 CC, The Netherlands.

∗ Corresponding authors: A.S. (a.sapmaz@lumc.nl); P.P.G. (p.p.geurink@lumc.nl)

Table of contents

Figure S1. Assessment of the cell permeability of **JYQ-88** S3

Figure S2. Inhibition of PARK7 by 3 inhibitors and amine precursor S4

Figure S3. Activity evaluation of inhibitors against DUBs in HEK293T cells S5

Figure S4. Assessment of covalent bond formation of **JYQ-164**, **JYQ-173** S6

Figure S5. IC_{50} determination of **JYQ-164**, **JYQ-173** S7

Table S1. Inhibition values and selectivity ratios of **JYQ-88**, **JYQ-164**, and **JYQ-173** between PARK7 and UCHL1 .... S7

Figure S6. Cytotoxicity assay for **JYQ-164** and **JYQ-173** in A549 cells S8

Figure S7. PARK7 labeling in HEK293T and A549 cells with probes S9

Figure S8. Cell permeability and cellular PARK7 labeling efficiency of **JYQ-192**, **JYQ-196**, and **JYQ-197** S10

Figure S9. Off-target validation of **JYQ-196** in HEK293T and A549 cells S12

Figure S10. Degradation evaluation of PROTACs **JYQ-187**, **JYQ-188** S13

Figure S11. Plate lay-out and LC-MS analysis of the compound library S14

NMR spectra S15

LC-MS spectra S44

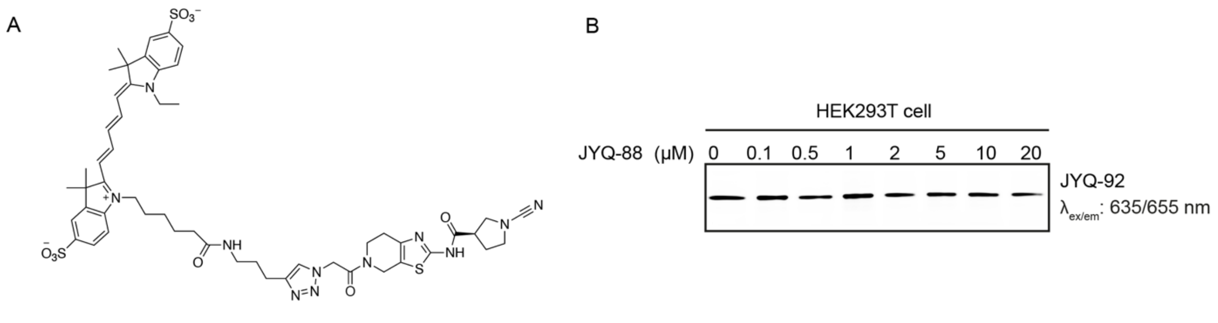

JYQ-92

Figure S1. Assessment of the cell permeability of **JYQ-88**. (A) Structure of **JYQ-92**. (B) Target engagement of **JYQ-88** in HEK293T cells. HEK293T cells were incubated with a concentration series of **JYQ-88** for 24 h, followed by cell lysis and incubation with PARK7 probe **JYQ-92** for 1 h at 37 °C. The reactions were stopped by the addition of NuPAGE™ LDS sample buffer (4x). Samples were resolved by SDS-PAGE using a 12% Bis-Tris gel with MES SDS running buffer (Novex, NuPAGE) and visualized by fluorescence scanning on a Typhoon FLA 9500 (GE Healthcare Life Sciences) using a Cy5 channel ($\lambda_{ex/em}$ 635/655 nm). The disappearance of the **JYQ-92**-labeled band in the cell lysates as a result of HEK293T cells treatment with the indicated concentration of **JYQ-88** is used as a measurement of cellular PARK7 engagement with **JYQ-88**.

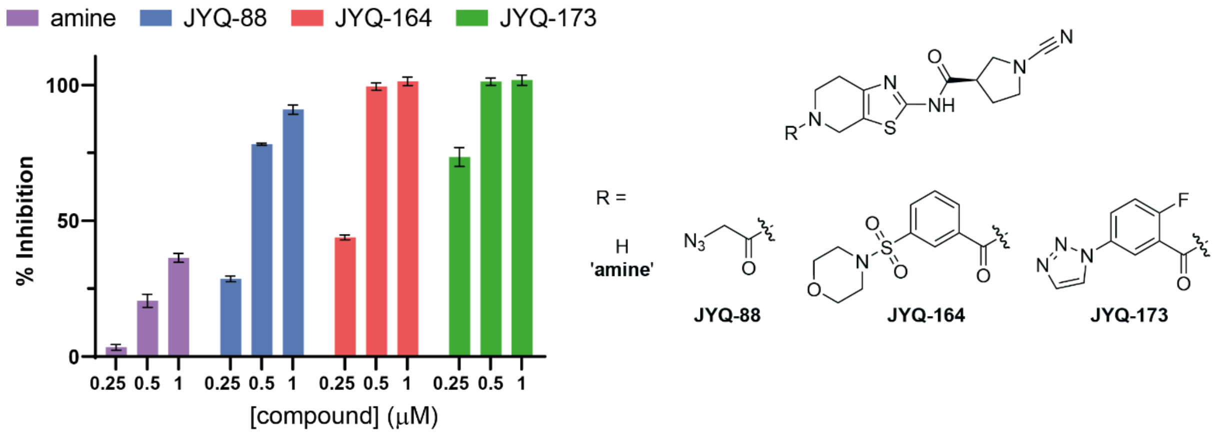

Figure S2. Inhibition of PARK7 at 0.25, 0.5 and 1 μM final concentration of three PARK7 inhibitors and their amine precursor, determined using the PARK7 FP assay.

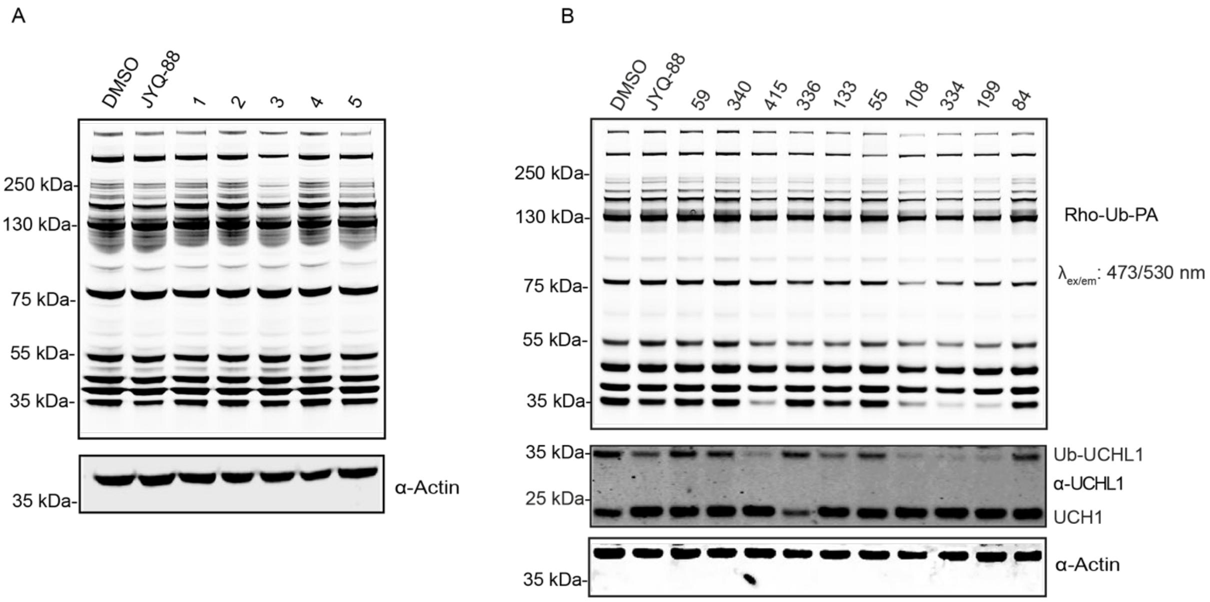

Figure S3. Activity evaluation of inhibitors against DUBs in HEK293T cells. (A) Inhibitors from structure modification. (B) Inhibitors from HTS. HEK293T cells were incubated with 5 μM of the indicated compounds for 24 h, followed by cell lysis and incubation with Rho-Ub-PA probe for 30 min at 37 °C. Samples were resolved by SDS-PAGE using a 4-12% Bis-Tris gel with MOPS SDS running buffer (Novex, NuPAGE) and visualized by fluorescence scanning on a Typhoon FLA 9500 (GE Healthcare Life Sciences) using a Rhodamine channel ($\lambda_{ex/em}$ 473/530 nm), followed by transferring to Nitrocellulose membranes and Western blot analysis.

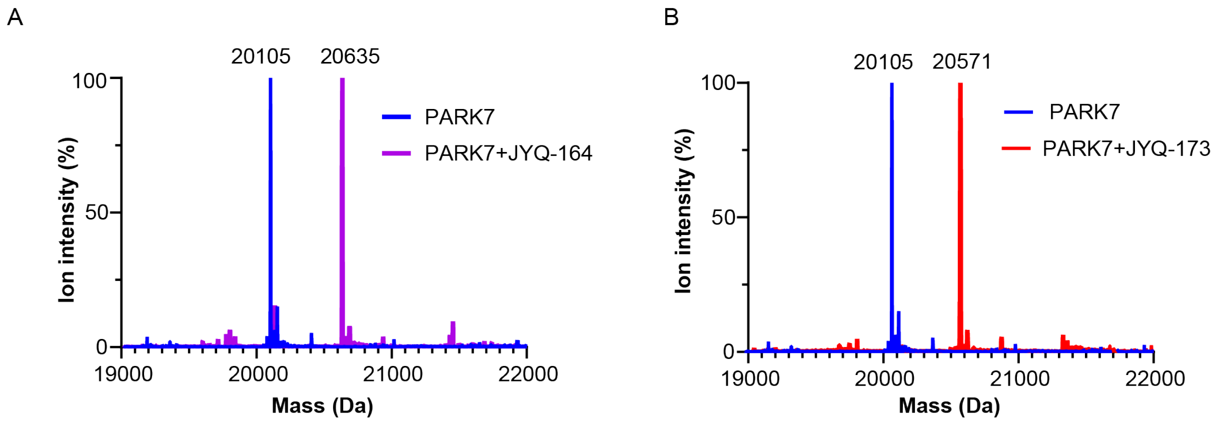

Figure S4. Assessment of covalent bond formation of **JYQ-164** and **JYQ-173** with PARK7. Purified recombinant PARK7 protein was analyzed by intact mass spectrometry following incubation with **JYQ-164** or **JYQ-173**. Both inhibitors formed a covalent complex with PARK7 as indicated by a molecular weight increase of 520 Da (panel A) and 466 Da (panel B).

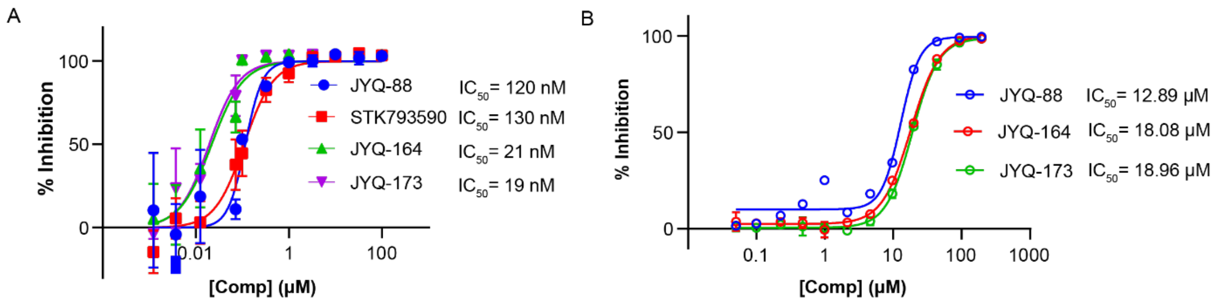

Figure S5. IC_{50} determinations. (A) IC_{50} determination of **JYQ-164**, **JYQ-173**, **JYQ-88**, and **STK793590** for PARK7 using the DiFUMAc assay. (B) IC_{50} determination of **JYQ-88**, **JYQ-164**, and **JYQ-173** for UCHL1 using Ub-RhoMorpholine as substrate.

Table S1. Inhibition values and selectivity ratios of **JYQ-88**, **JYQ-164**, and **JYQ-173** between PARK7 and UCHL1. The inhibition value is given as IC_{50} (μM).

Compound	PARK7	UCHL1	Selectivity ratio
JYQ-88	0.120	12.89	100
JYQ-164	0.021	18.08	861
JYQ-173	0.019	18.96	998

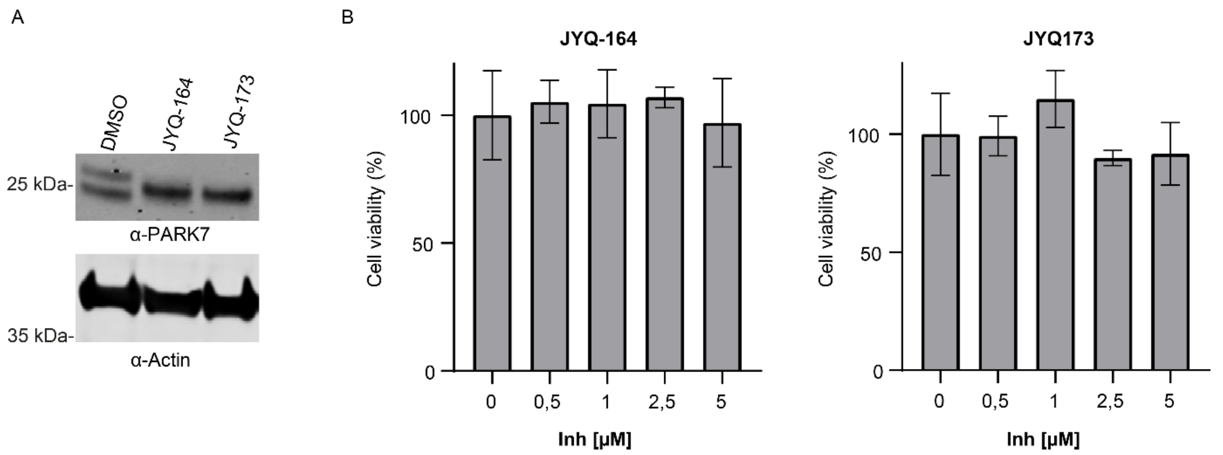

Figure S6. Cytotoxicity assay for **JYQ-164** and **JYQ-173** in A549 cells. (A) PARK7 engagement of **JYQ-164** and **JYQ-173** in A459 cells**.** A549 cells were incubated with 1 µM of the indicated compounds for 72 h, where the inhibitors were renewed after 36 h, followed by cell lysis and incubation with PARK7 probe **JYQ-92** for 1 h at 37 °C. The reactions were stopped by addition of NuPAGE™ LDS sample buffer (4x), followed by cell lysis, SDS-PAGE, fluorescence scanning, and immunoblotting against PARK7 and β-actin. β-actin was used as a loading control. (B) A549 cells were incubated with indicated concentration of **JYQ-164** (left panel) or **JYQ-173** (right panel) for 72 h where the inhibitors were renewed after 36 h. Cell viability was measured using the CellTiter-Blue assay. Relative viability was normalized to untreated control and corrected for background signal.

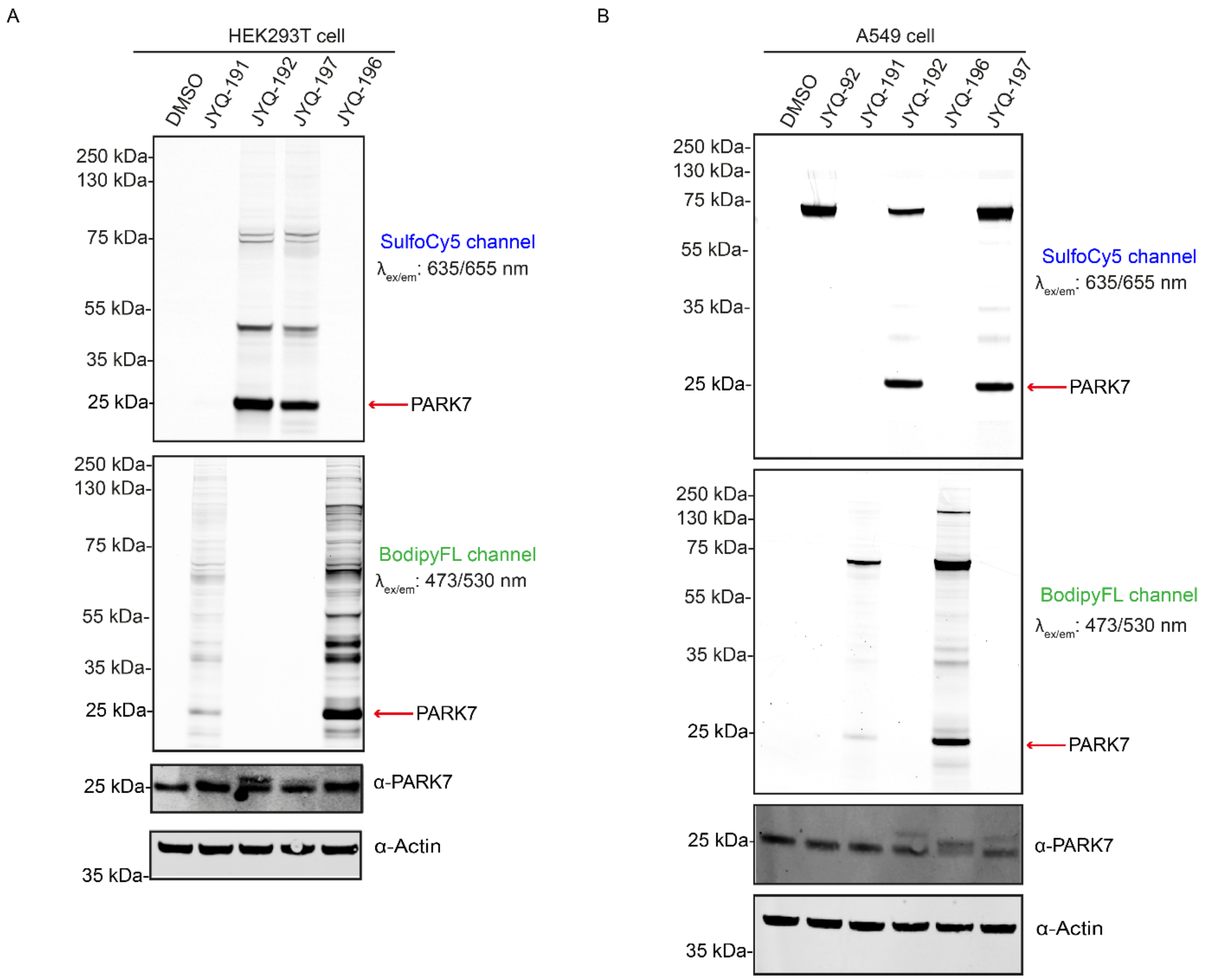

Figure S7. Assessment of PARK7 labeling in HEK293T and A549 cells with probes derived from PARK7 inhibitors **JYQ-164** and **JYQ-173**. HEK293T (A) and A549 (B) cells were incubated with 5 μM final concentration of indicated probes or DMSO for 24 h, followed by cell lysis, SDS-PAGE, fluorescence scanning, and immunoblotting against PARK7 and β-actin. β-actin was used as a loading control.

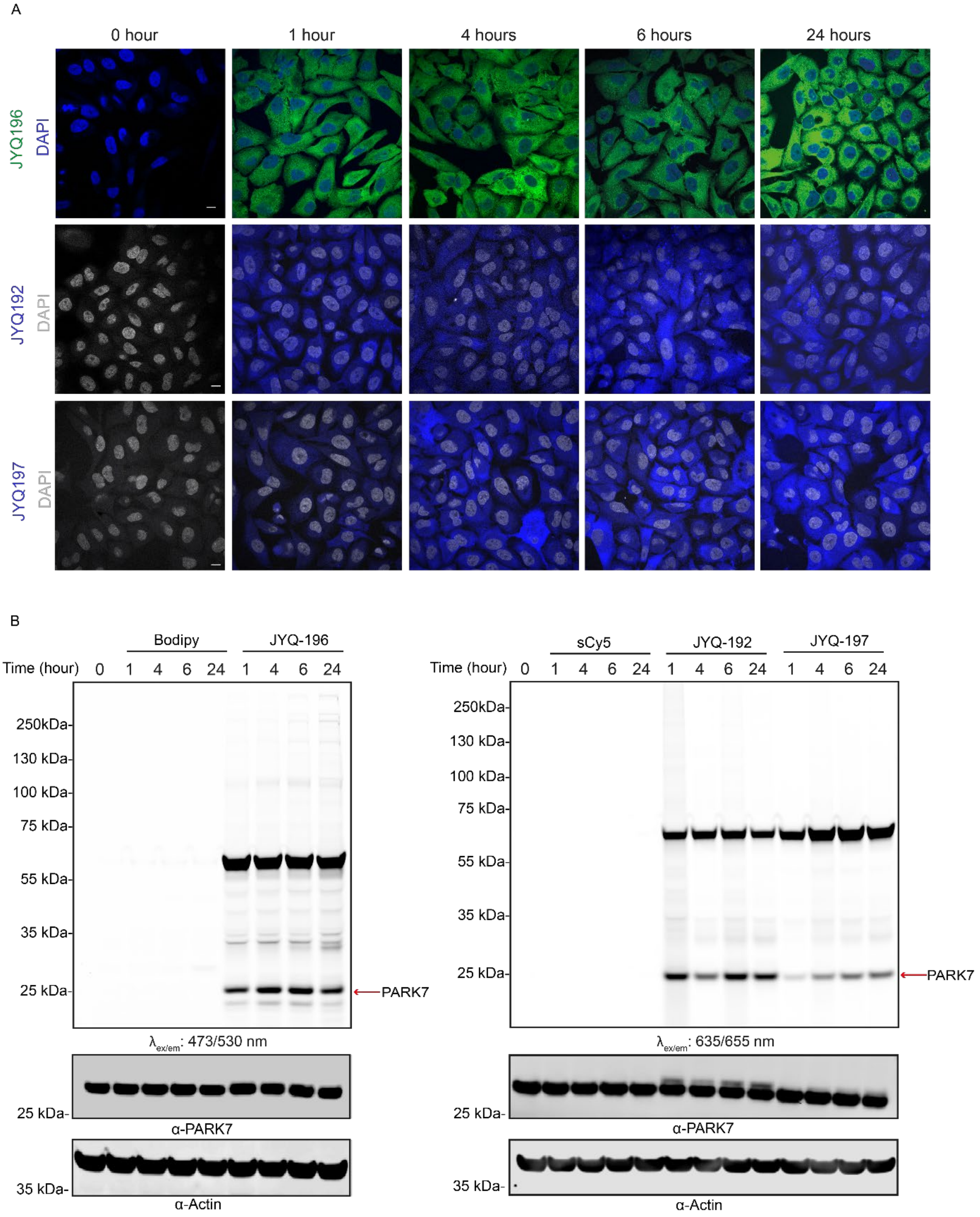

Figure S8. Assessment of cell permeability and cellular PARK7 labeling efficiency of **JYQ-192**, **JYQ-196**, and **JYQ-197**. (A) Representative confocal images of fixed A549 cells treated with 5 μM final concentration of **JYQ-192**, **JYQ-196**, or **JYQ-197** at the indicated time points. **JYQ-196** (green) overlays with DAPI (blue) in the top panel, and **JYQ-192** and **JYQ-197** (blue) overlays with DAPI (gray) in the middle and bottom

panel, respectively. Scale bars = 10 μm. (B) A549 cells treated with 5 μM final concentration of **JYQ-192**, **JYQ-196**, **JYQ-197**, SulfoCy5 or BodipyFL at the indicated time points followed by cell lysis, SDS-PAGE, fluorescence scanning, and immunoblotting against PARK7 and β-actin. β-actin was used as a loading control.

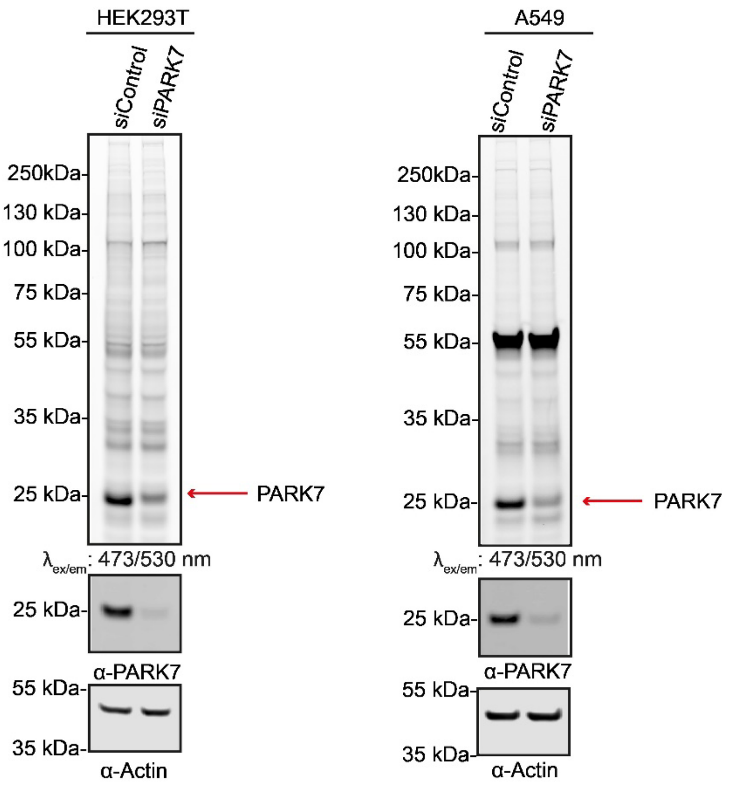

Figure S9. Off-target validation of **JYQ-196** in HEK293T and A549 cells. HEK293T cells and A549 cells were transfected with siControl or siPARK7 and incubated for 48 h. 5 μM final concentration of **JYQ-196** was added to the samples and cells were incubated for 4 h, followed by cell lysis, SDS-PAGE, fluorescence scanning, and immunoblotting against PARK7 and β-actin. β-actin was used as a loading control.

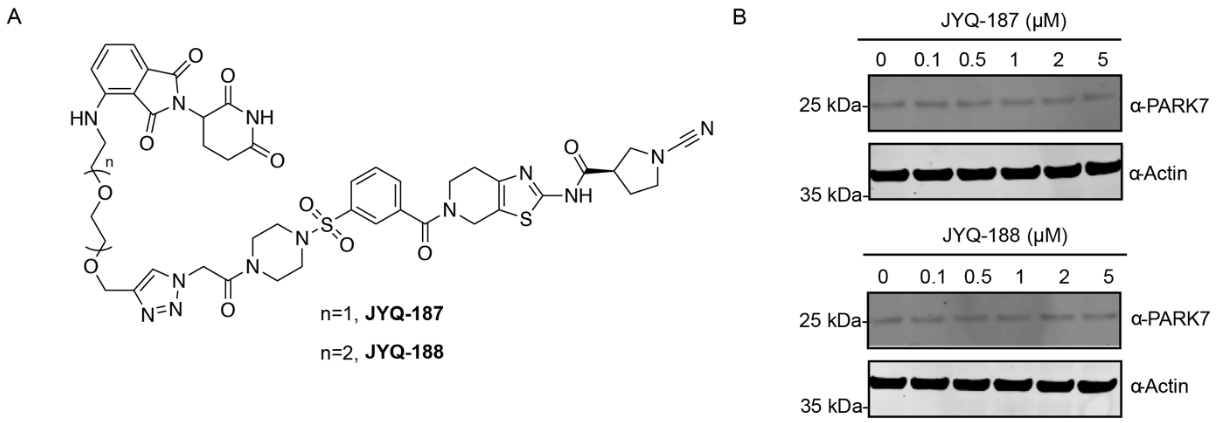

Figure S10. Degradation evaluation of PROTACs **JYQ-187** and **JYQ-188**. (A) Structures of PROTACs **JYQ-187** and **JYQ-188**. (B) PARK7 degradation efficacy with PROTACs **JYQ-187**, **JYQ-188**. A549 cells were incubated with indicated PROTACs for 8 h before cell lysis and western blot analysis.

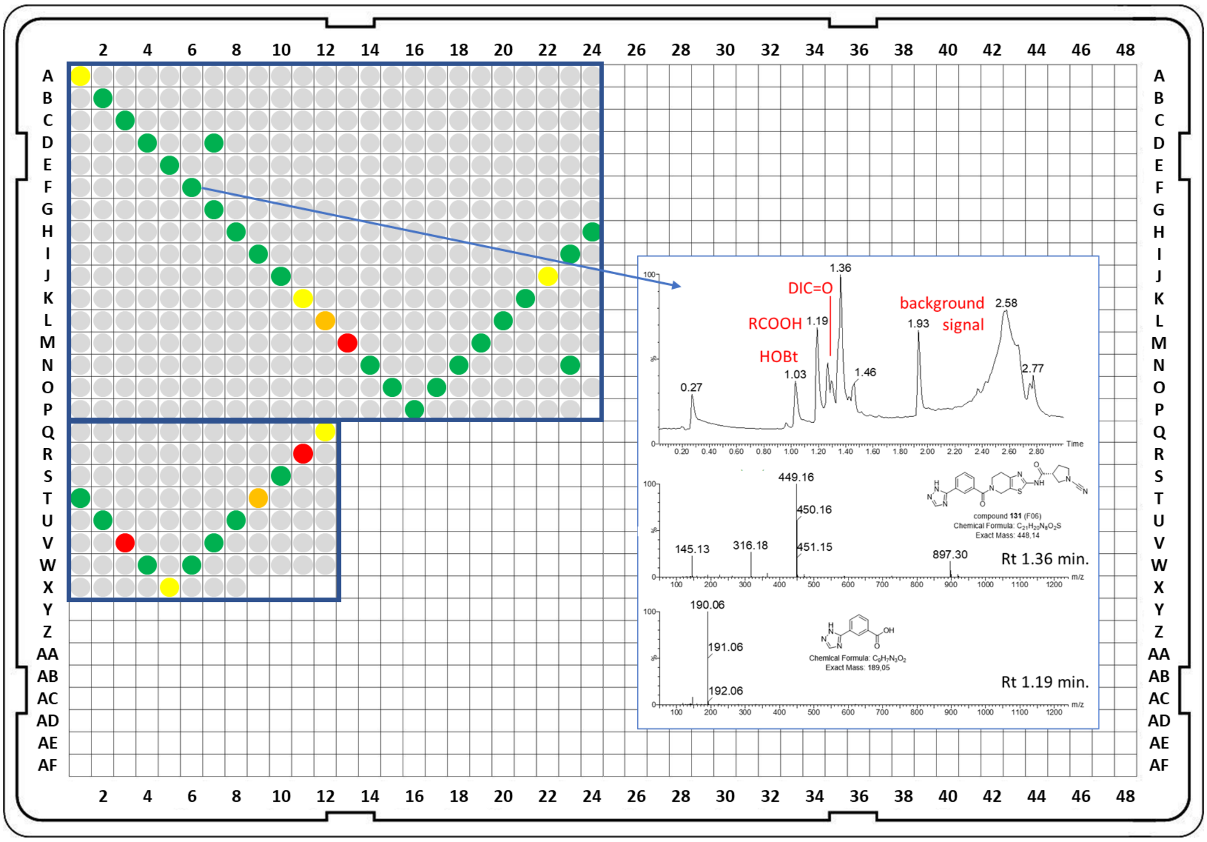

Figure S11. Plate lay-out showing the LC-MS analysis results of the compound library. A representative number of compounds (38 in total) covering all rows and columns in the plate were analyzed as indicated: 30 nL of the crude compound mixtures from the 10 mM synthesis plate was diluted in 30 µL ACN/H_2O 1:1 v/v and analyzed by LC-MS (1 µL injection). Product formation was assessed qualitatively from the LC-MS spectra. Colors represent product quality. Green: main product peak; yellow: intermediate product peak; orange: minor product peak; red: no product observed; grey: not analyzed. Insert: example of a typical LC-MS trace (compound **131**; well F06).

NMR Spectra

^{1}H-NMR and ^{13}C-NMR of compound 1 in DMSO.

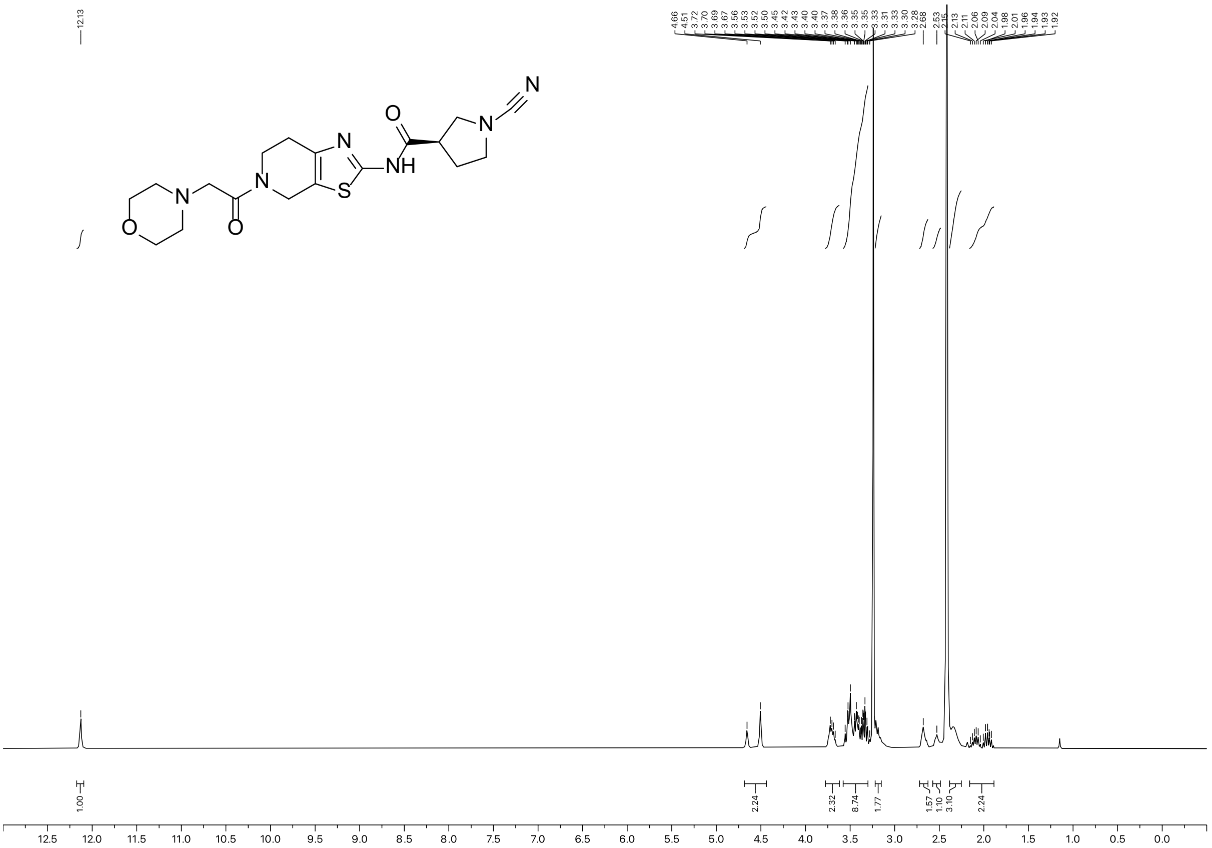

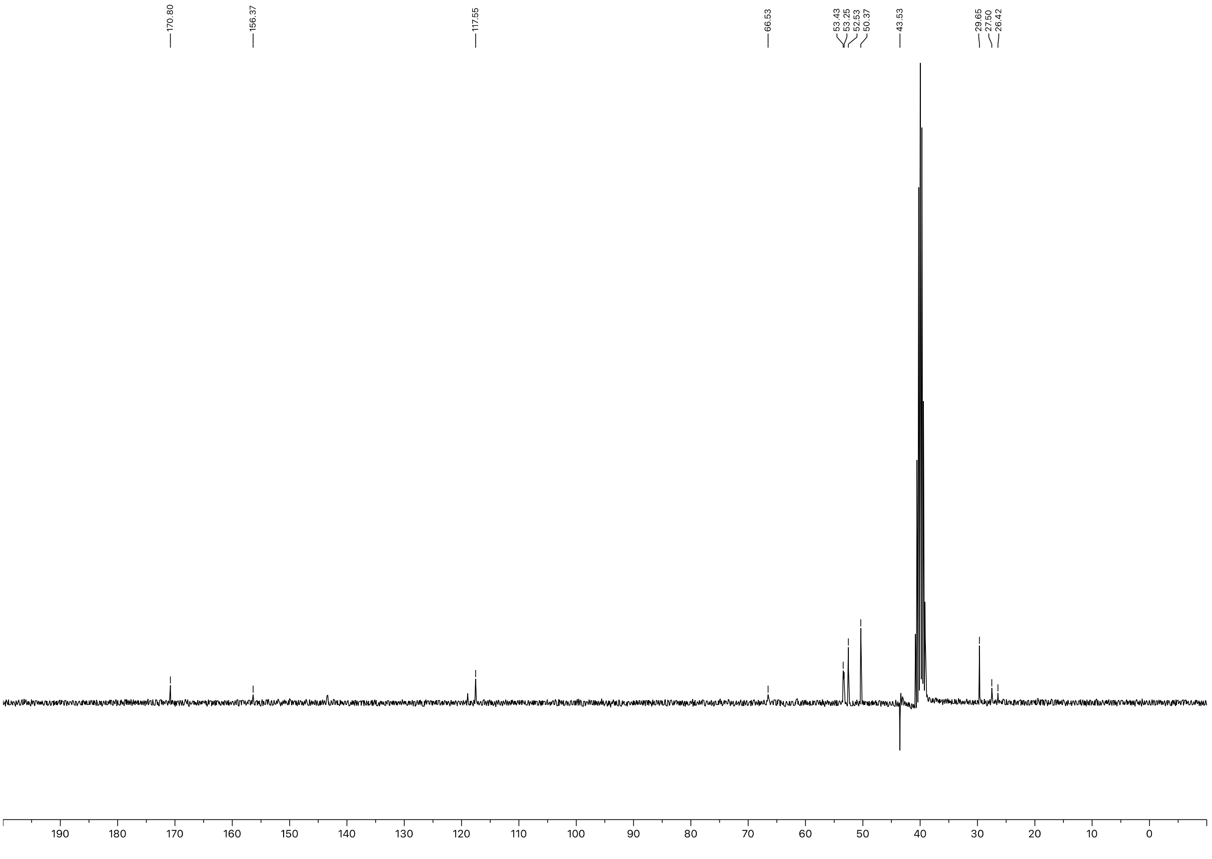

^{1}H-NMR and ^{13}C-NMR of compound 2 in DMSO.

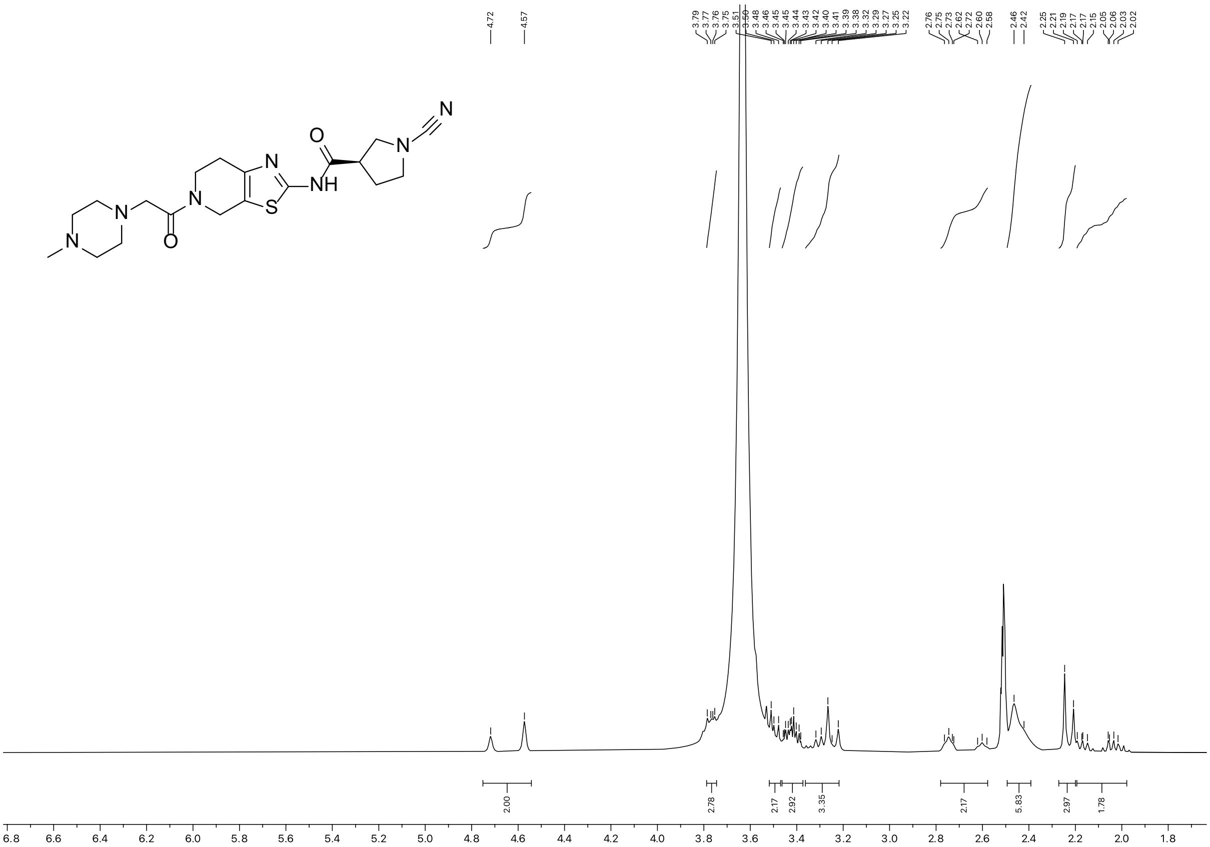

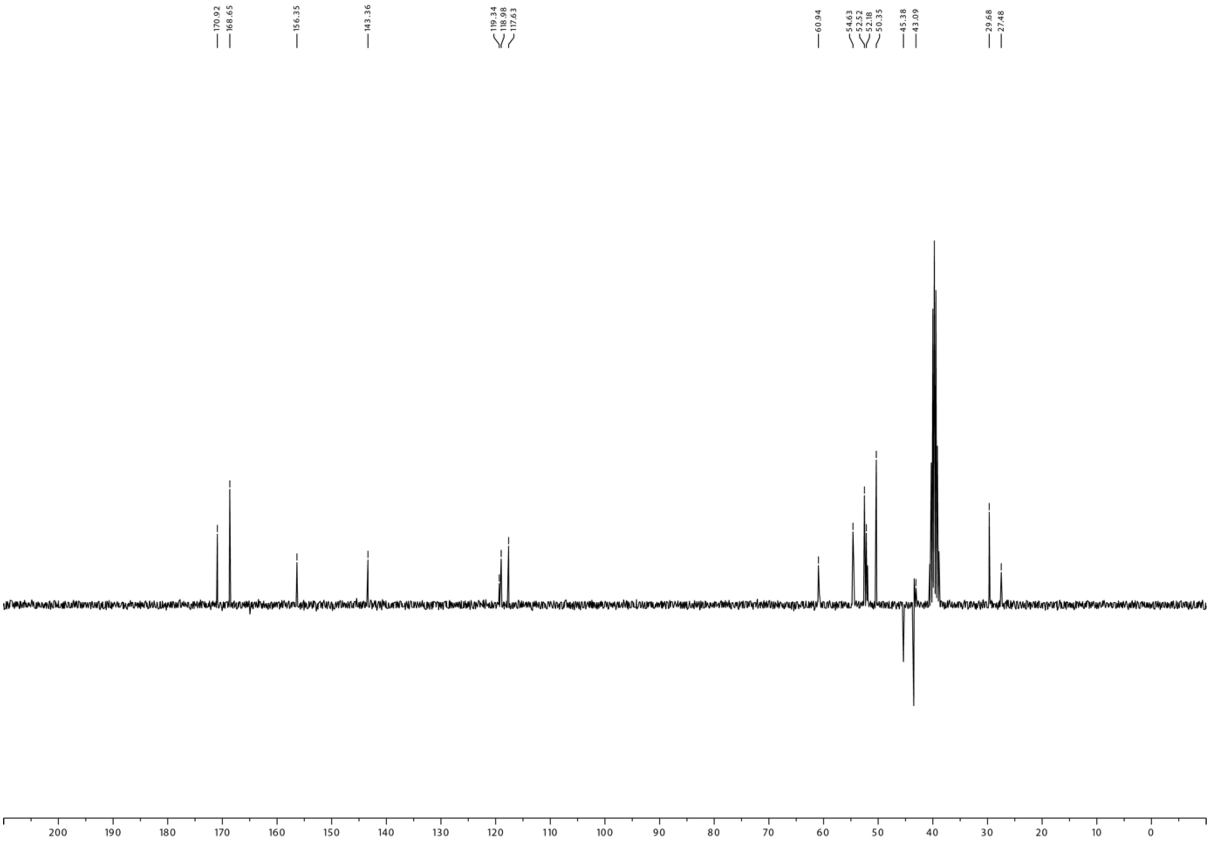

[1] H-NMR and ^{13}C-NMR of compound 3 in DMSO.

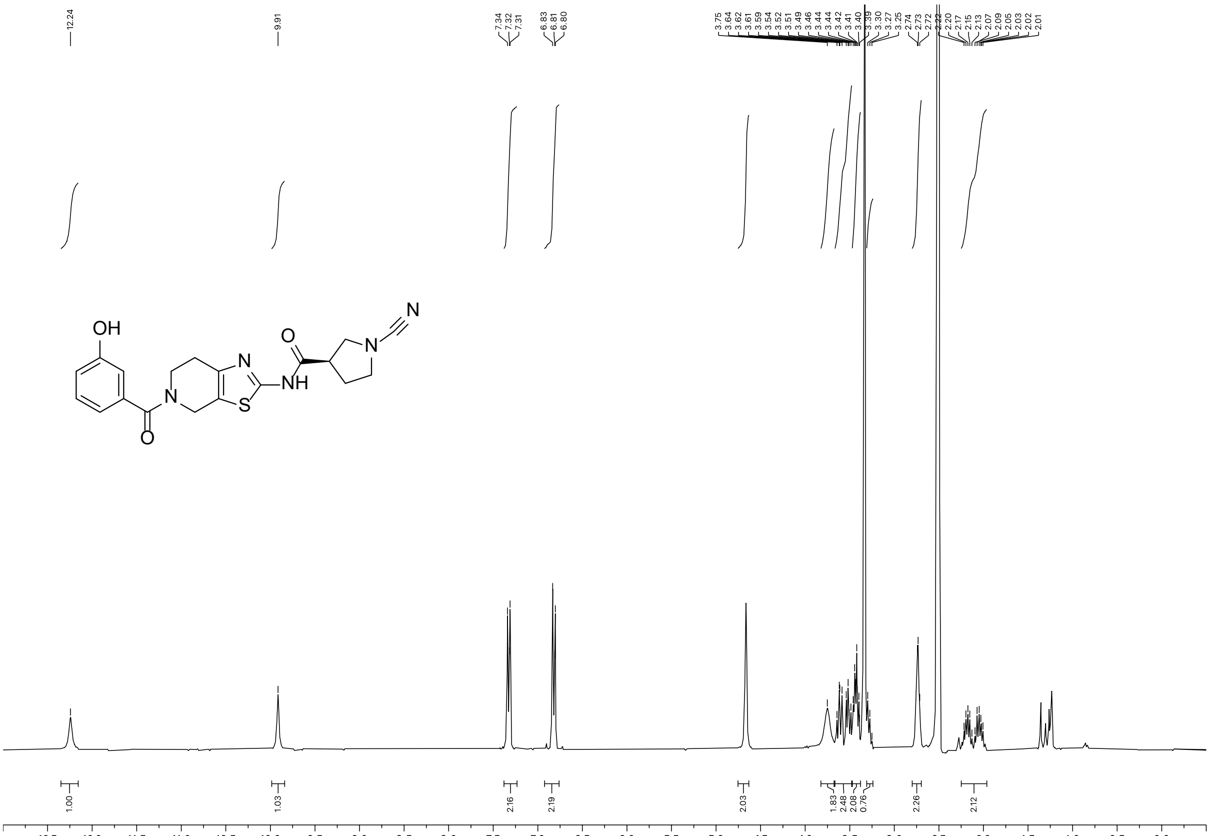

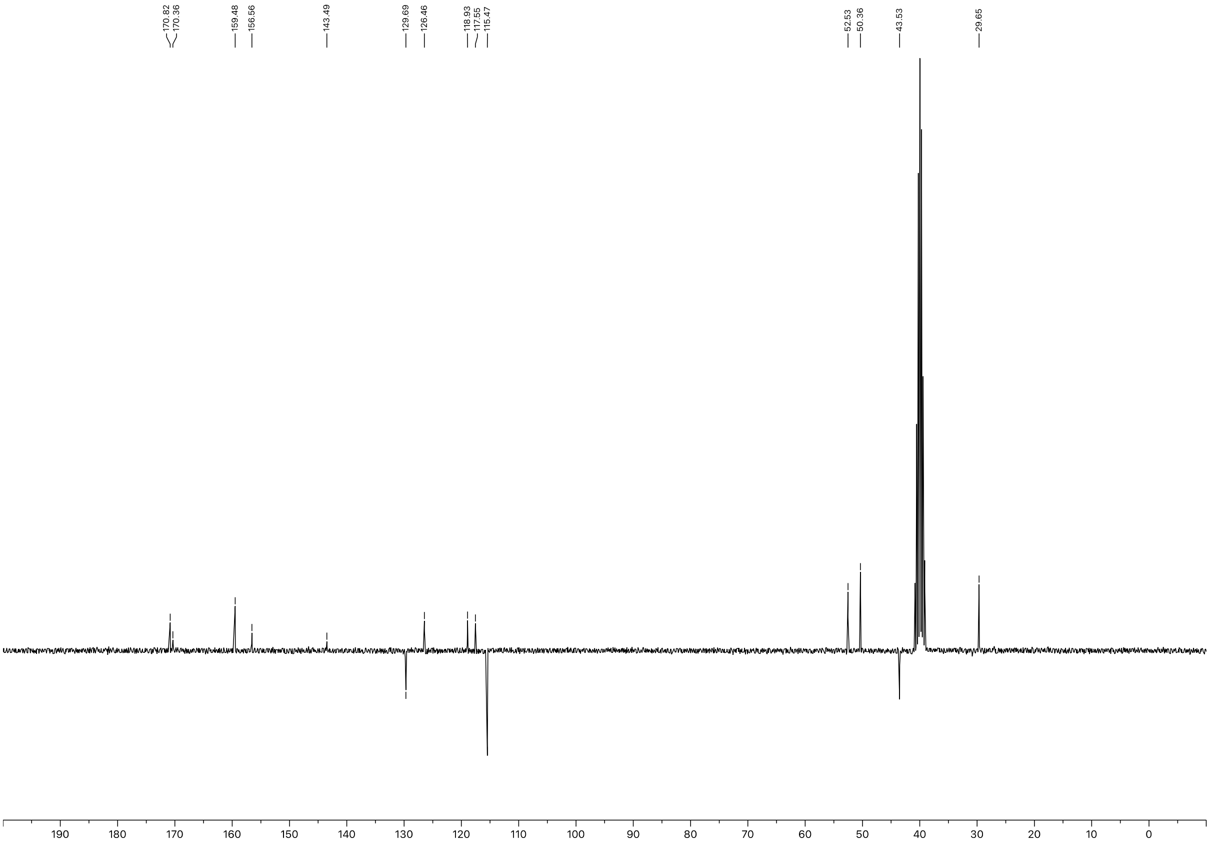

^{1}H-NMR and ^{13}C-NMR of compound 4 in CD_3OD.

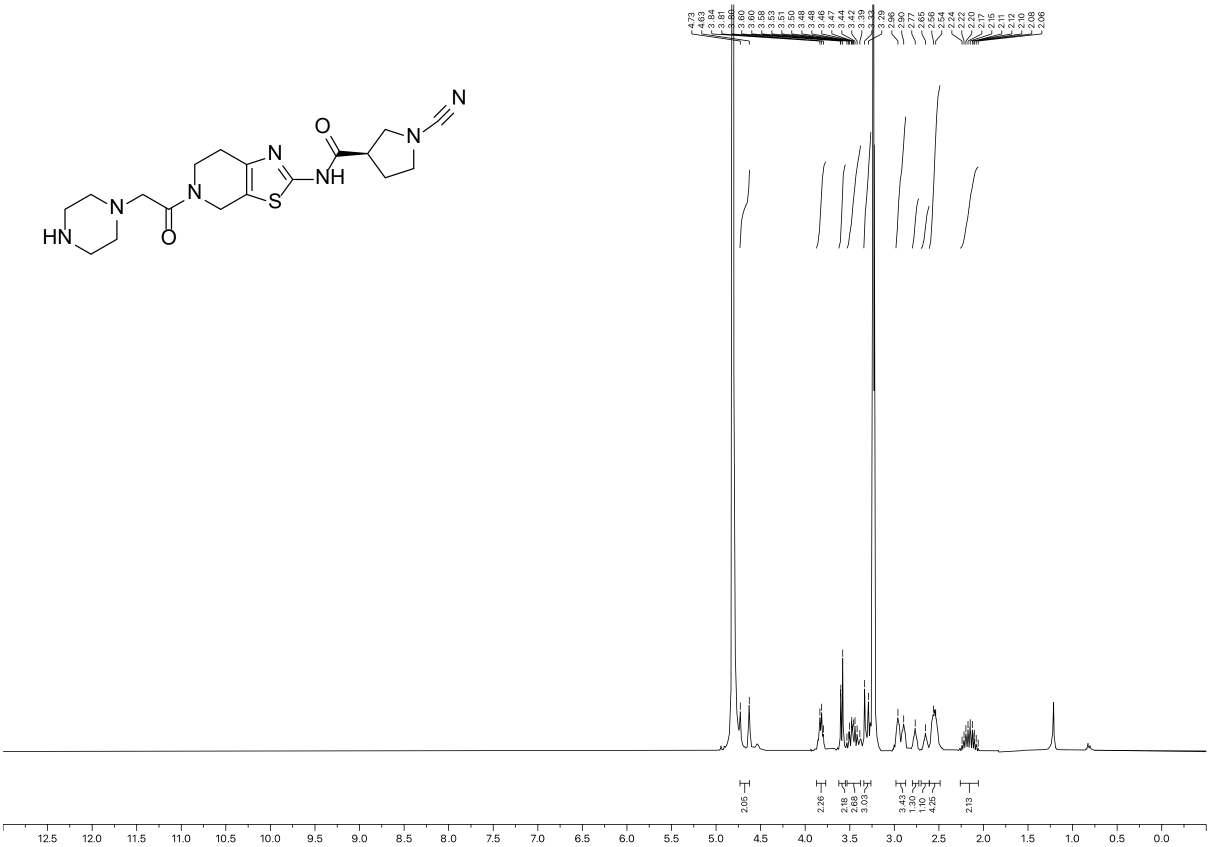

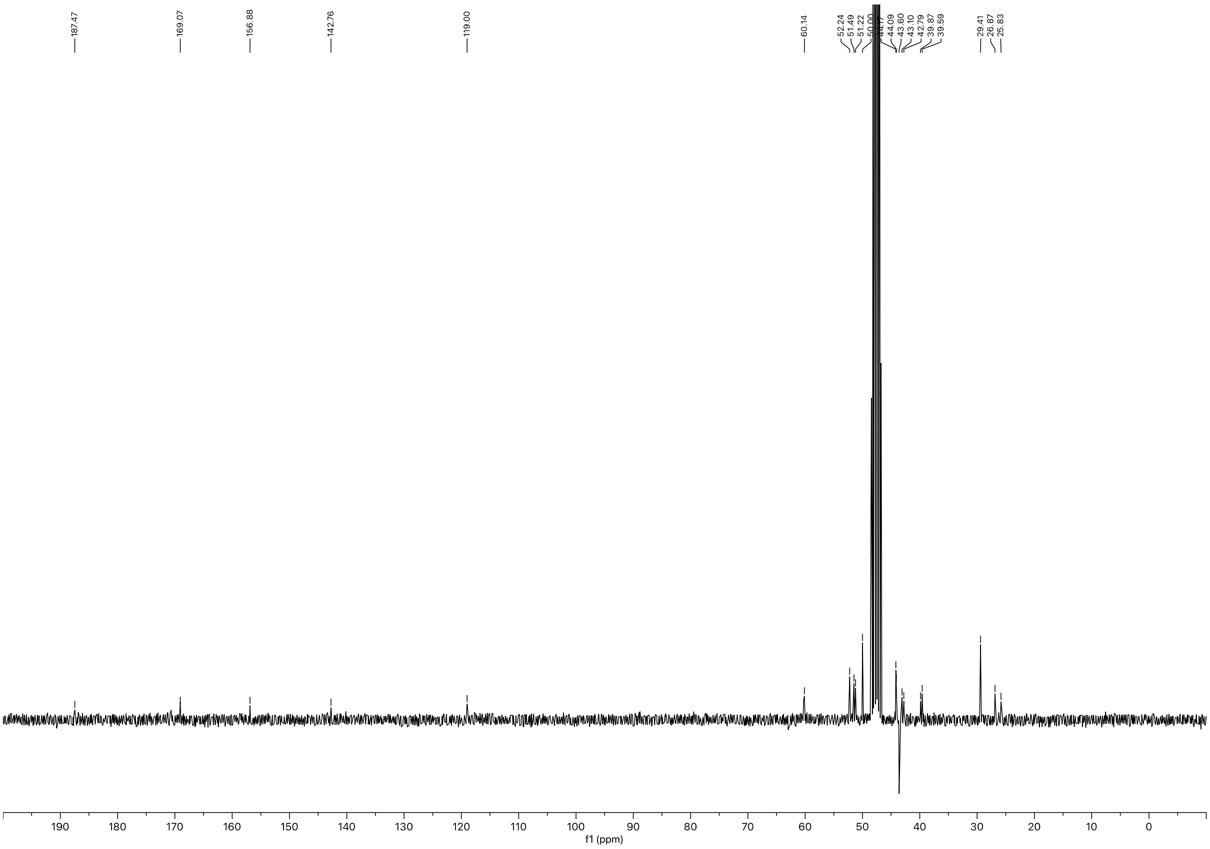

^{1}H-NMR of compound 5 in CD_3OD.

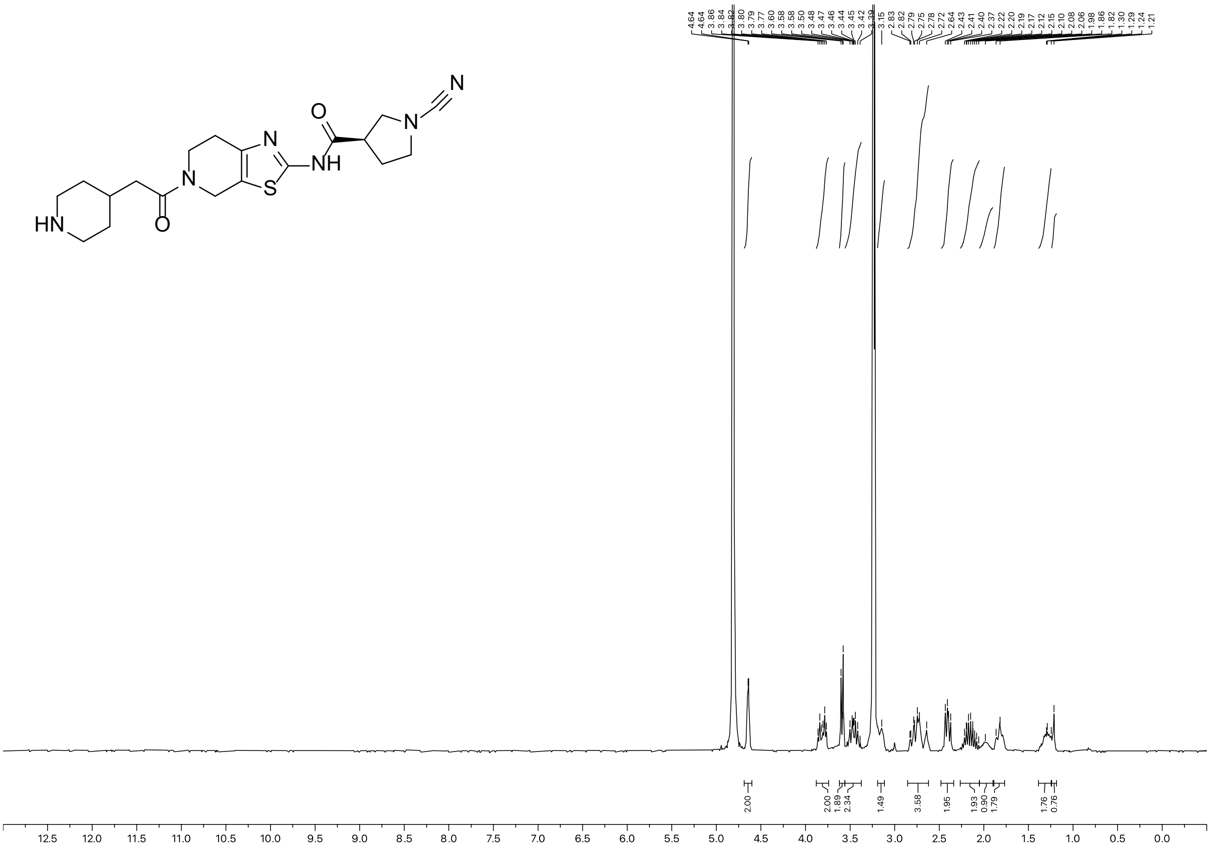

^{1}H-NMR of compound 59 in CD_3OD and ^{13}C-NMR of compound 59 in DMSO.

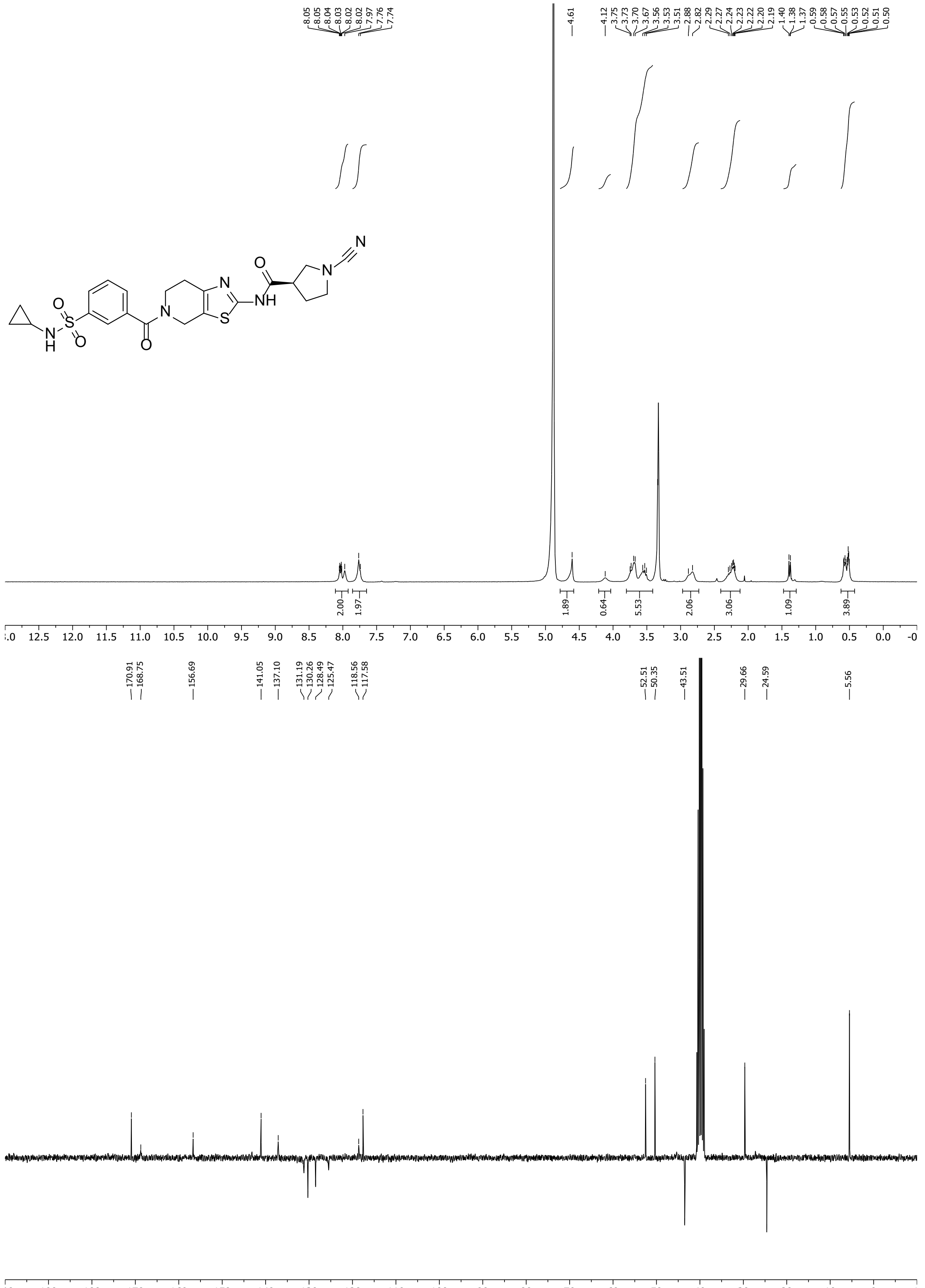

^{1}H-NMR and ^{13}C-NMR of compound 340 (JYQ-164) in DMSO.

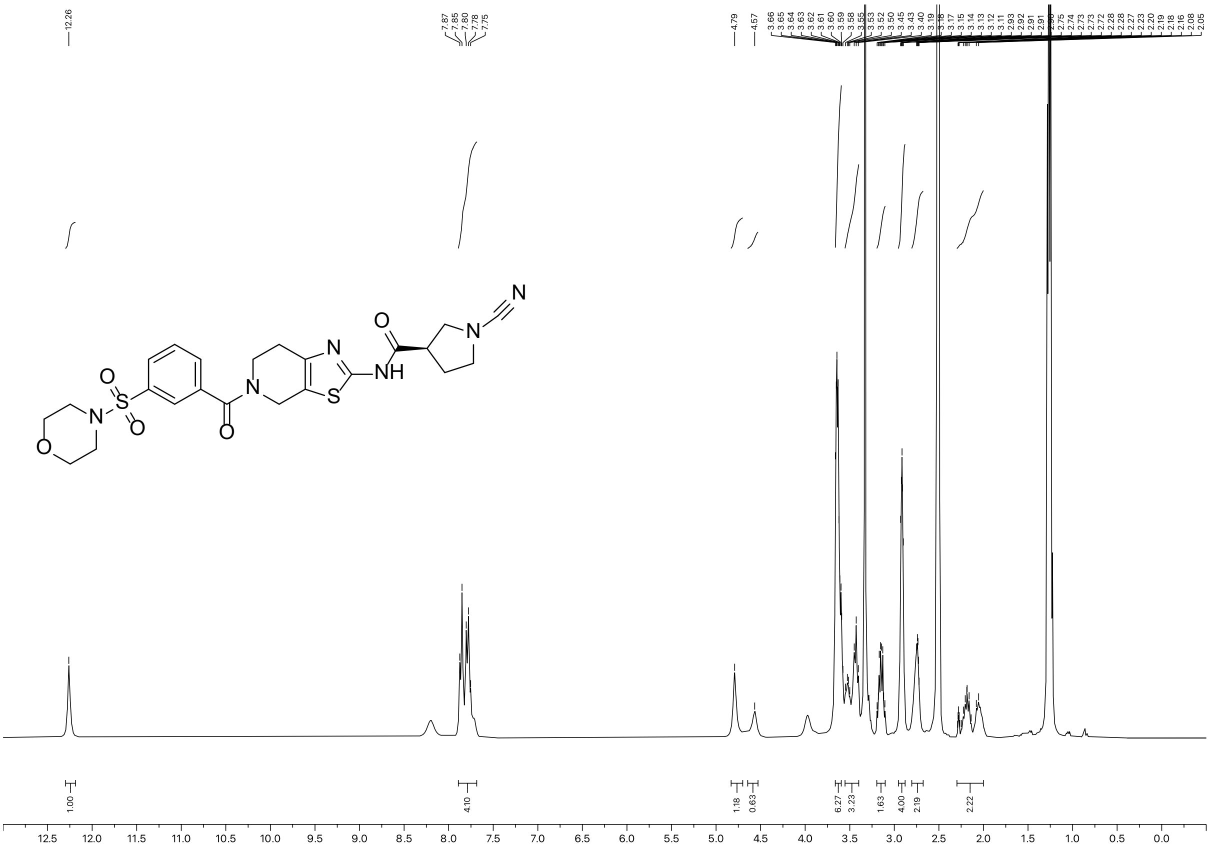

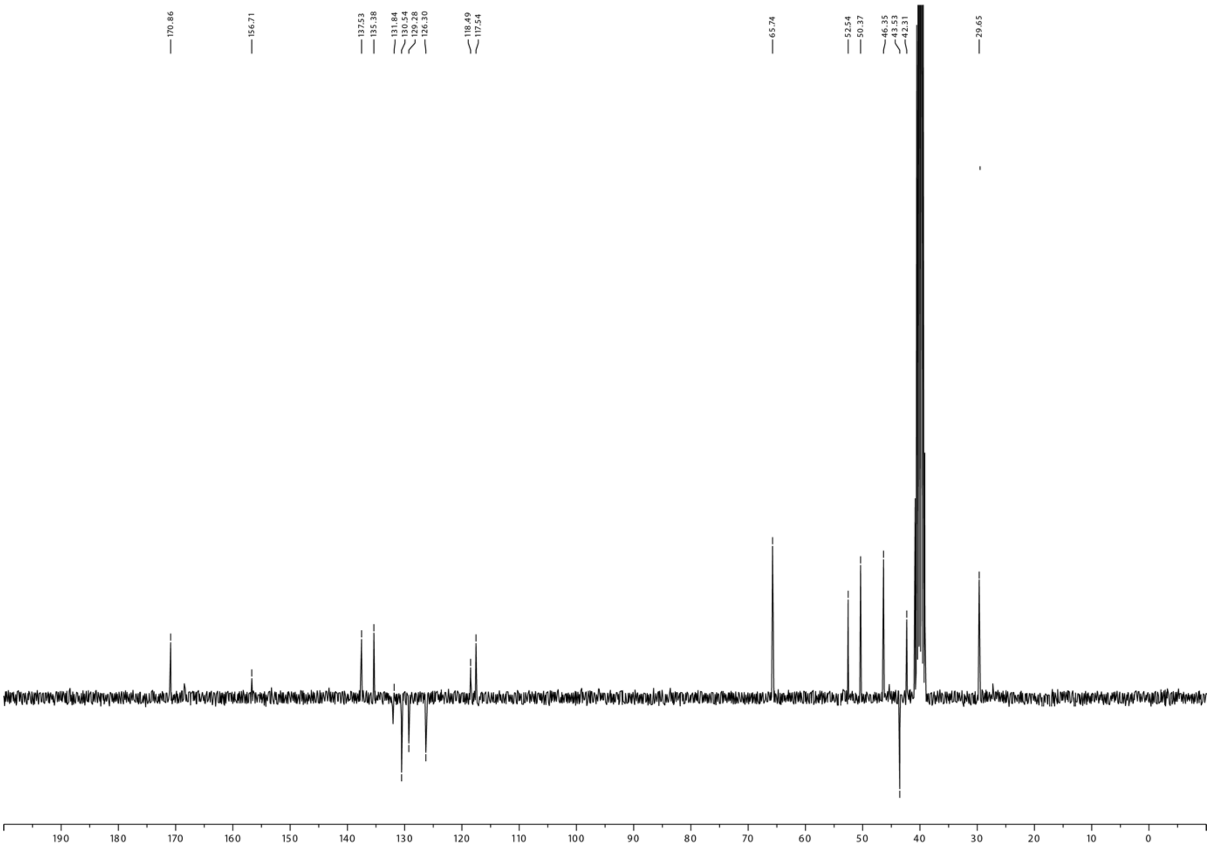

^{1}H-NMR and ^{13}C-NMR of compound 415 in DMSO.

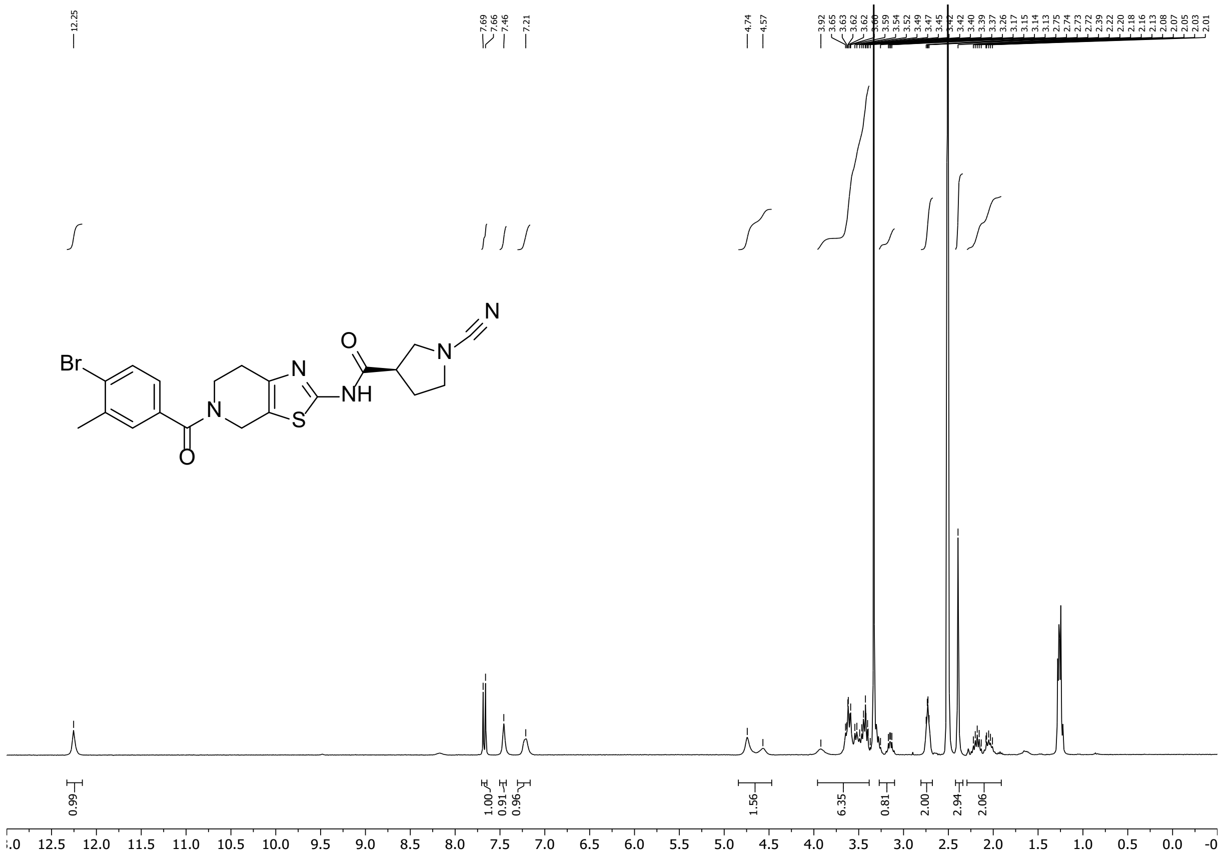

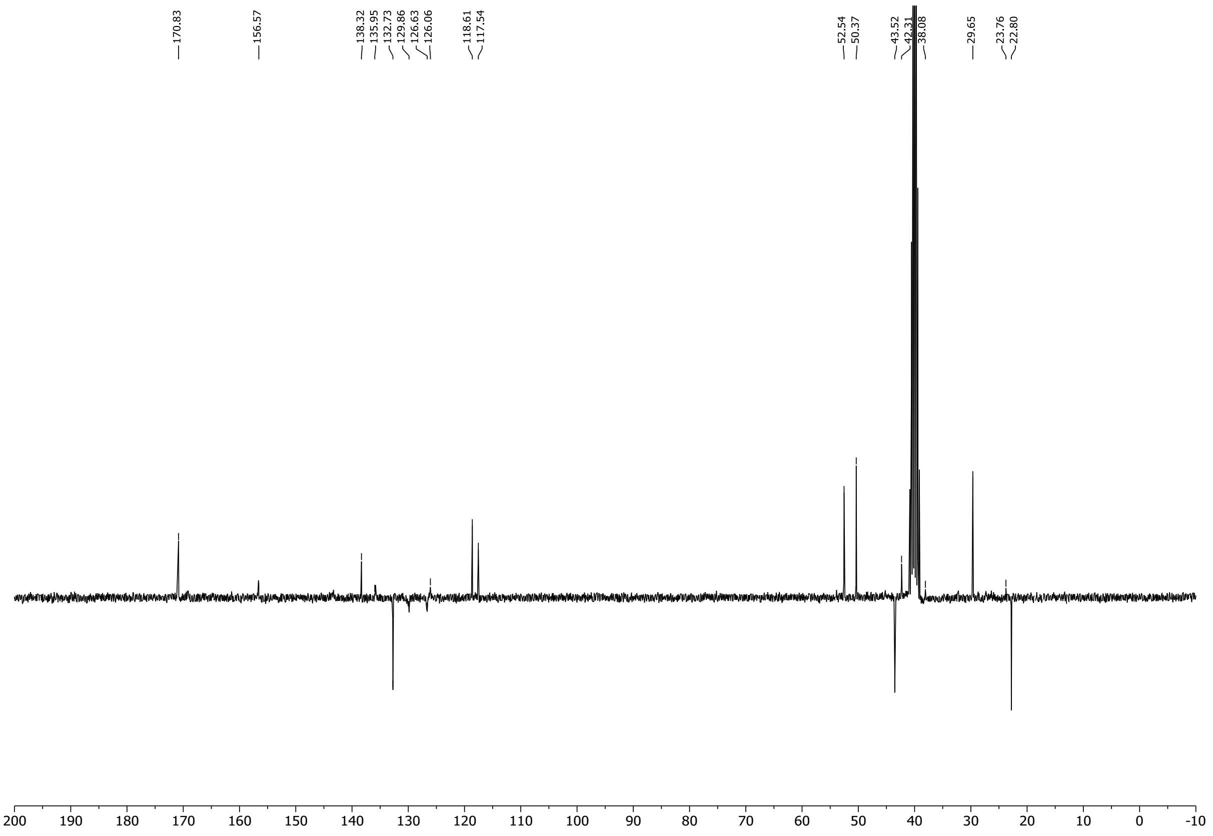

^{1}H-NMR and ^{13}C-NMR of compound 336 in DMSO.

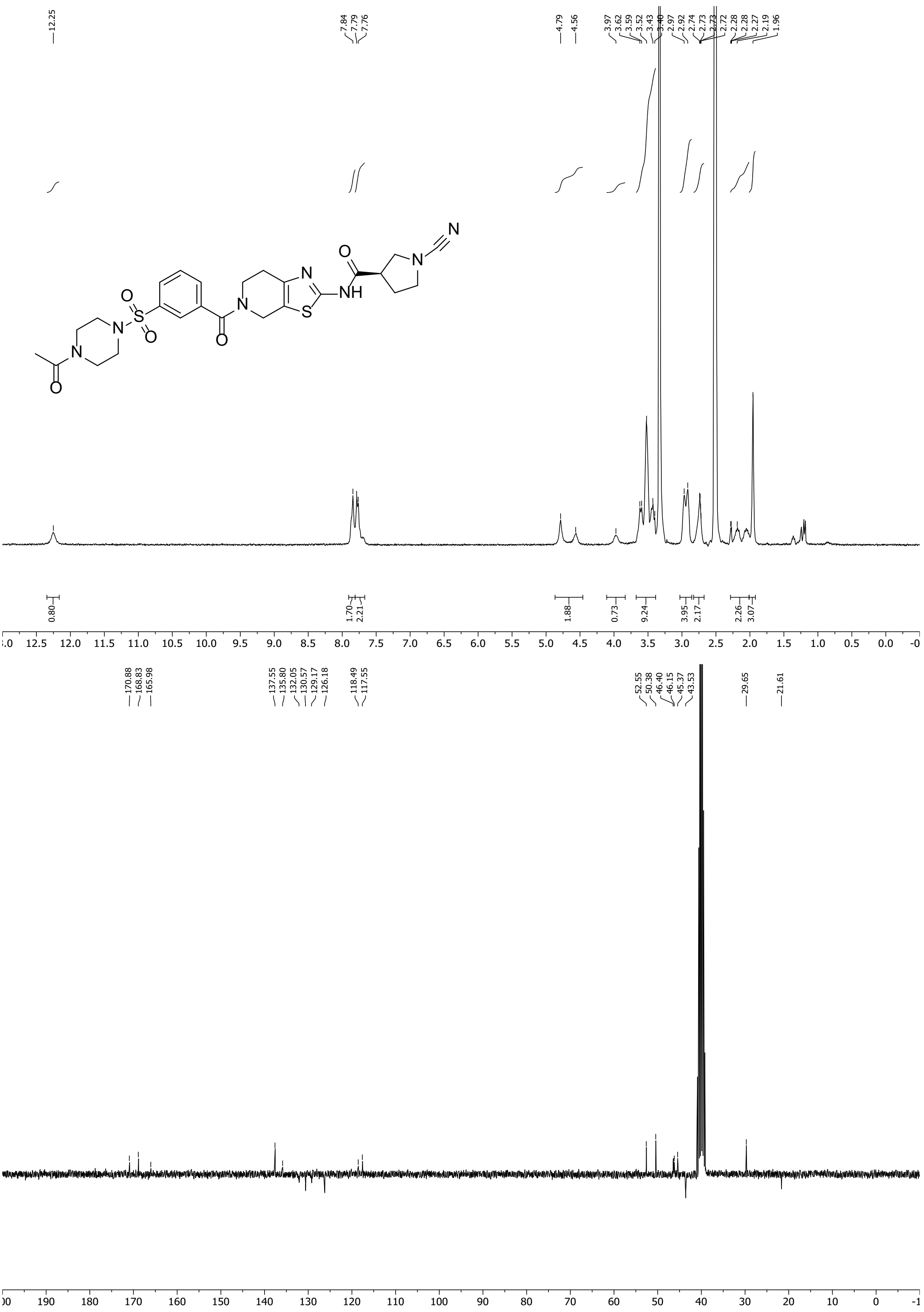

^{1}H-NMR and ^{13}C-NMR of compound 133 in DMSO.

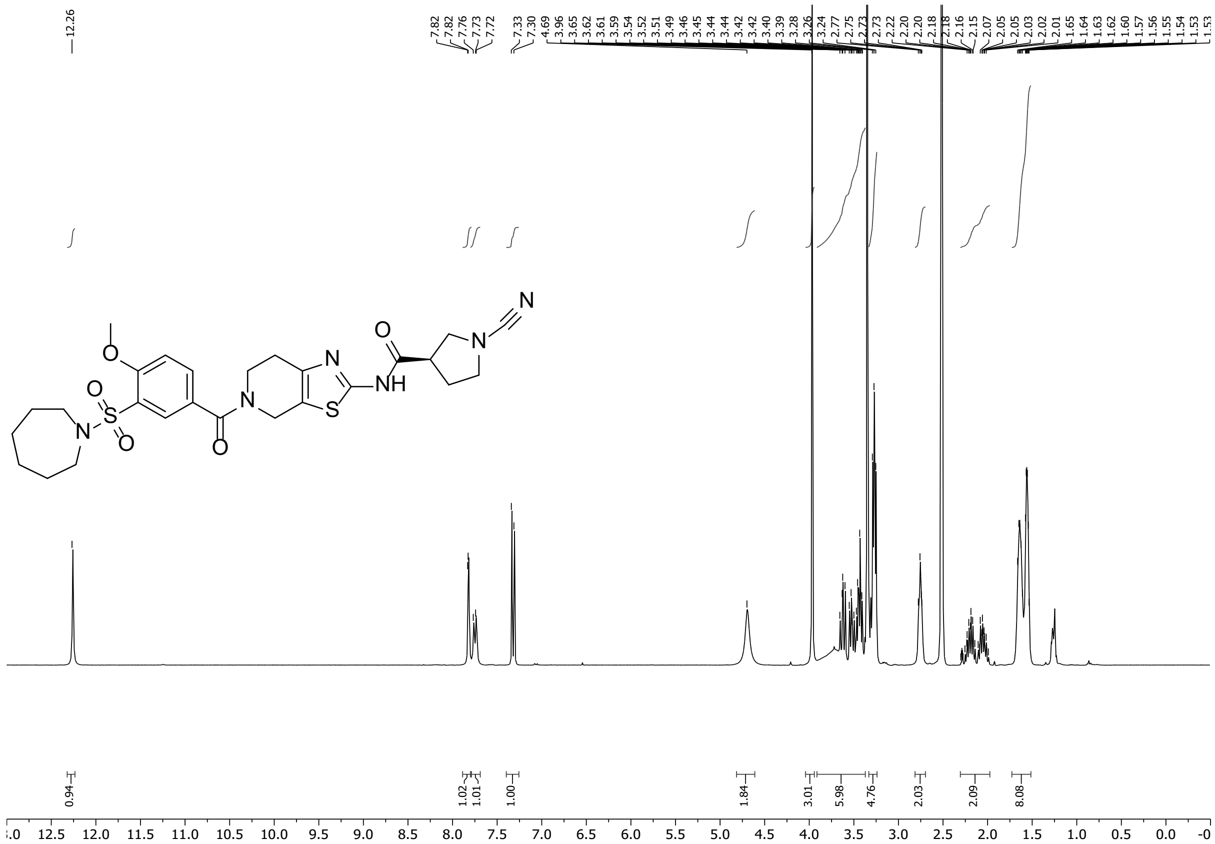

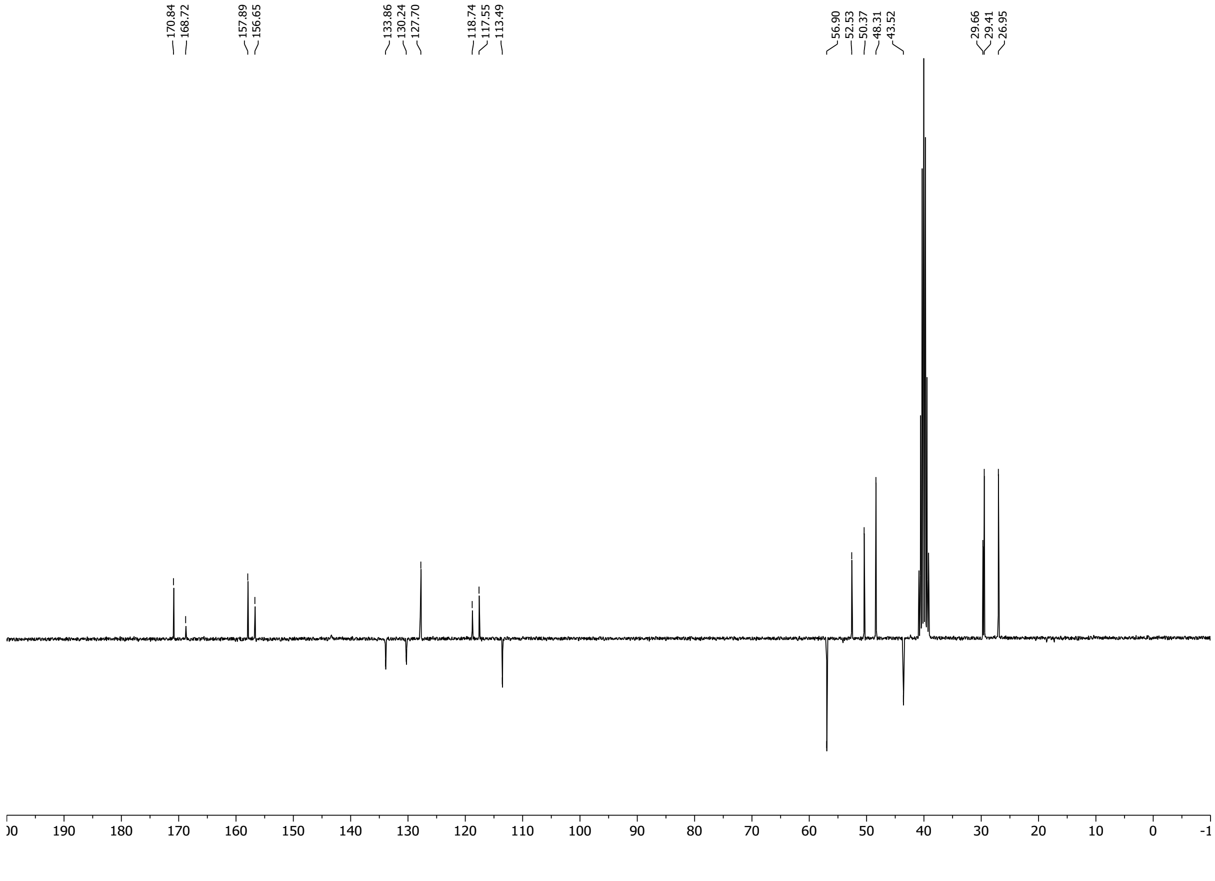

^{1}H-NMR and ^{13}C-NMR of compound 55 in DMSO.

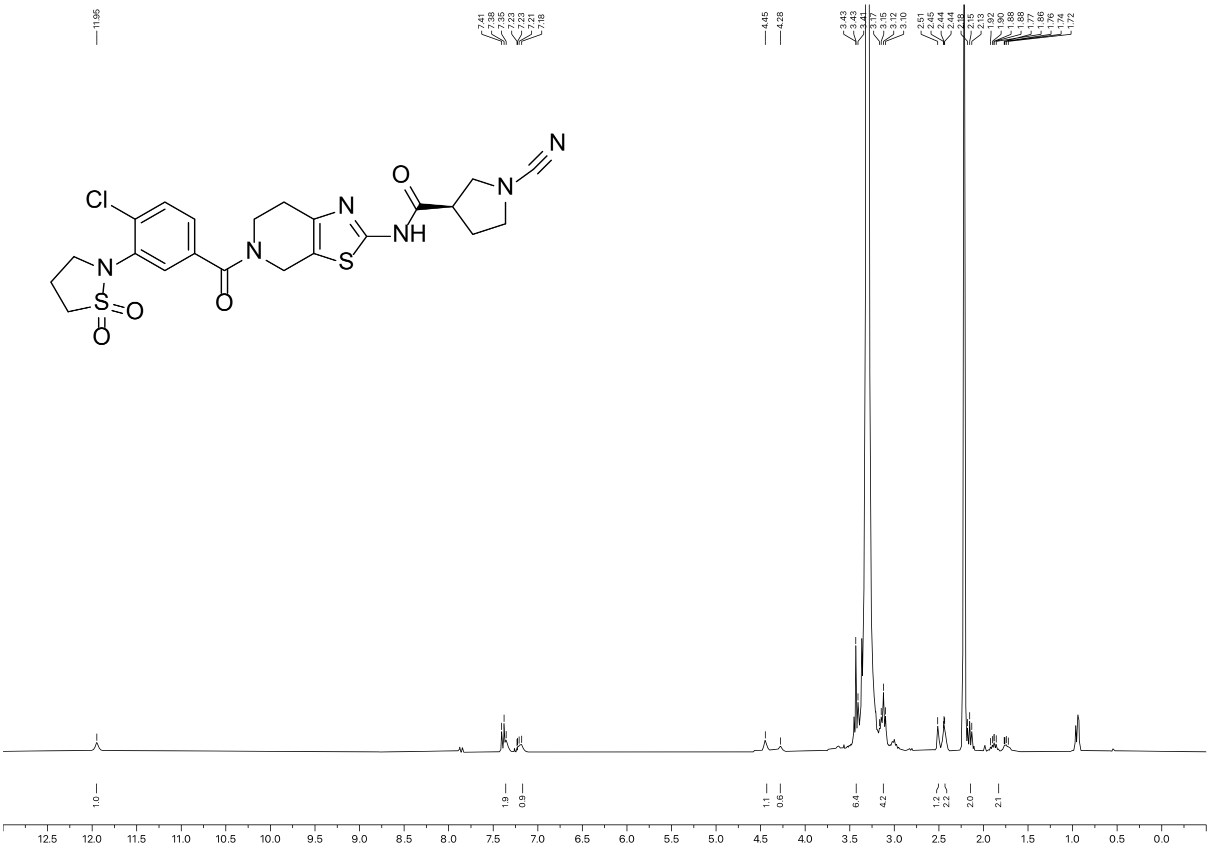

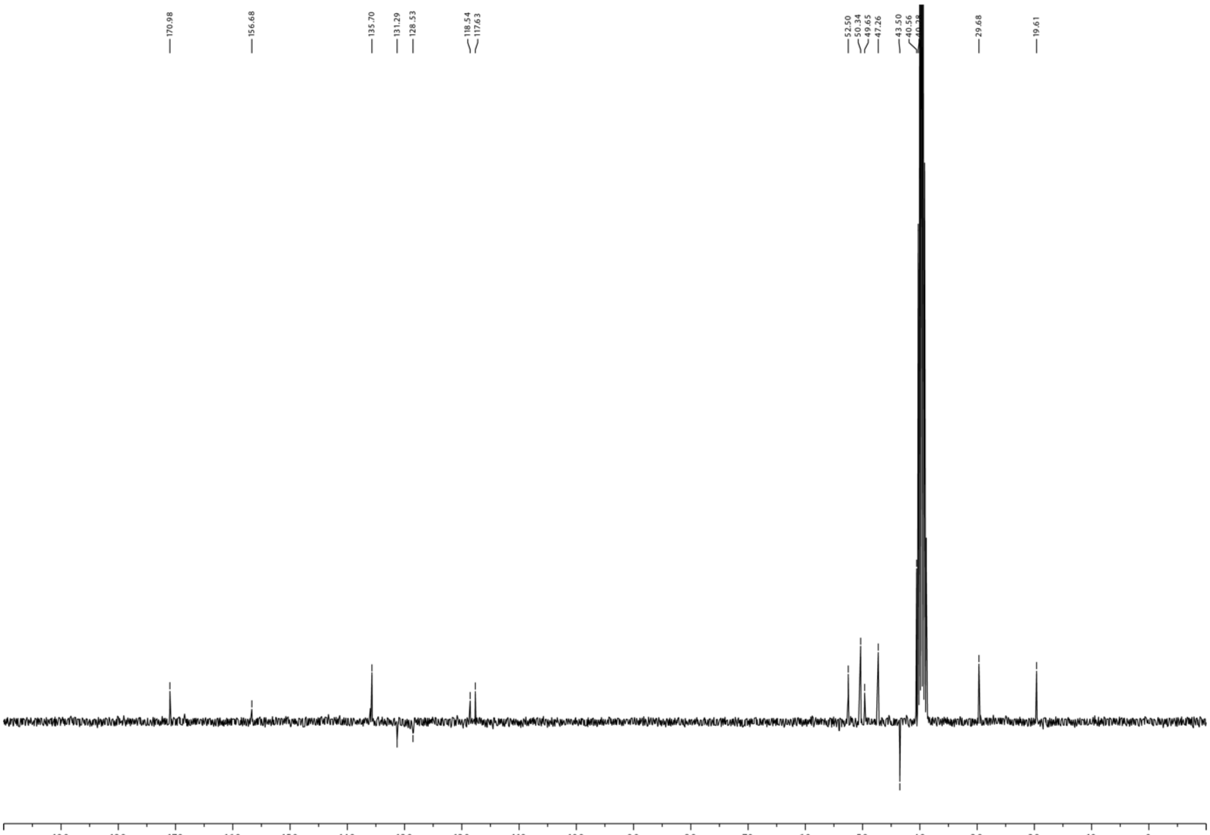

^{1}H-NMR and ^{13}C-NMR of compound 108 in DMSO.

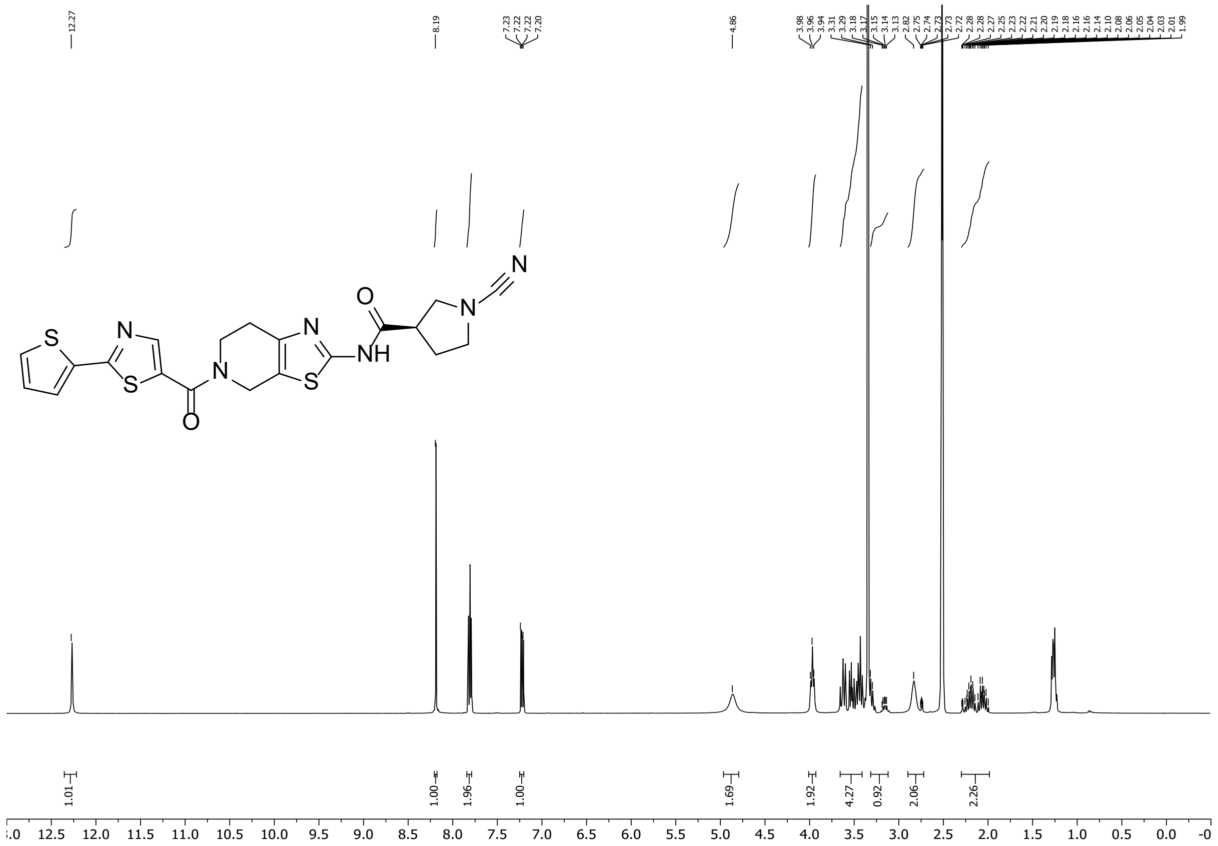

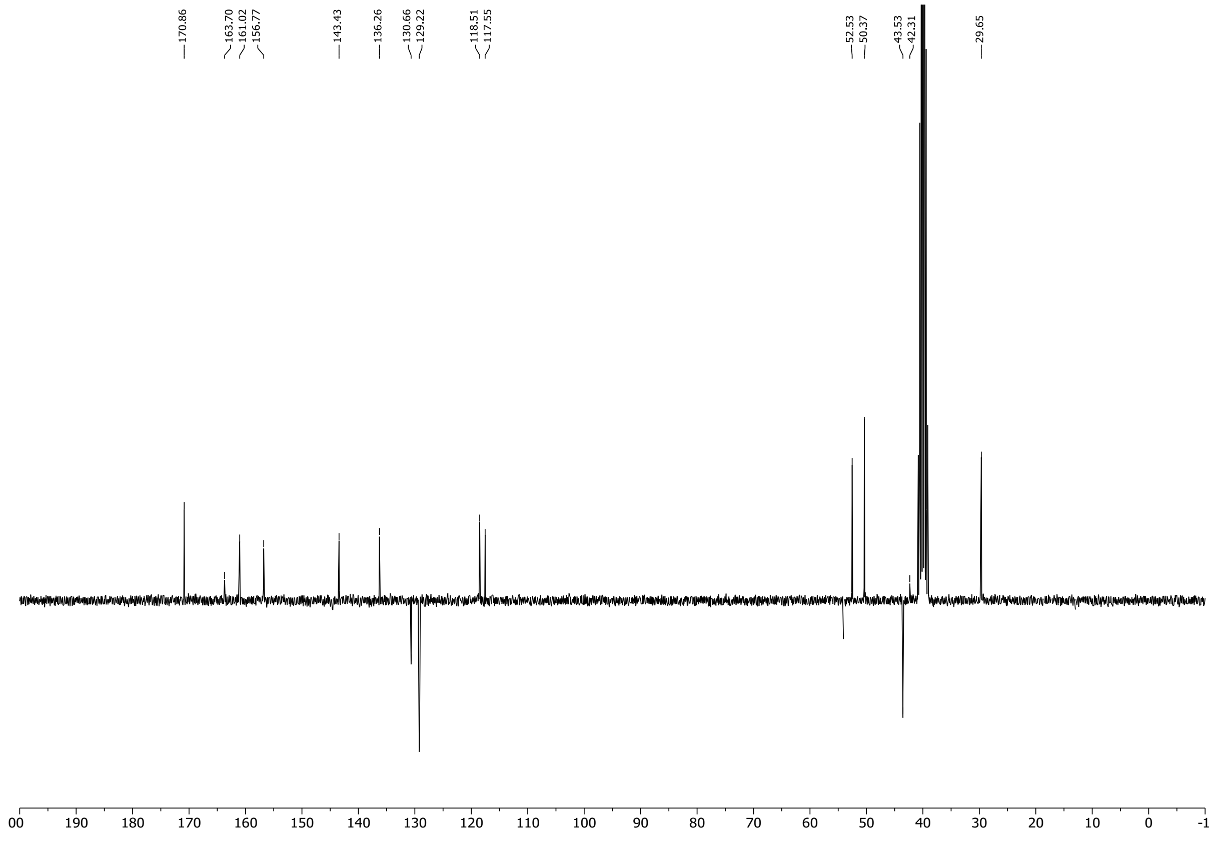

^{1}H-NMR and ^{13}C-NMR of compound 334 in DMSO.

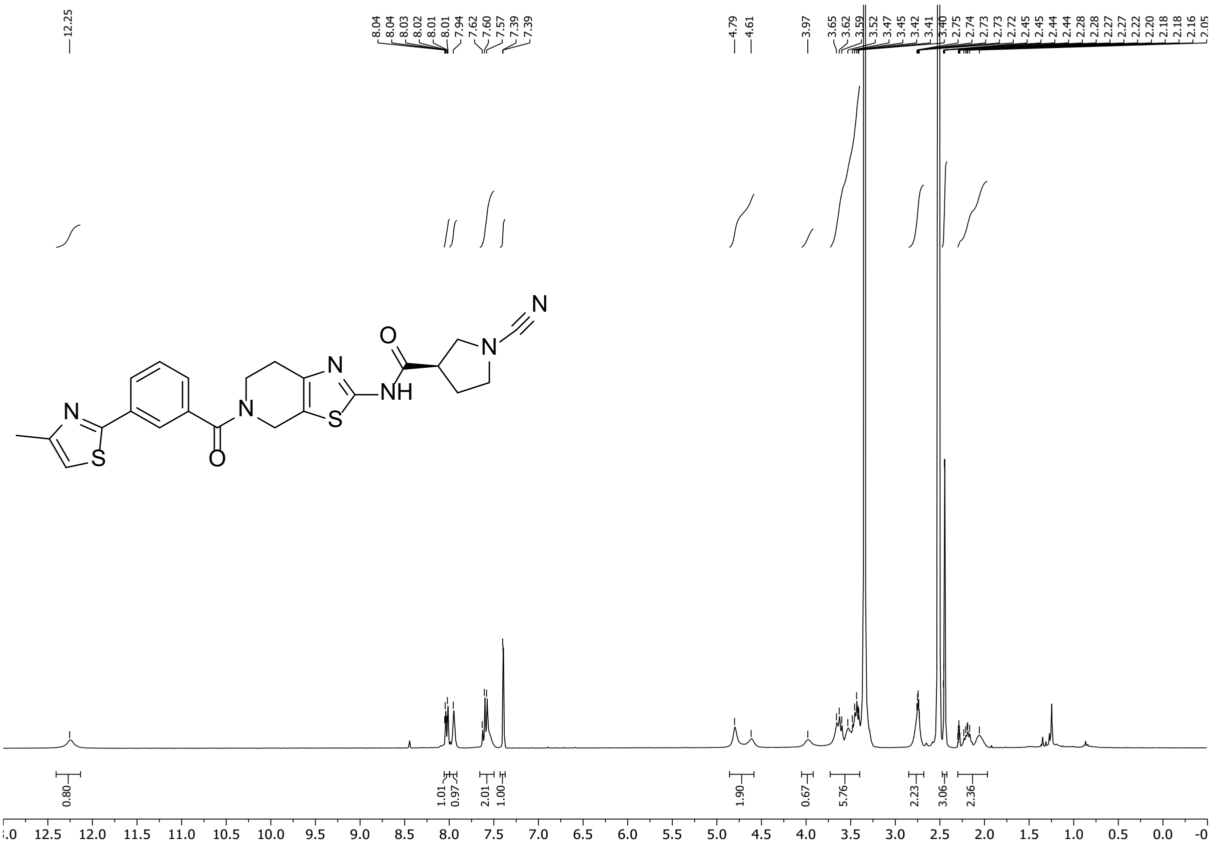

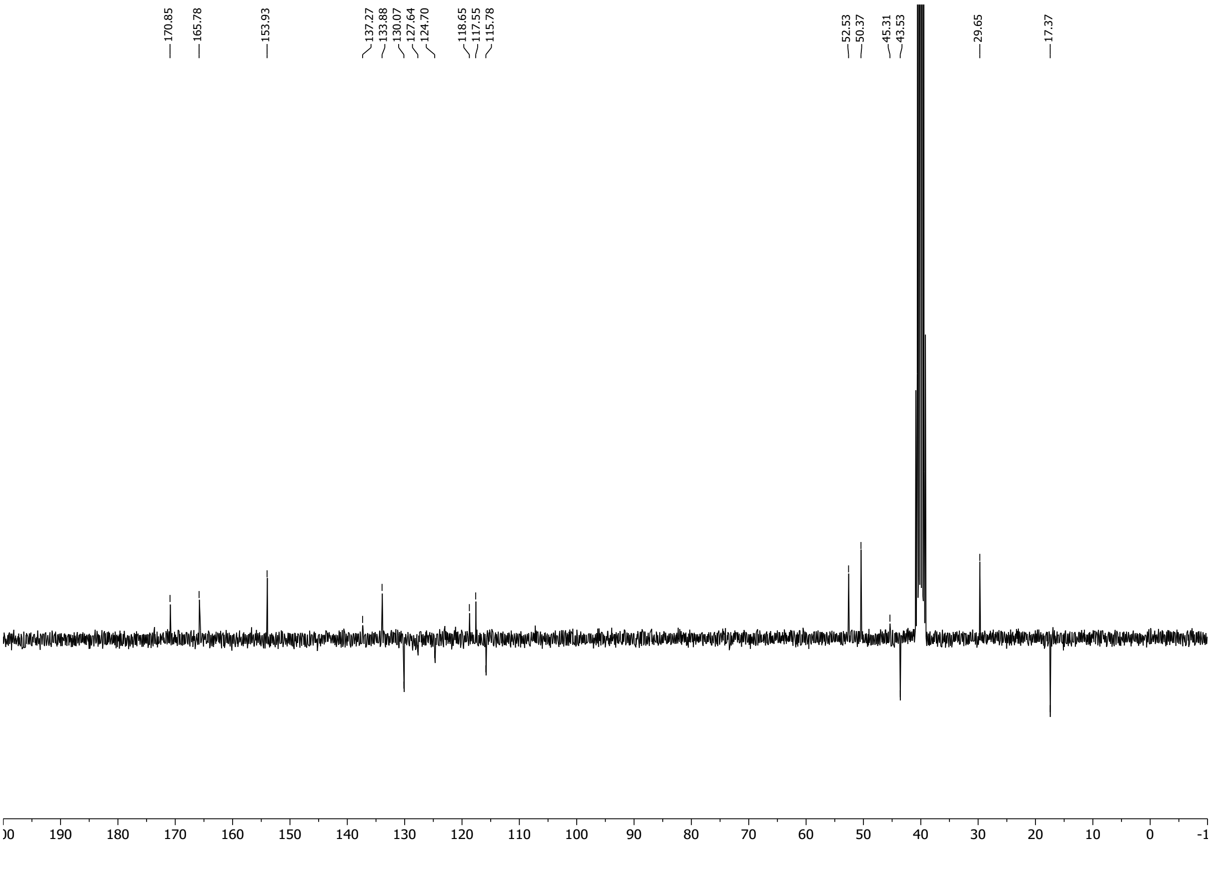

^{1}H-NMR and ^{13}C-NMR of compound 199 in DMSO.

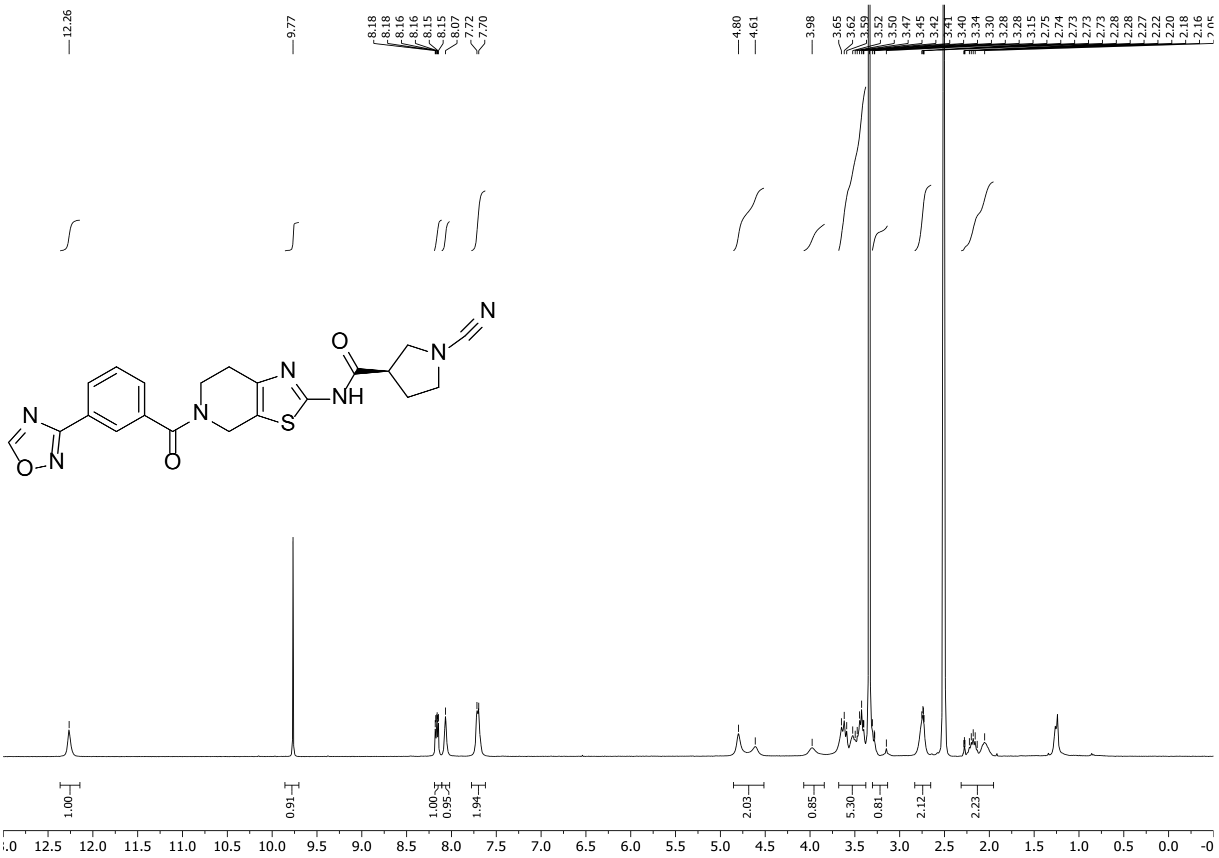

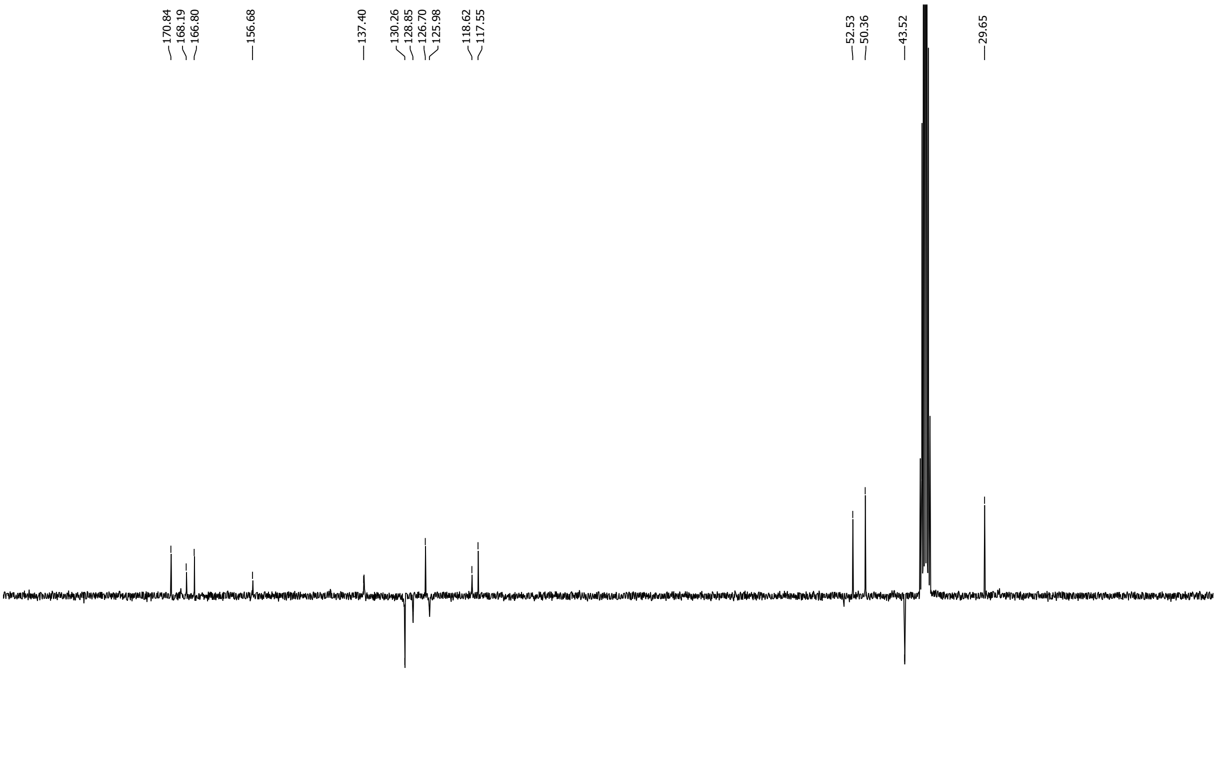

^{1}H-NMR and ^{13}C-NMR of compound 84 (JYQ-173) in DMSO.

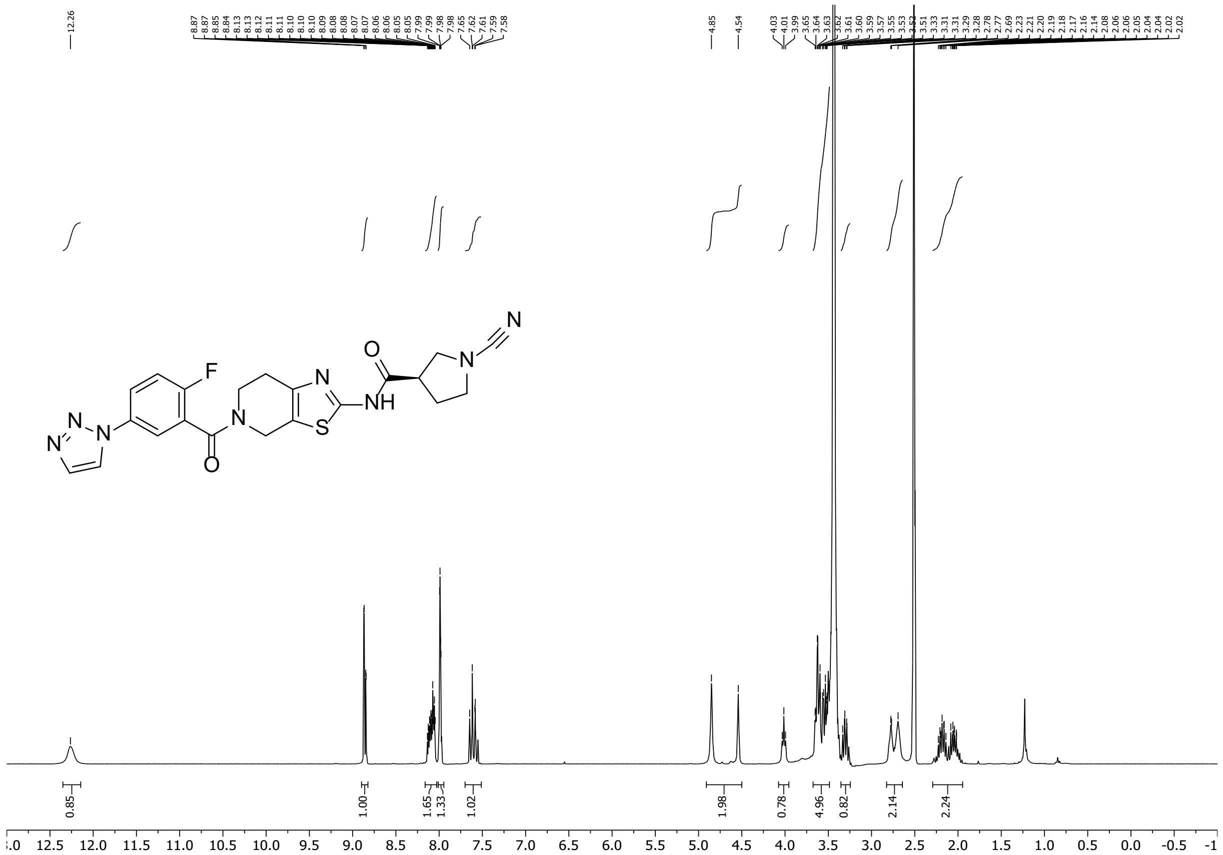

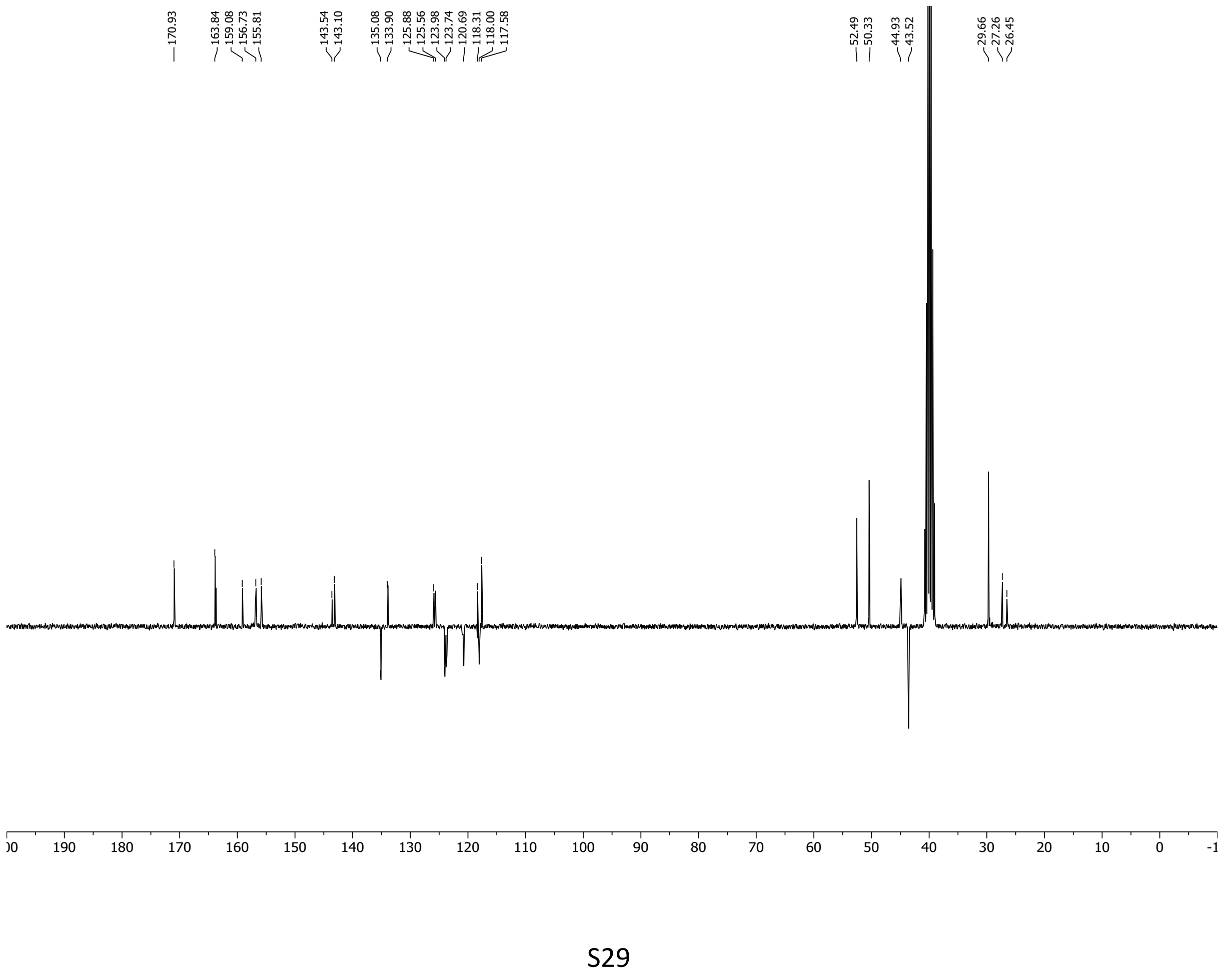

^{1}H-NMR and ^{13}C-NMR of compound 482 in DMSO.

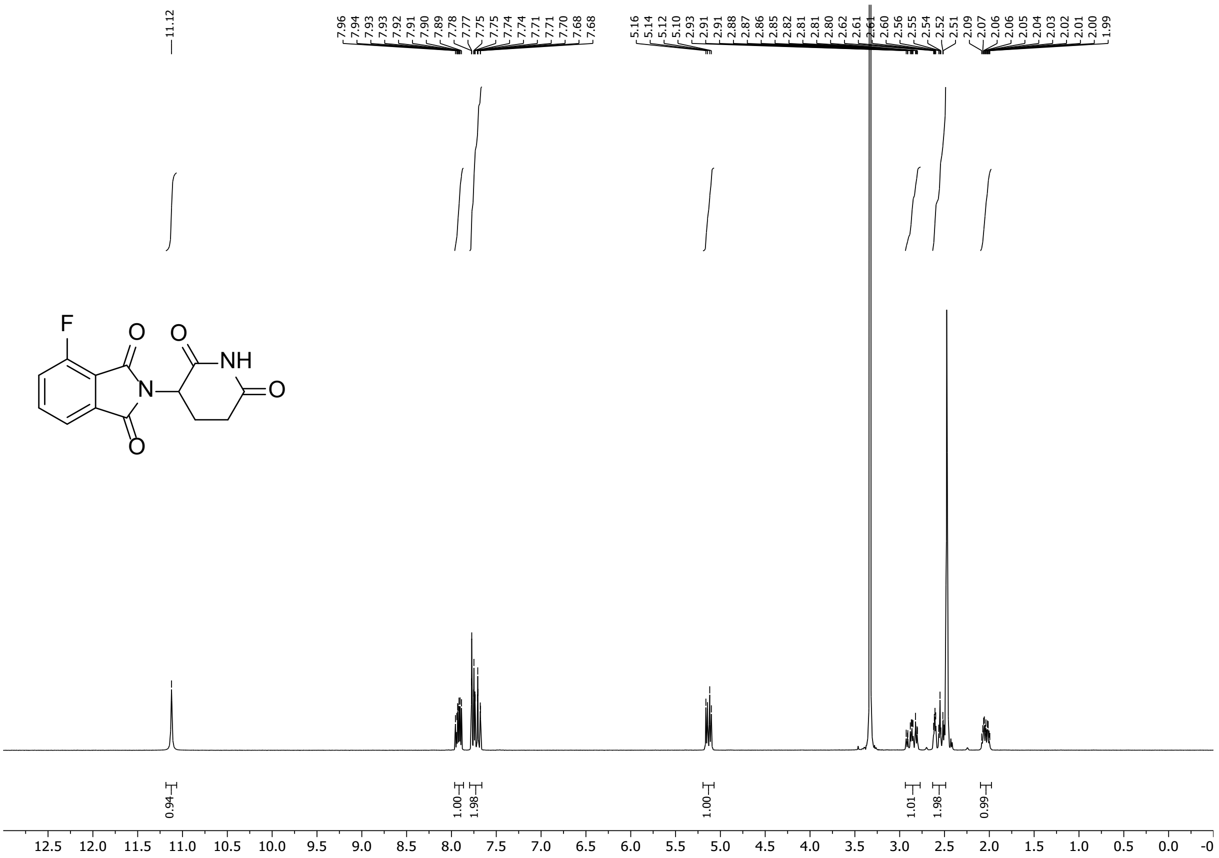

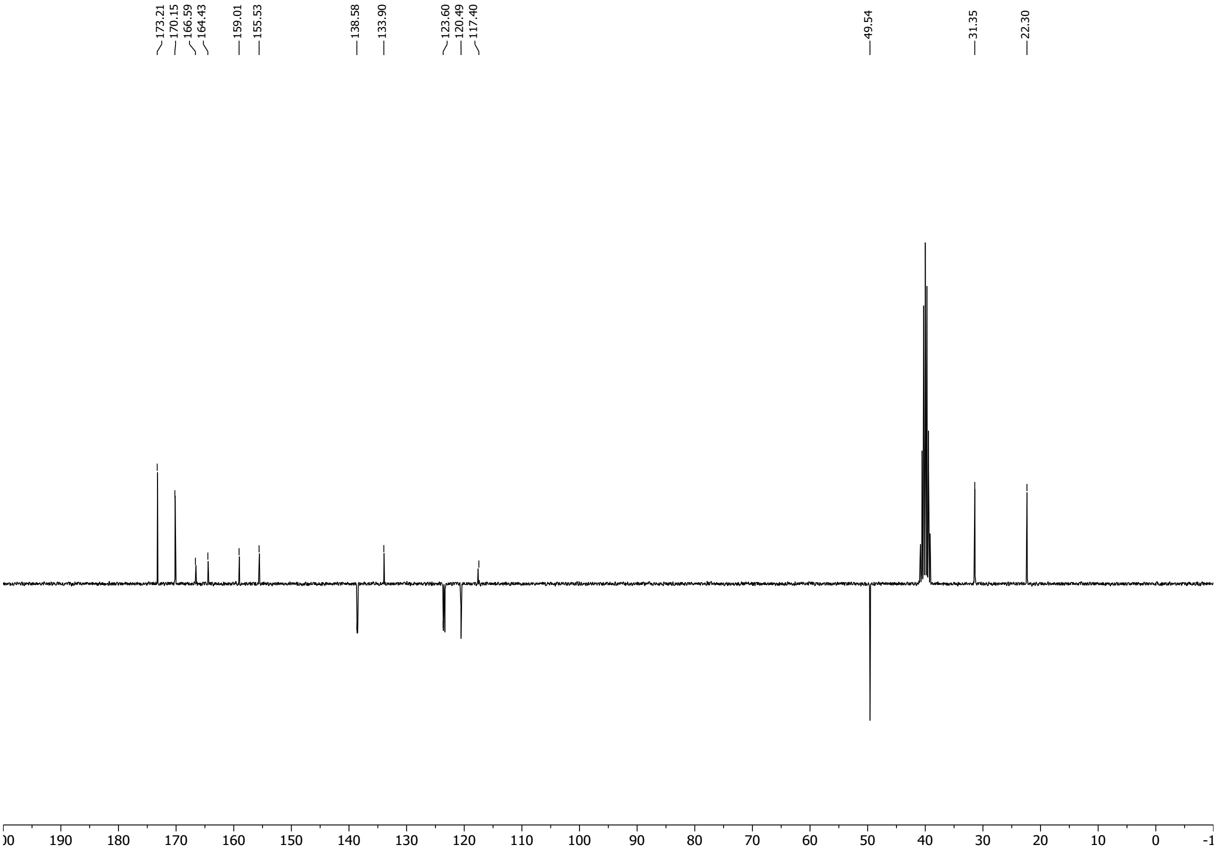

^{1}H-NMR and ^{13}C-NMR of compound 483a in $CDCl_3$.

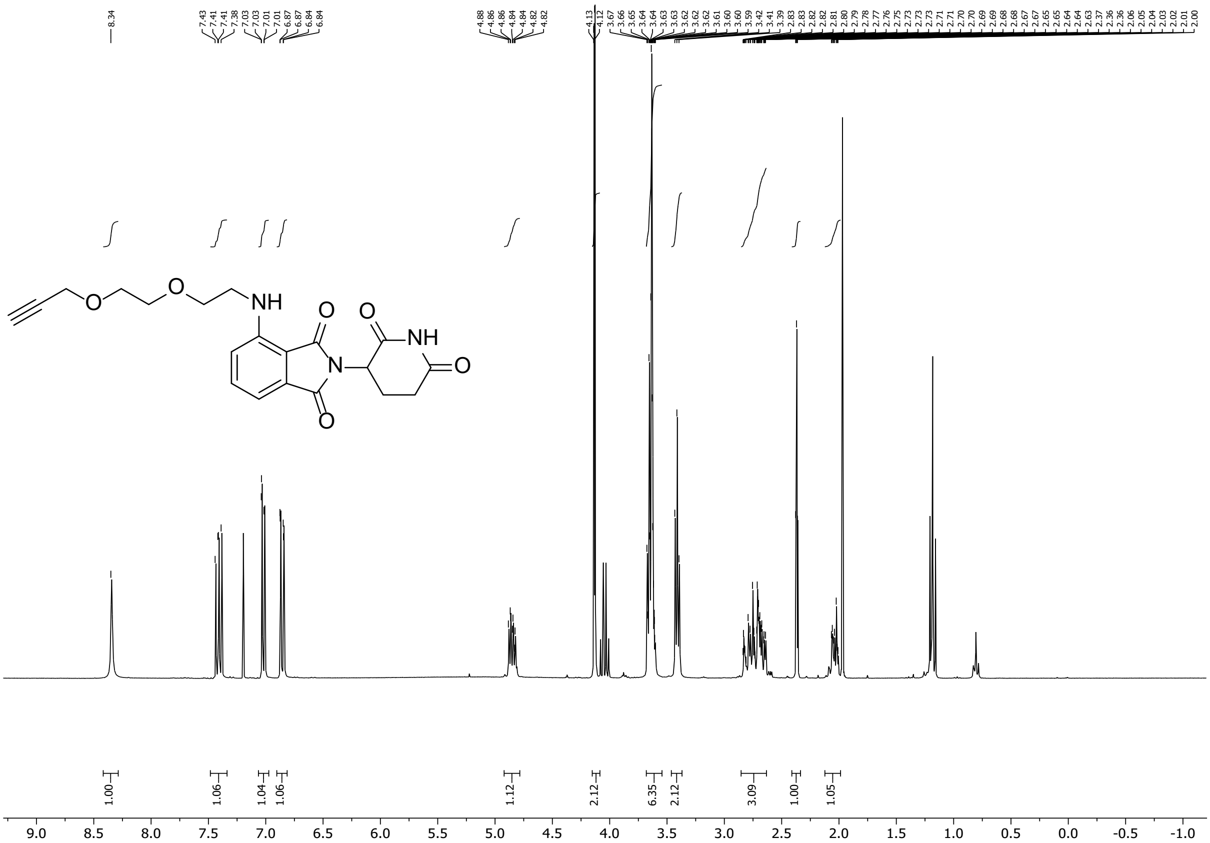

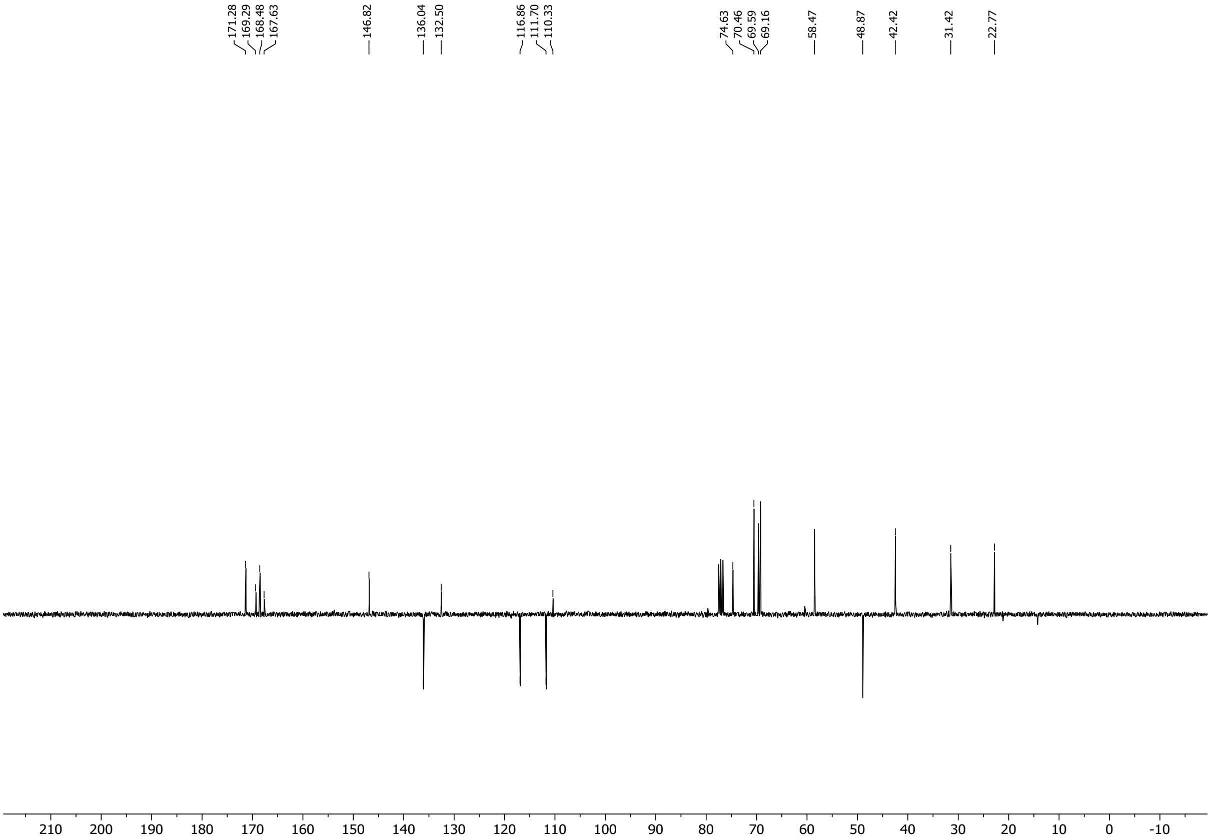

^{1}H-NMR and ^{13}C-NMR of compound 477 in $CDCl_3$.

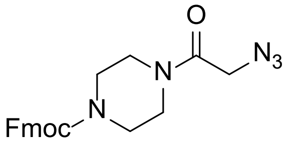

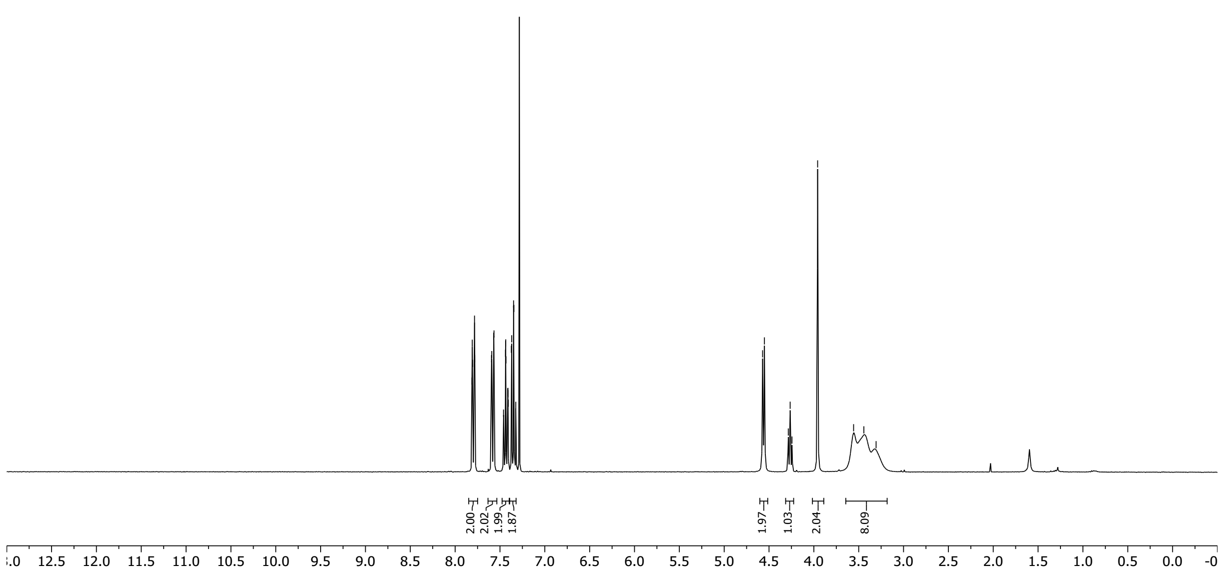

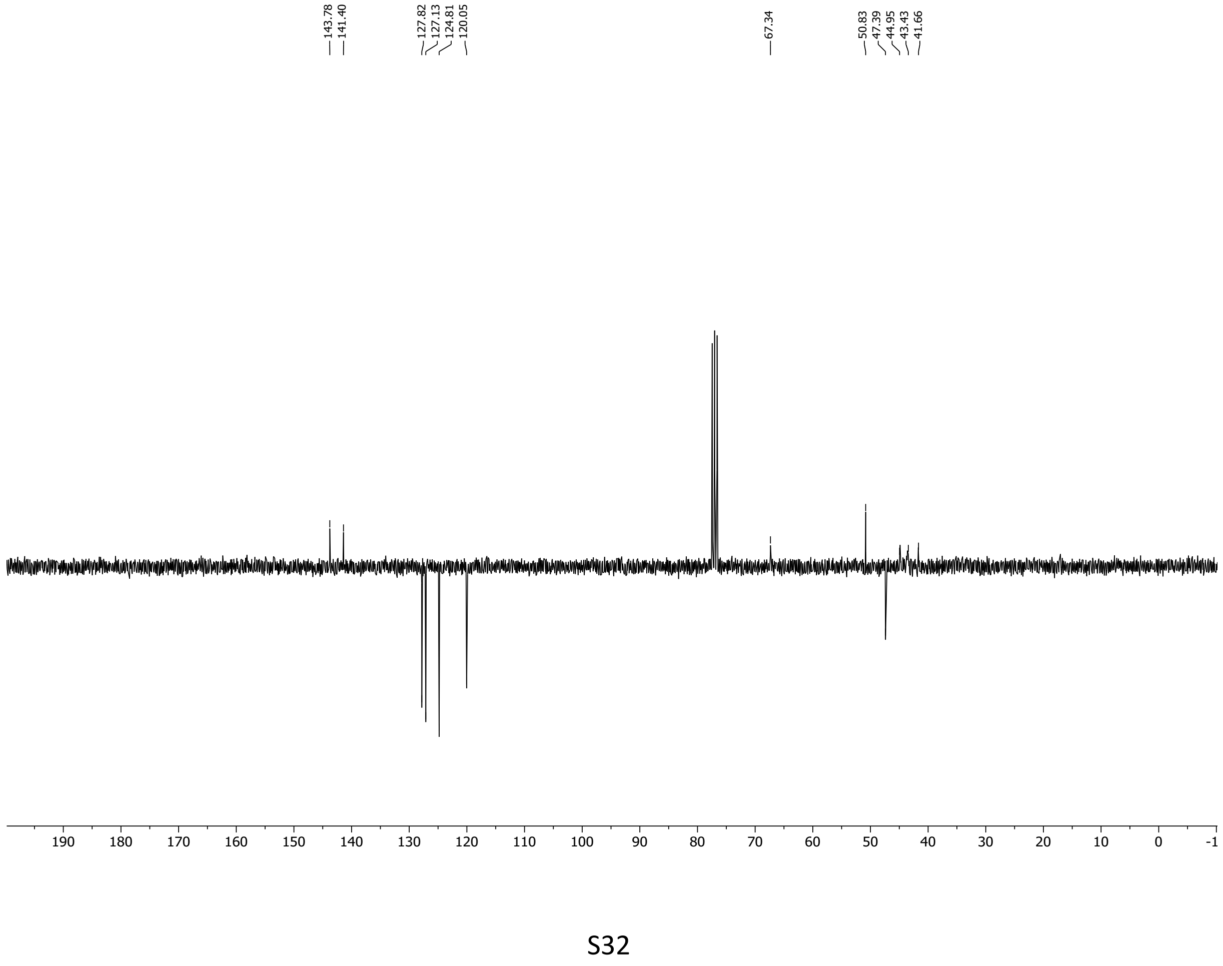

^{1}H-NMR and ^{13}C-NMR of compound 479 in DMSO.

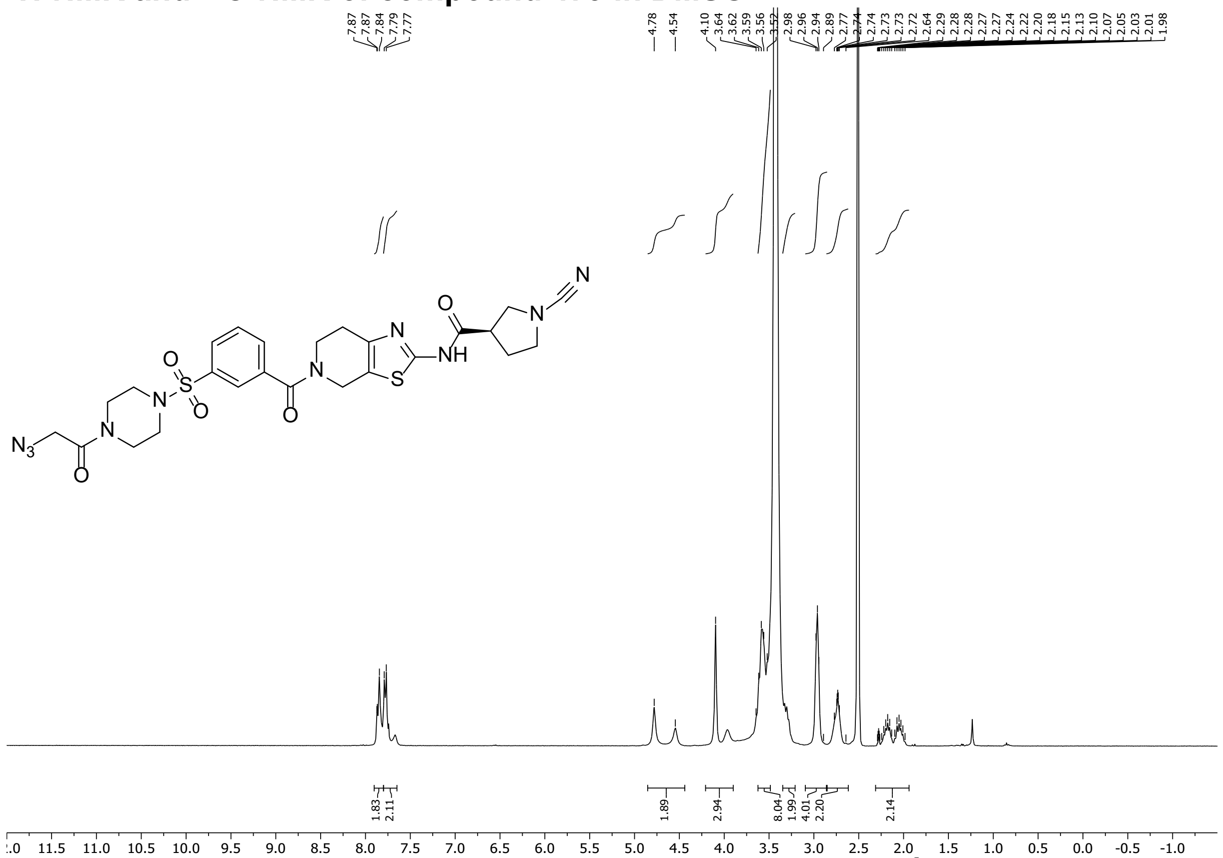

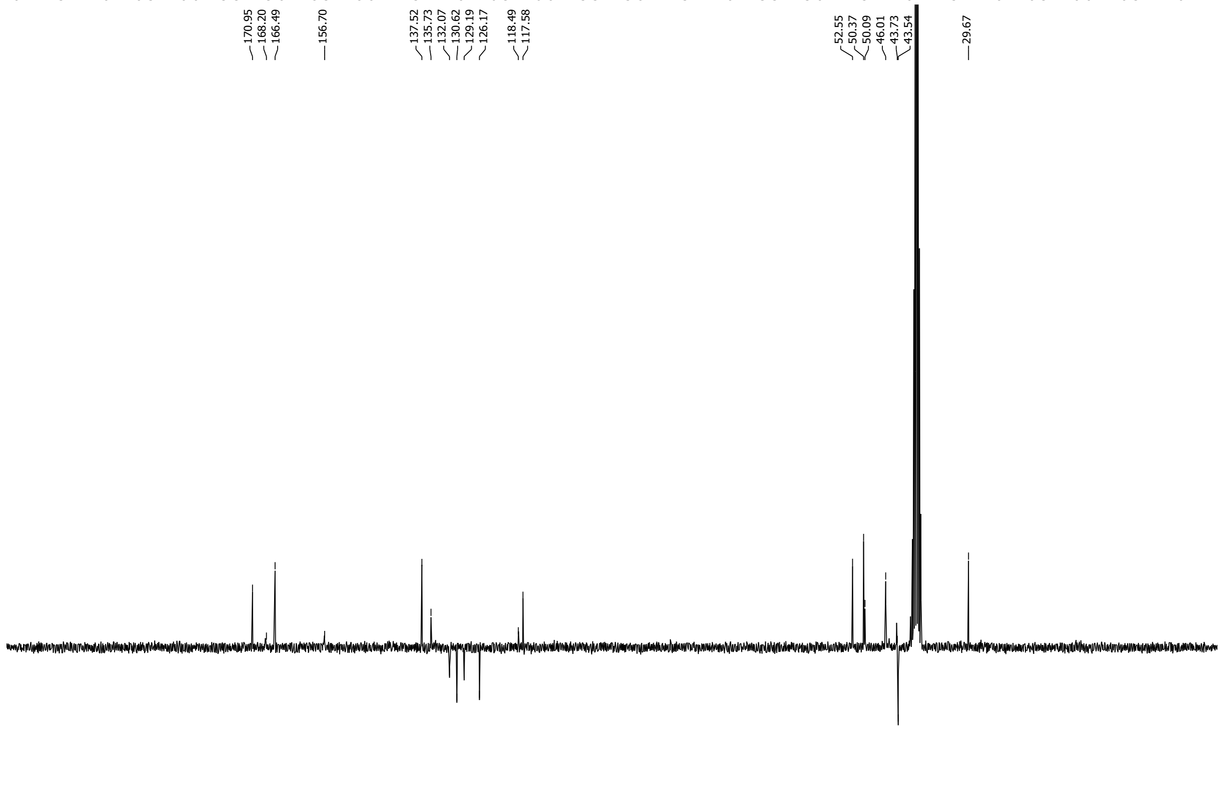

^{1}H-NMR and ^{13}C-NMR of compound JYQ-187 in DMSO.

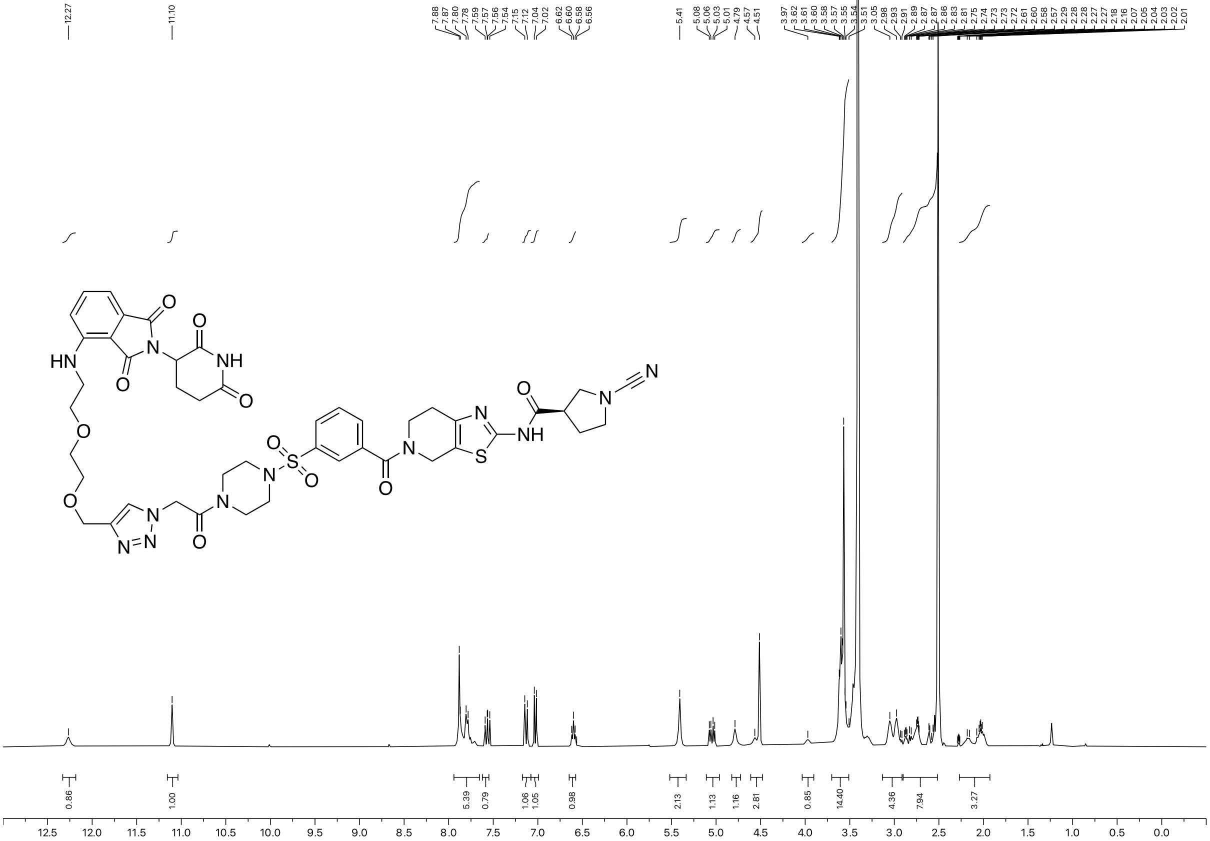

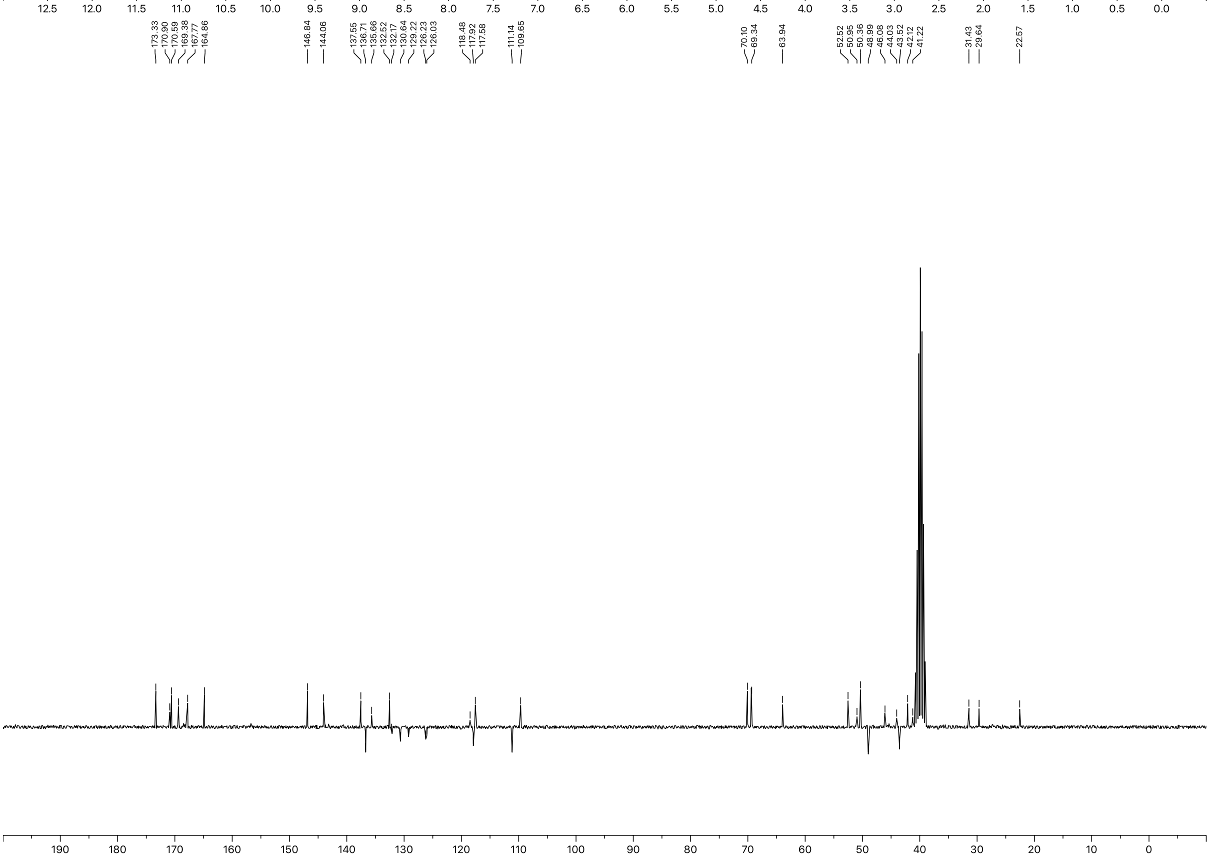

^{1}H-NMR and ^{13}C-NMR of compound JYQ-188 in DMSO.

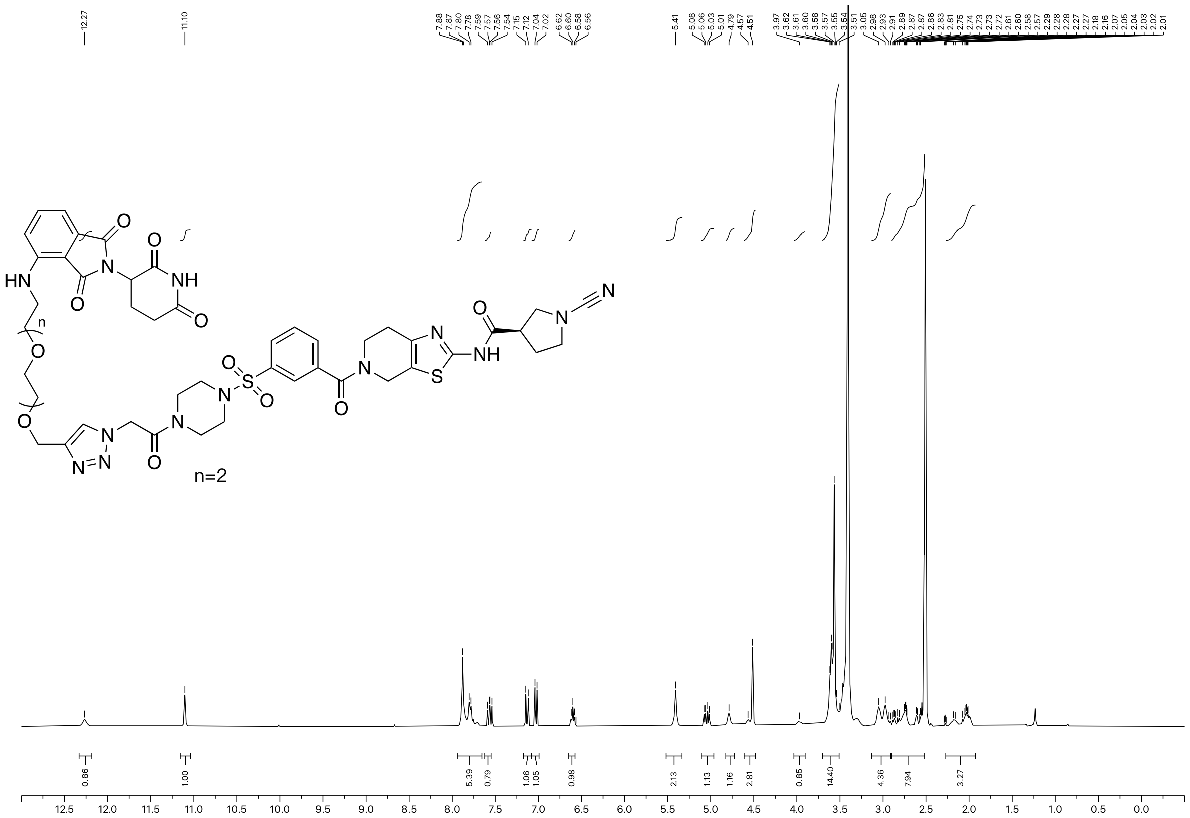

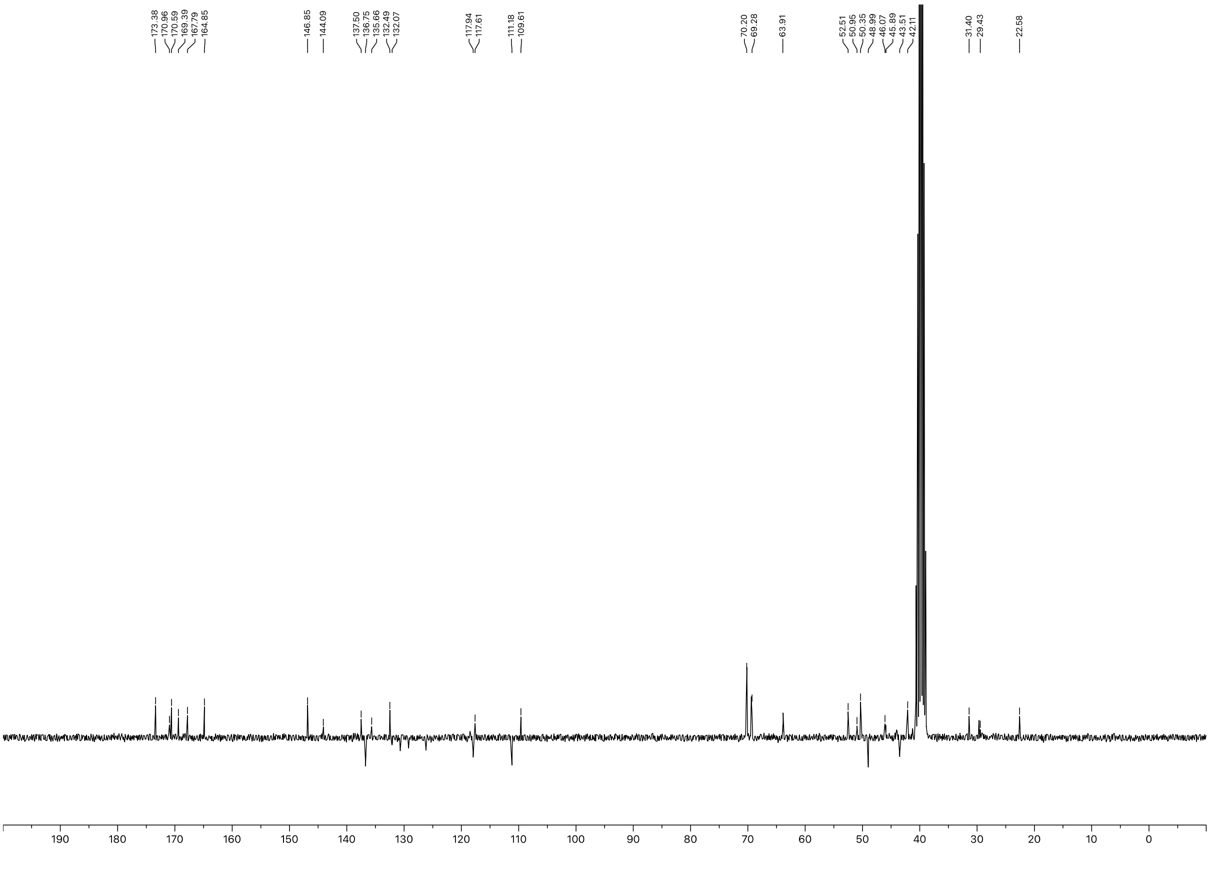

^{1}H-NMR of compound 480 in DMSO.

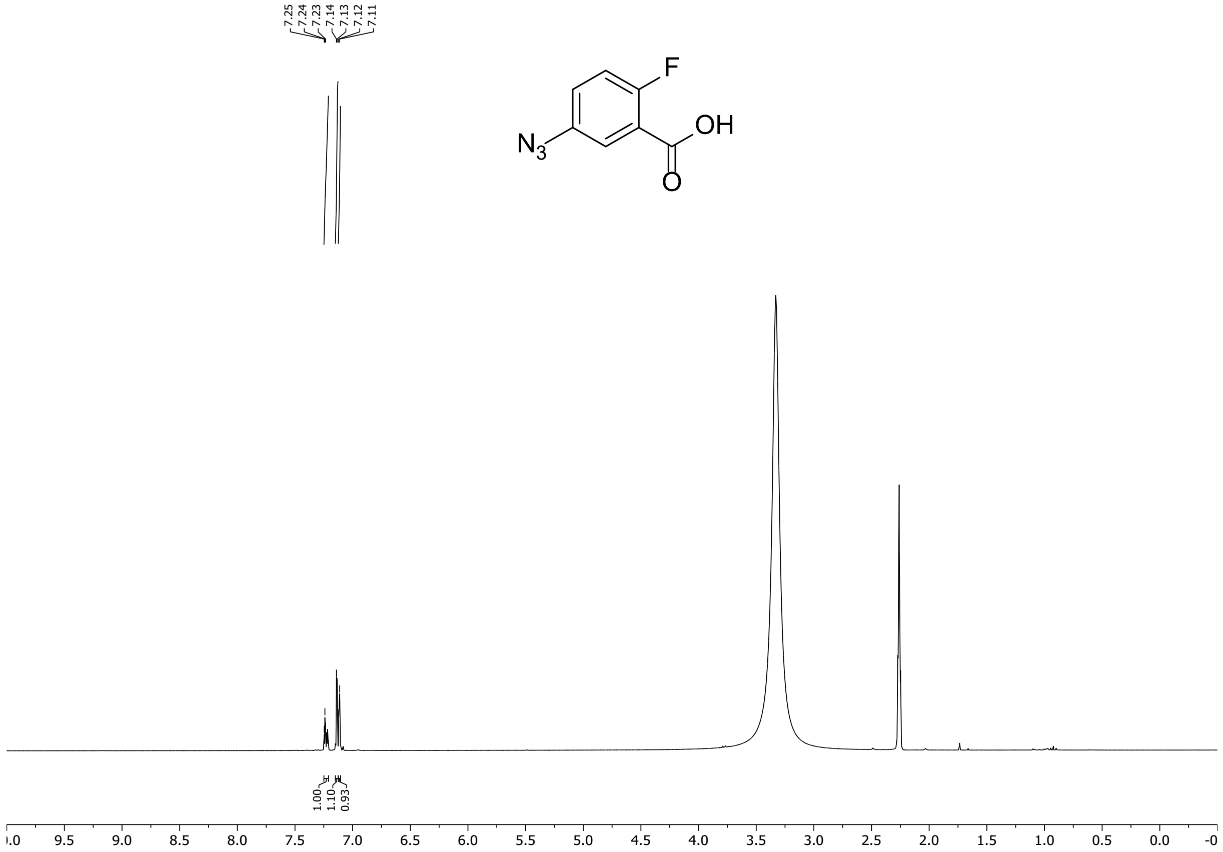

^{1}H-NMR and ^{13}C-NMR of compound 481 in DMSO.

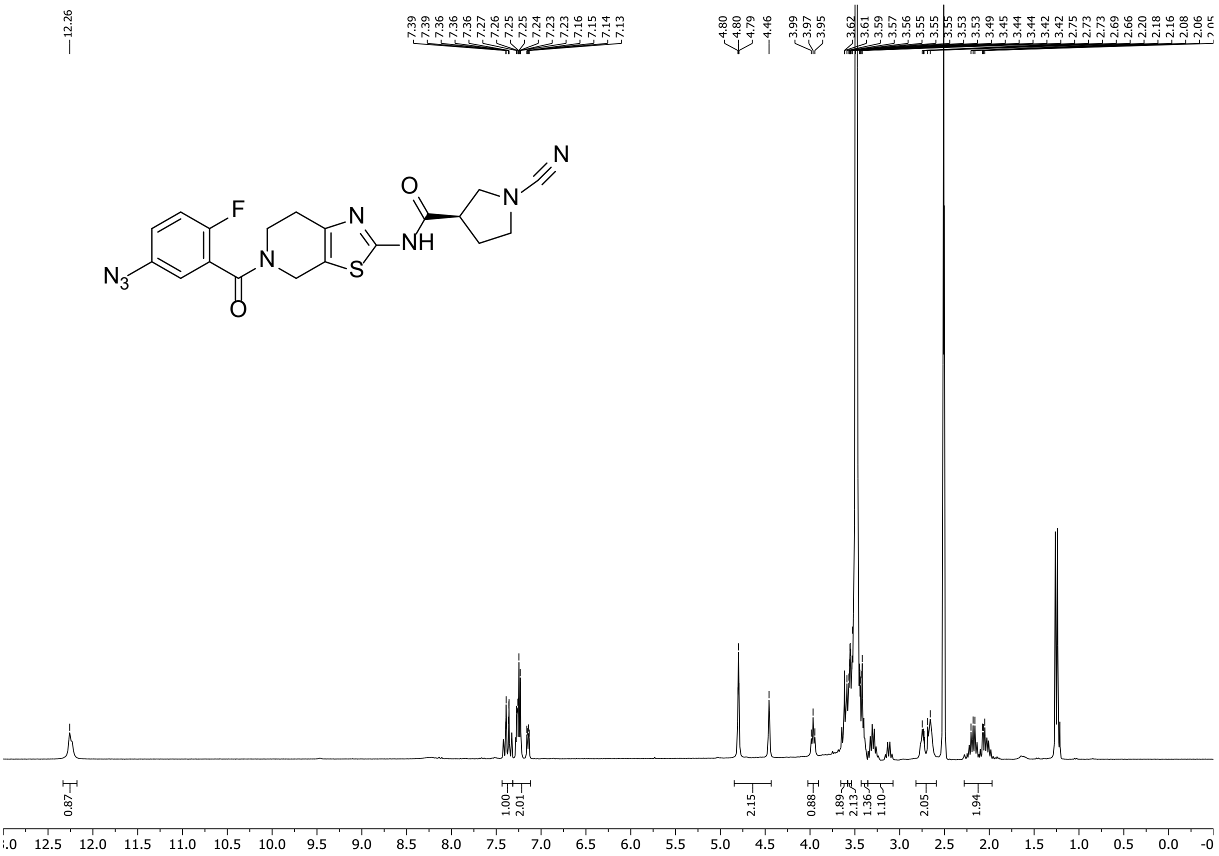

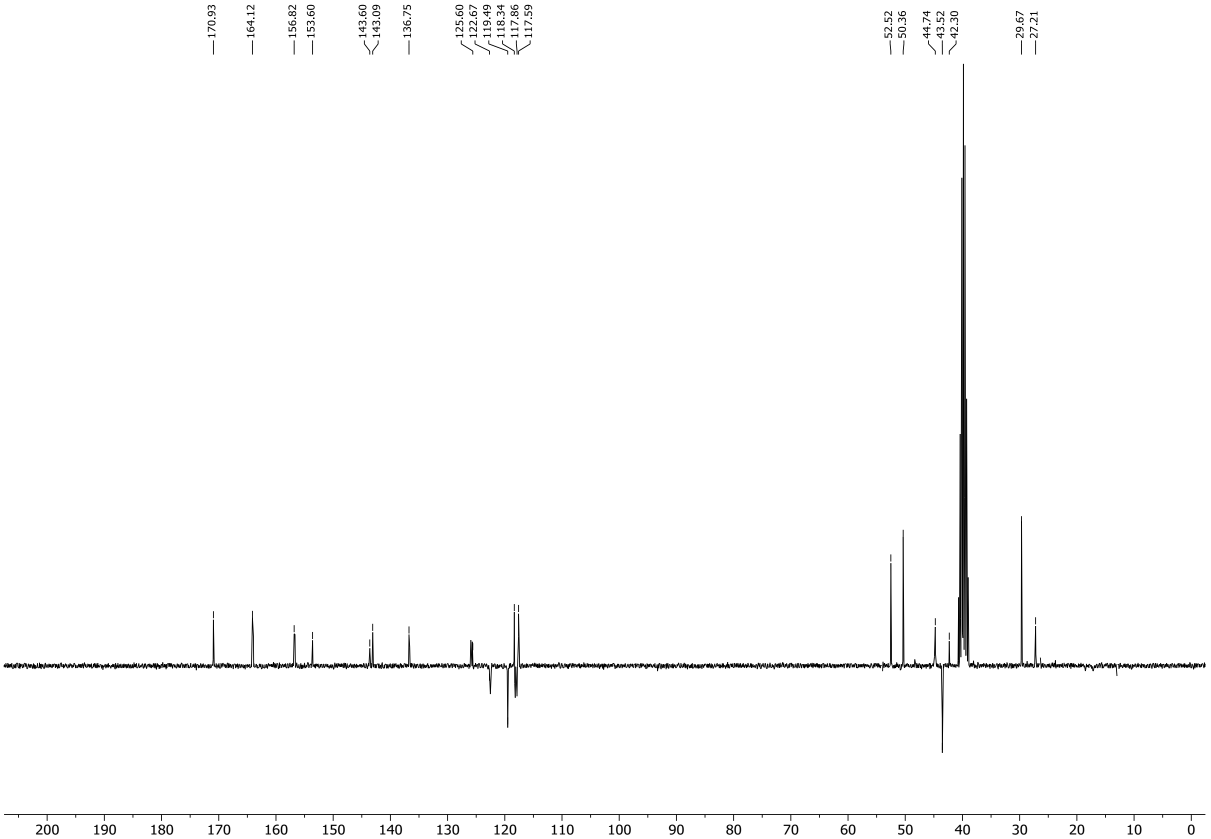

^{1}H-NMR and ^{13}C-NMR of compound JYQ-191 in DMSO.

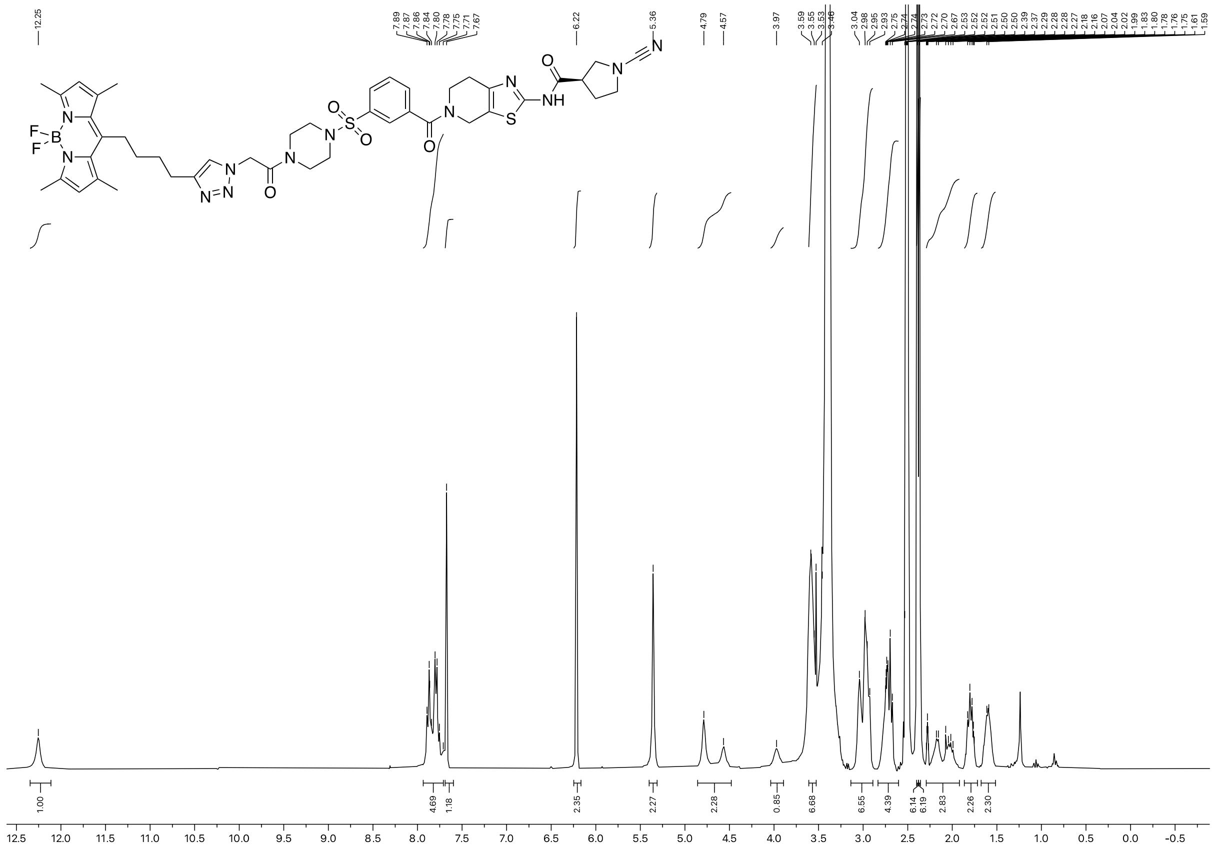

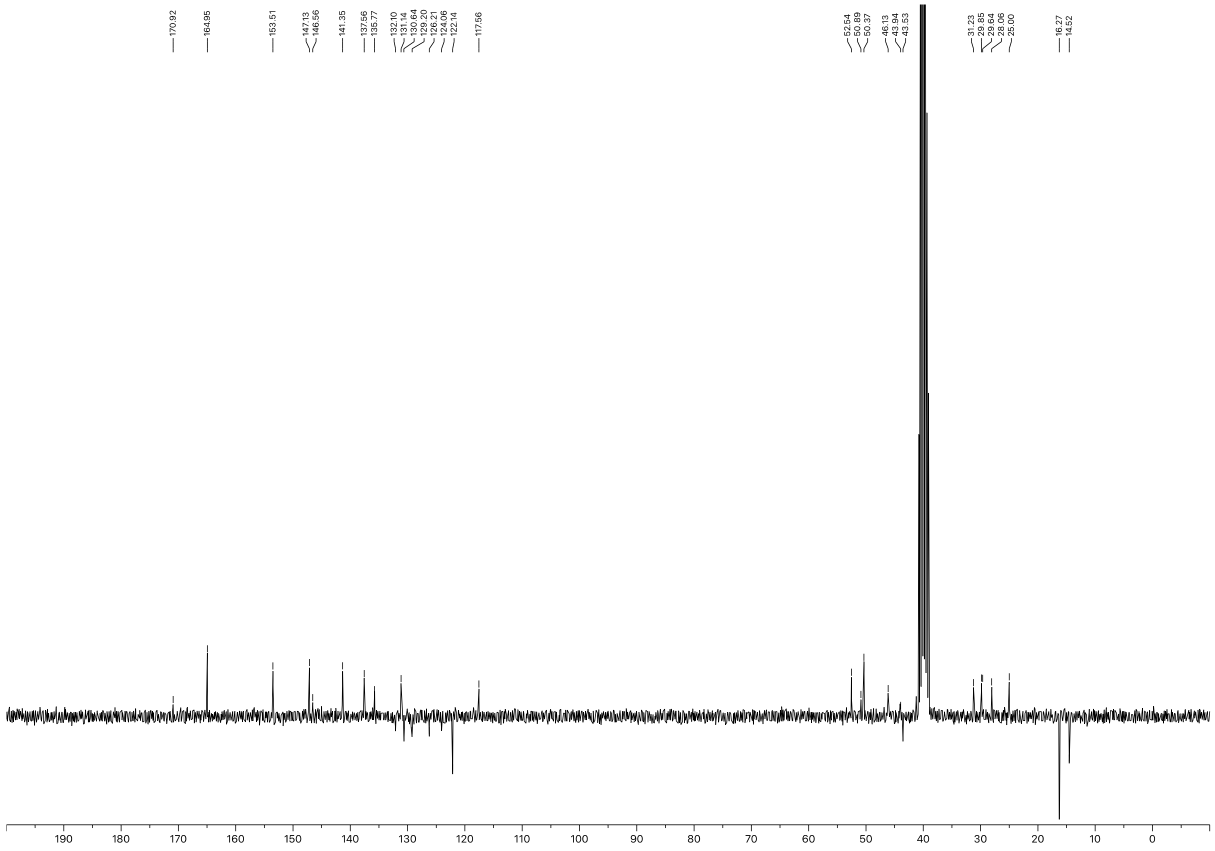

^{1}H-NMR and ^{13}C-NMR of compound JYQ-192 in DMSO.

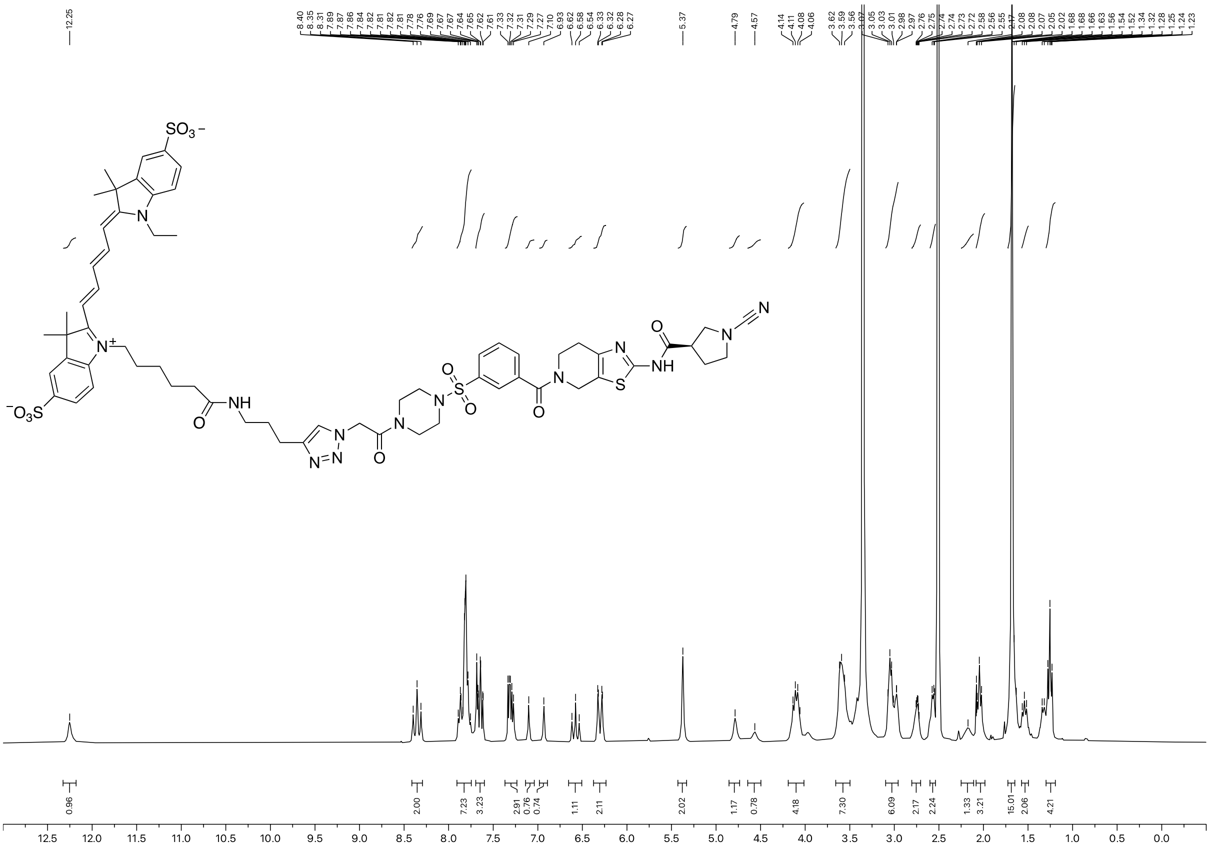

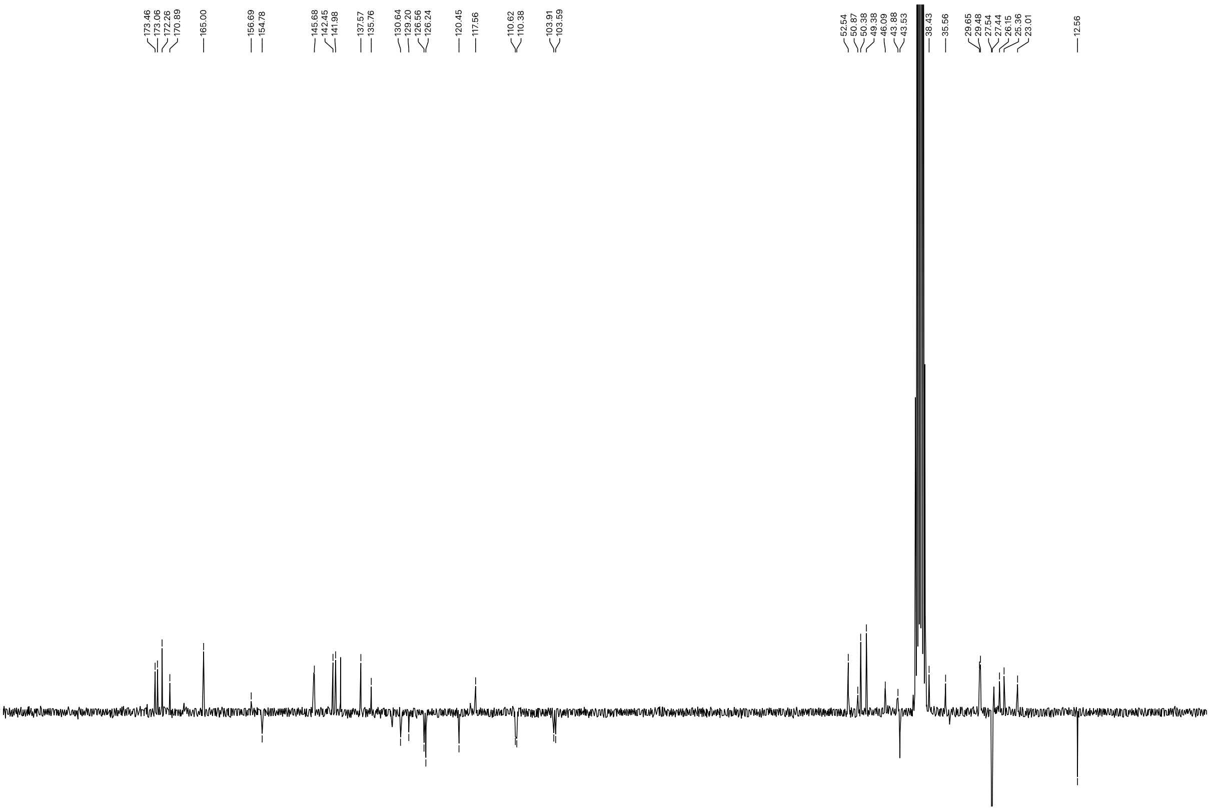

^{1}H-NMR and ^{13}C-NMR of compound JYQ-194 in DMSO.

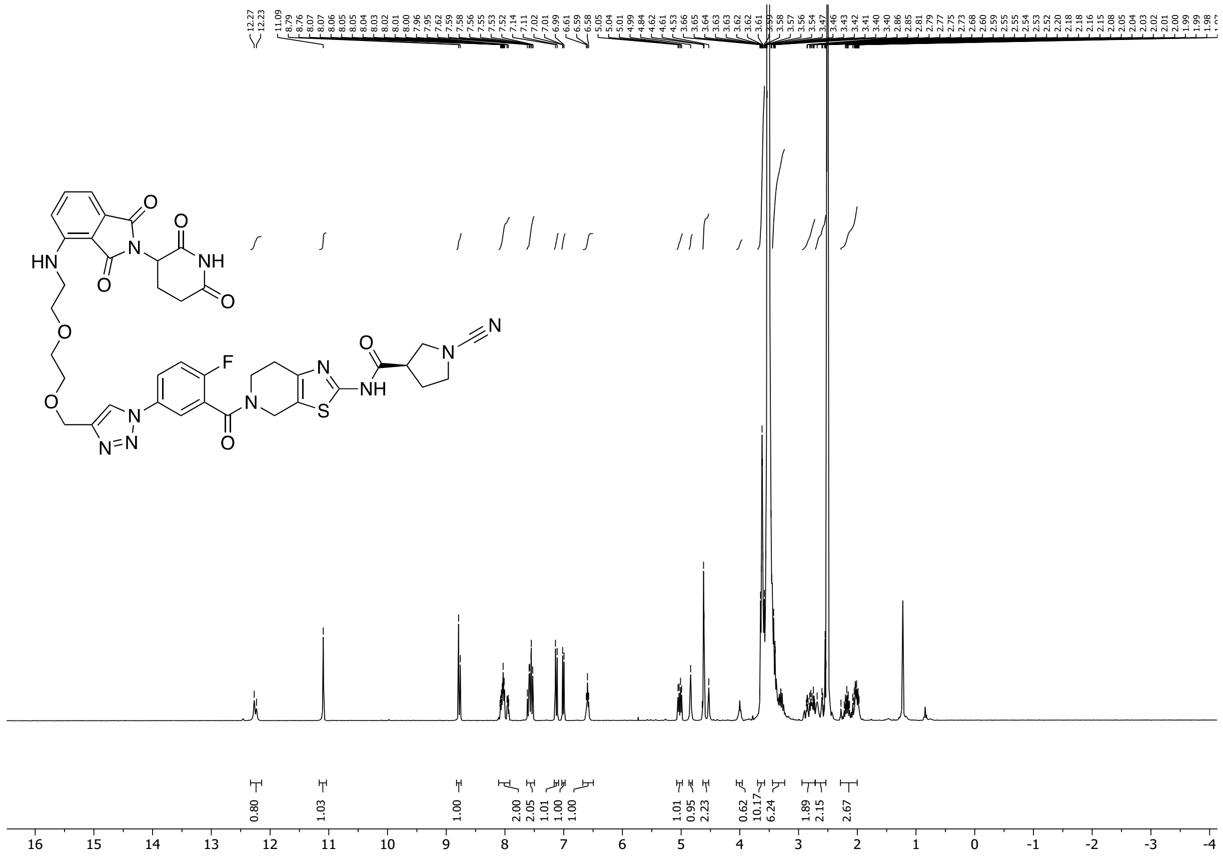

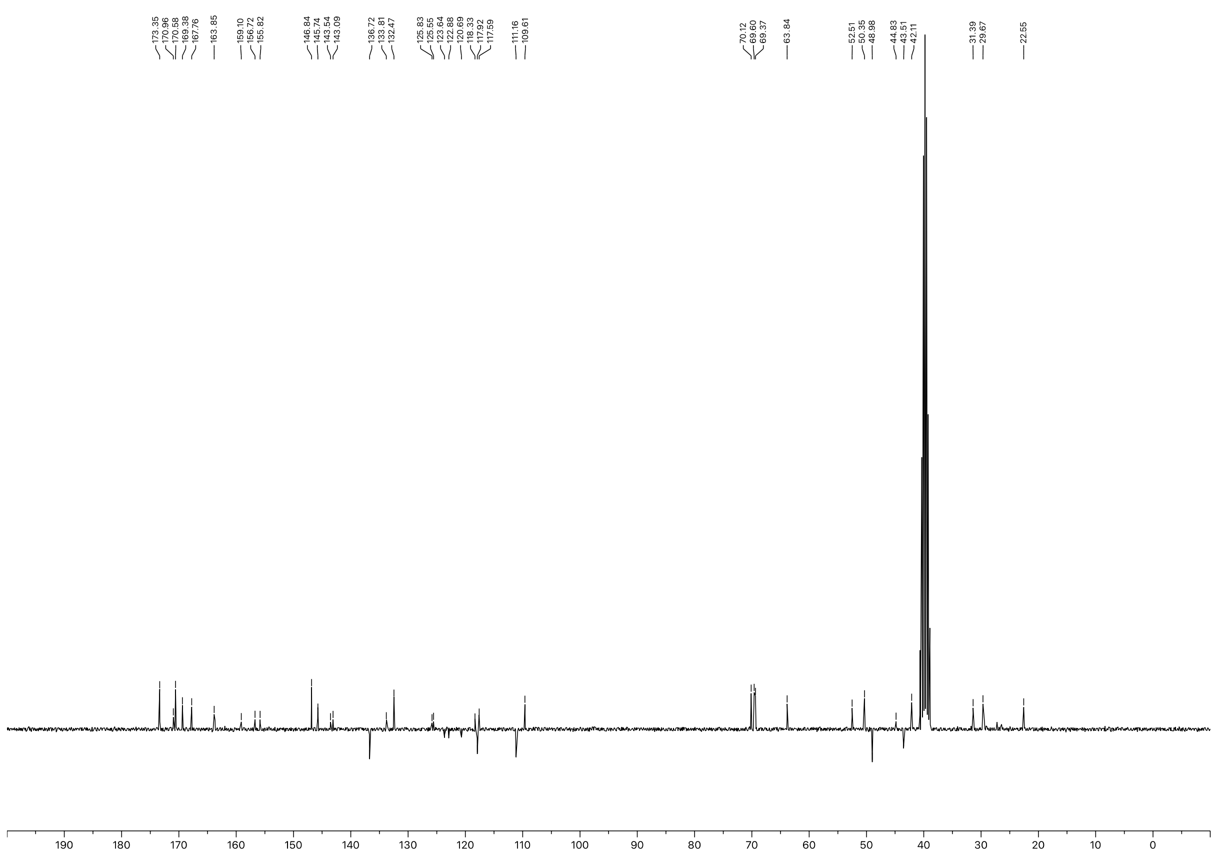

^{1}H-NMR and ^{13}C-NMR of compound JYQ-195 in DMSO.

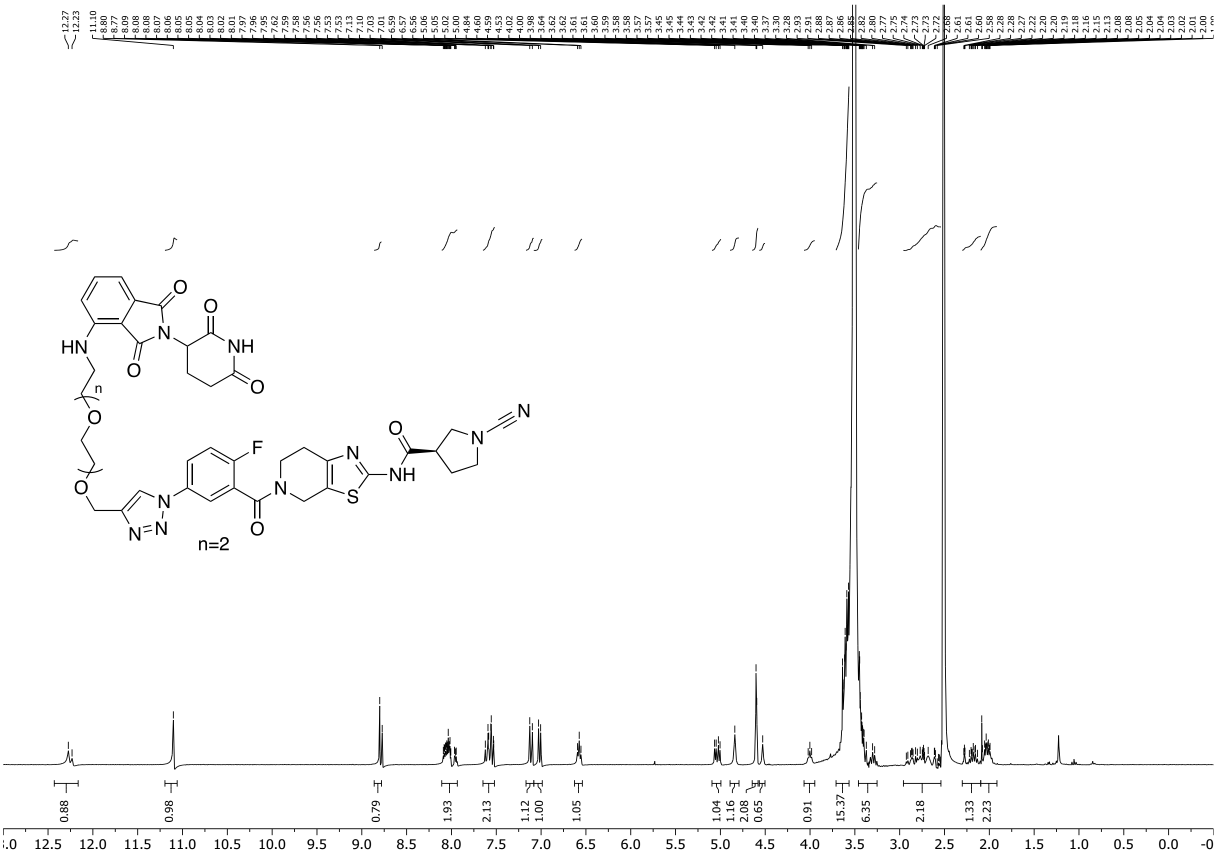

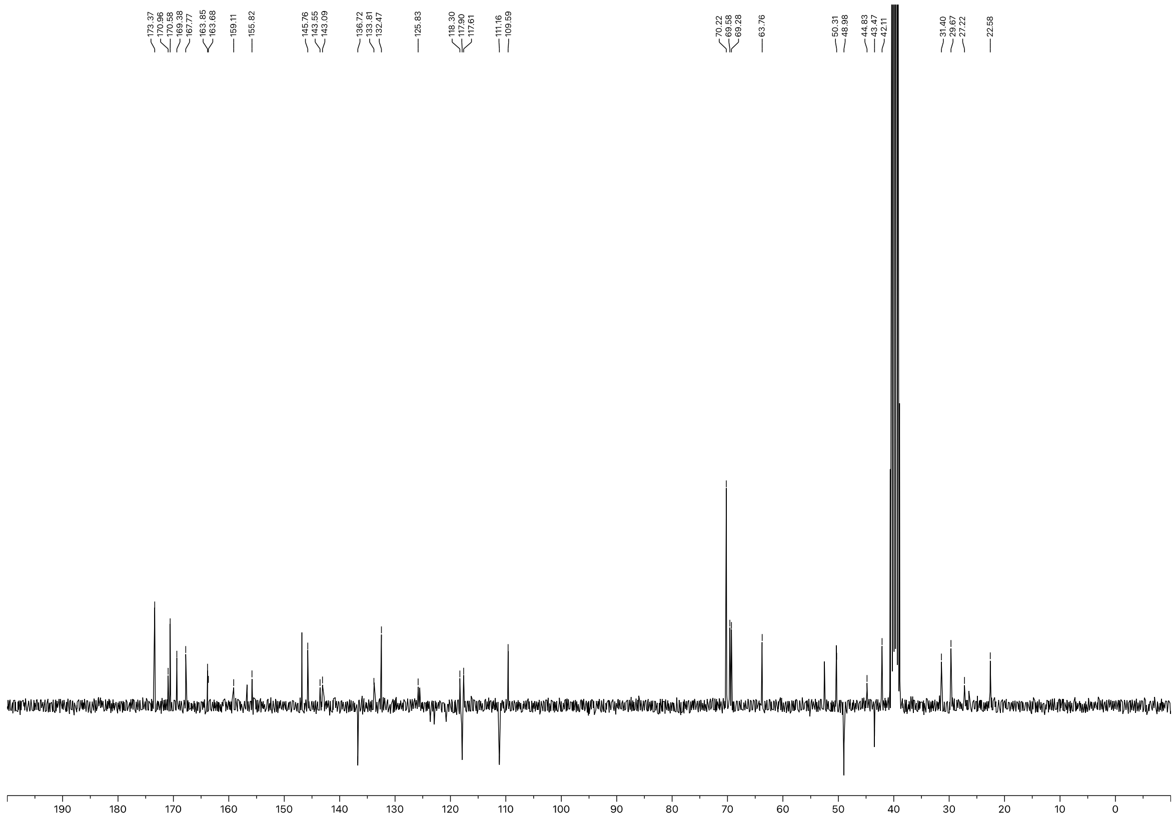

1H-NMR and 13C-NMR of compound JYQ-196 in DMSO.

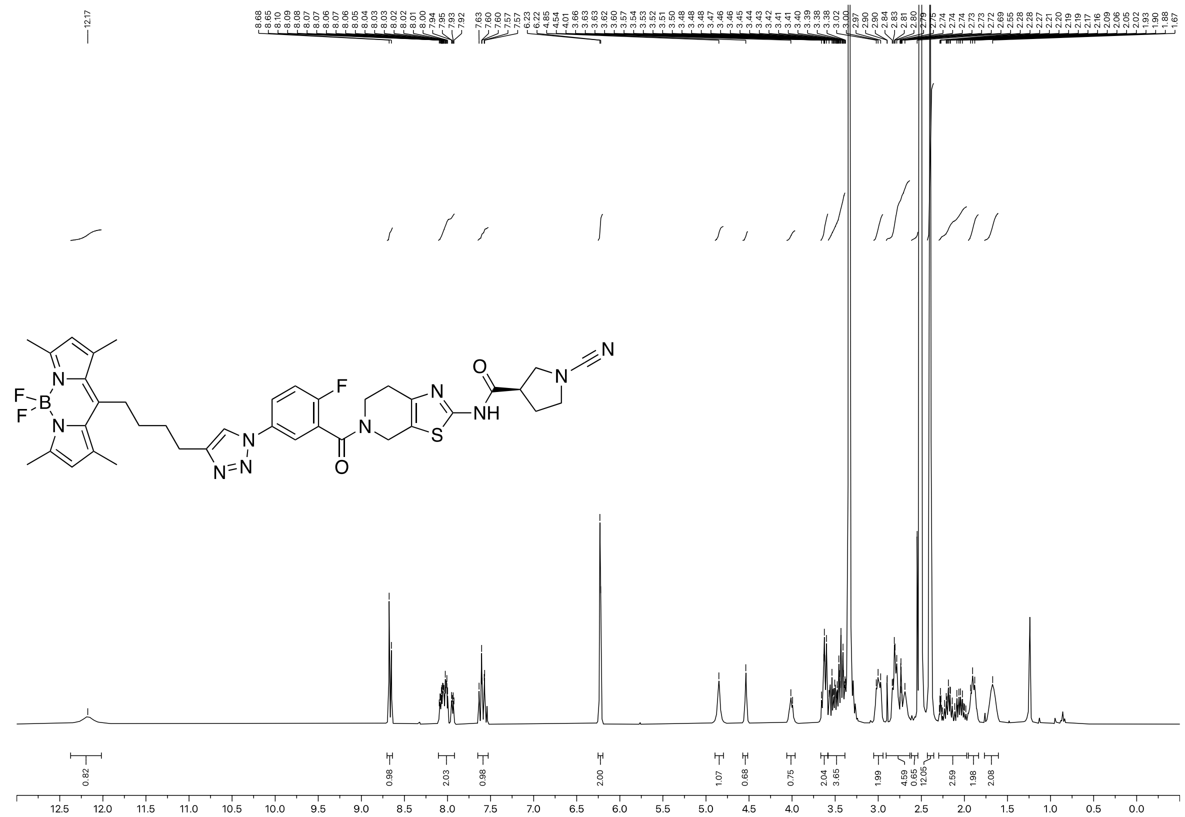

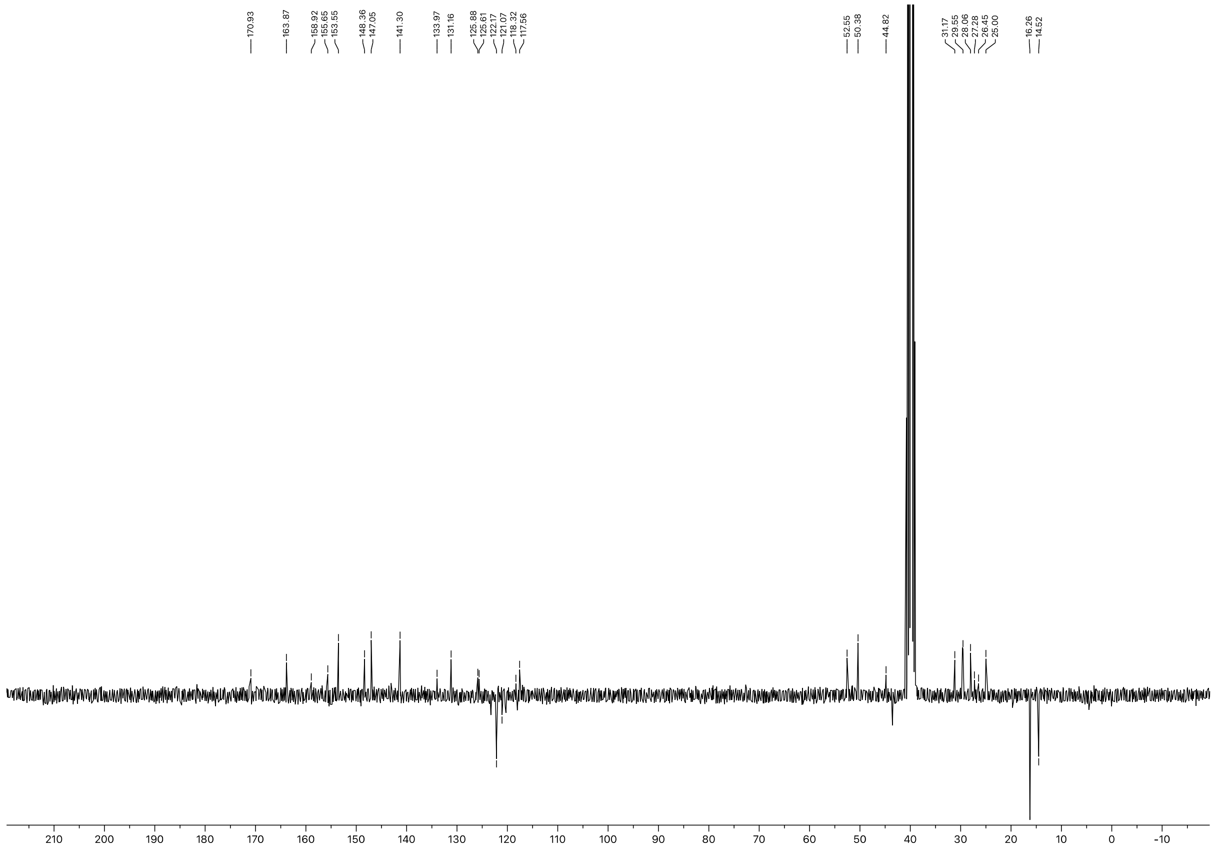

^{1}H-NMR and ^{13}C-NMR of compound JYQ-197 in DMSO.

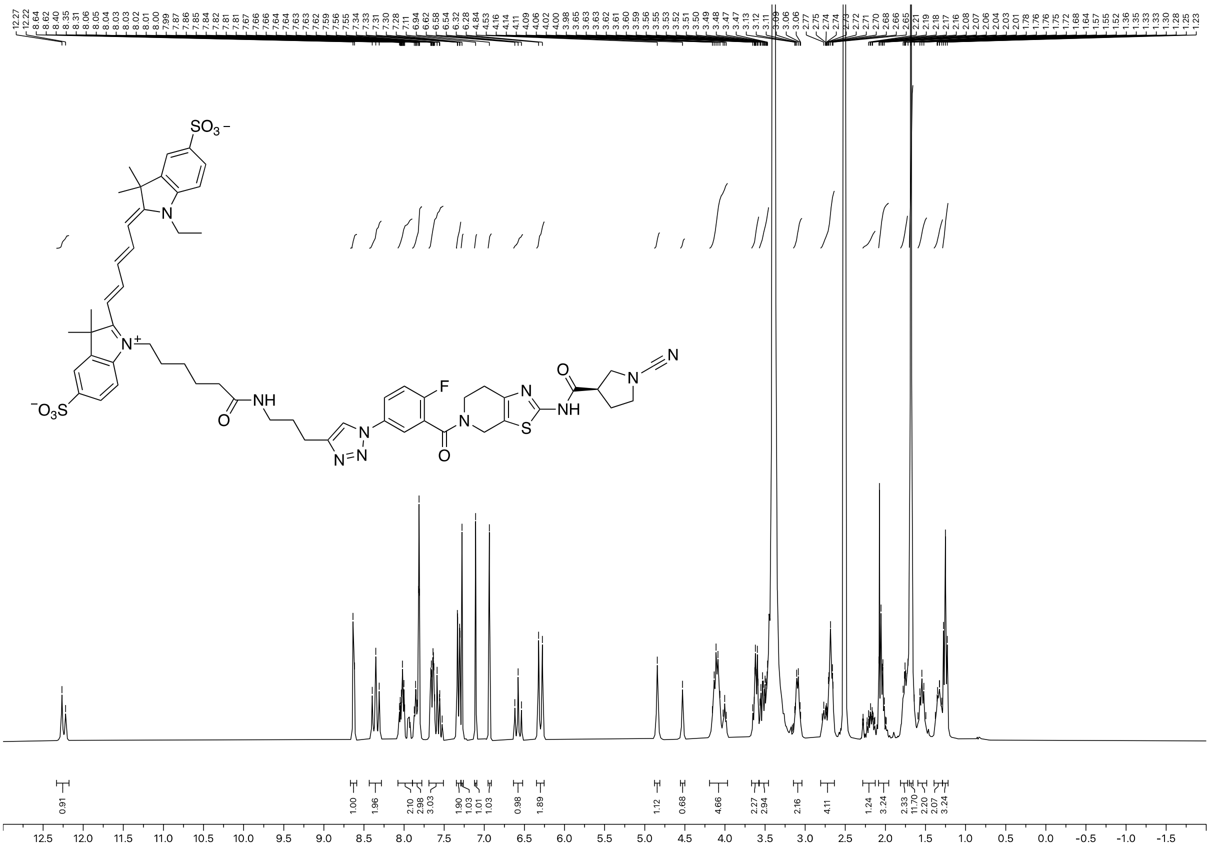

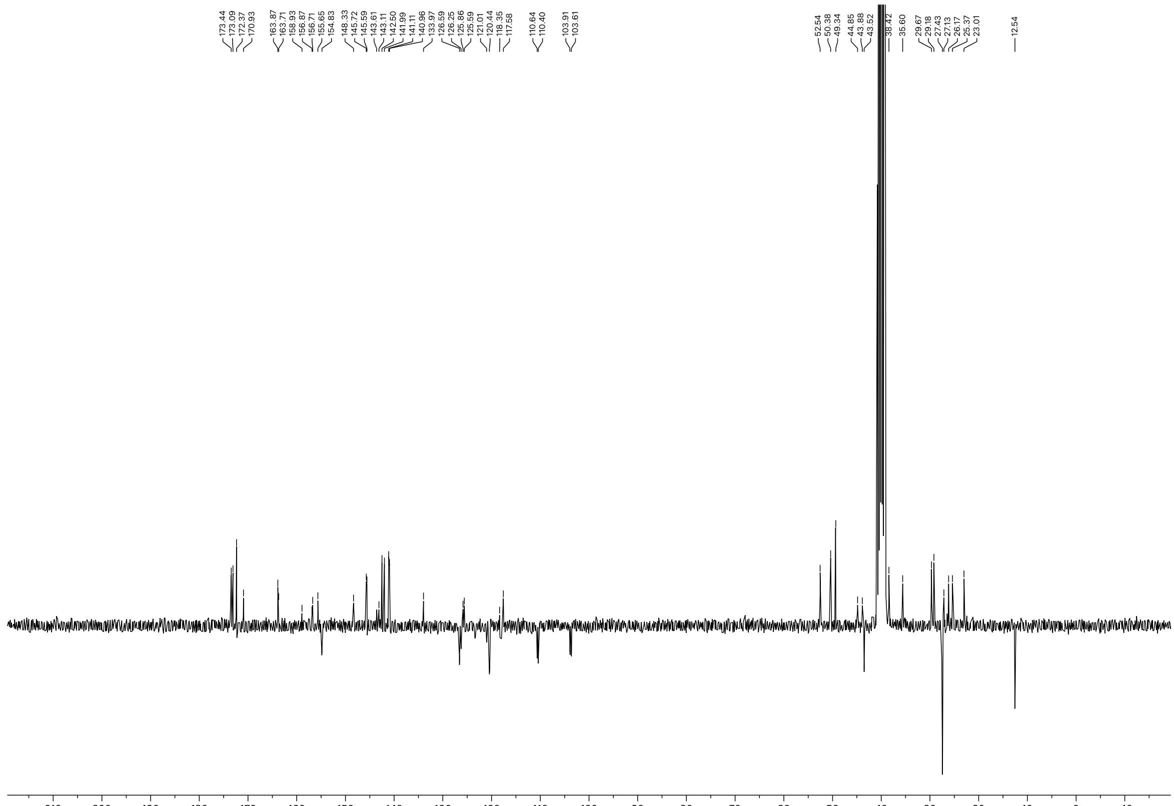

JYQ-164

Time	Height	Area	Area%
3.42	14763	142.17	0.05
3.64	12895986	276325.06	99.95

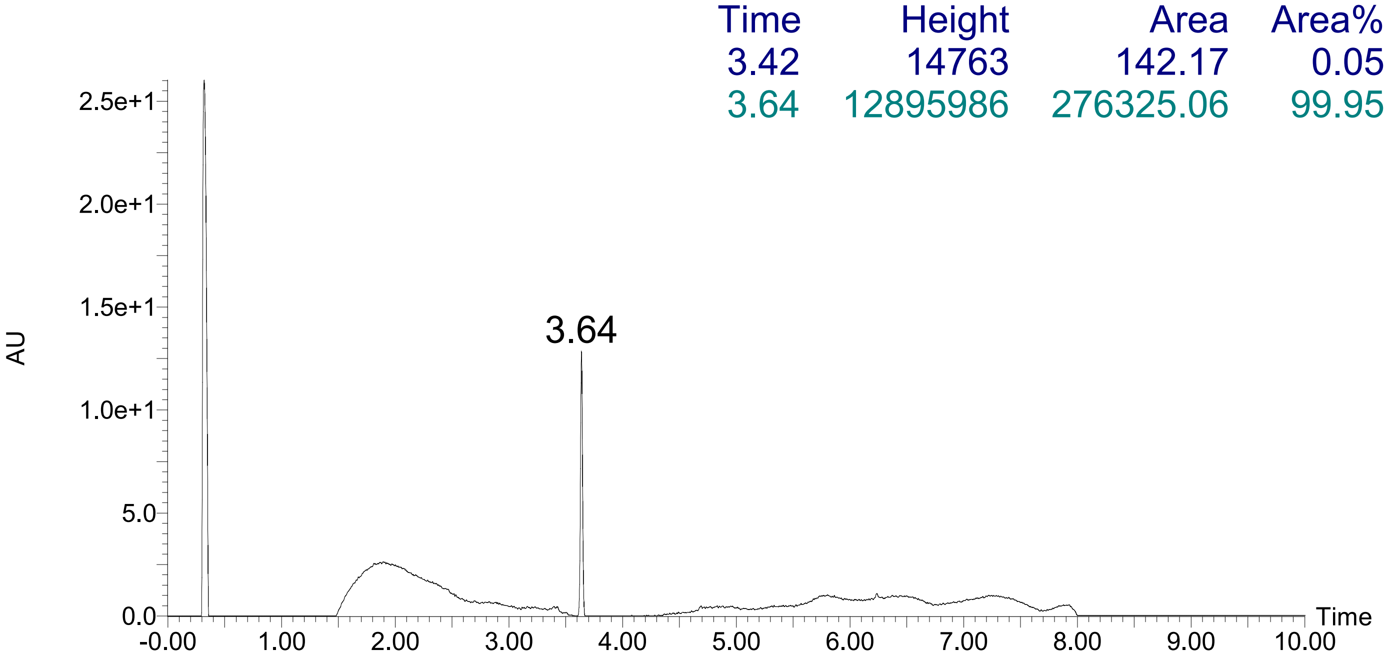

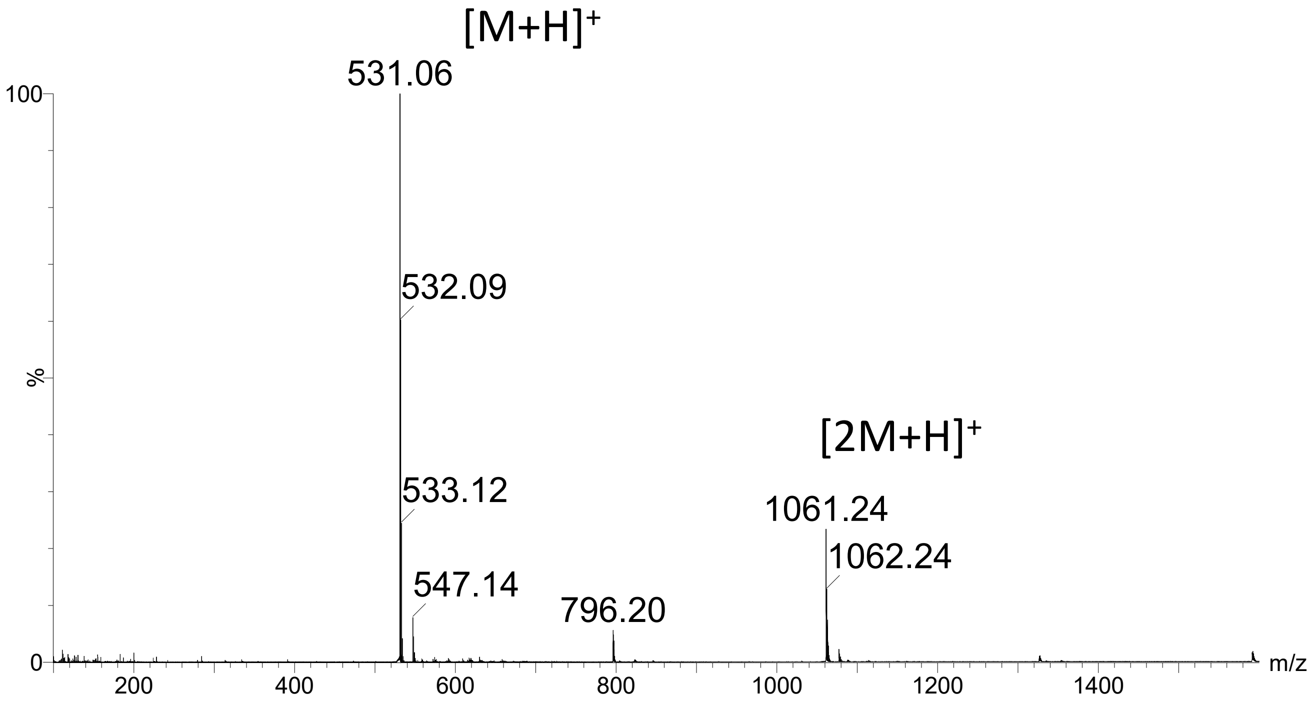

JYQ-173

Time	Height	Area	Area%
3.08	264266	3514.35	0.69
3.45	24131220	502822.38	99.31

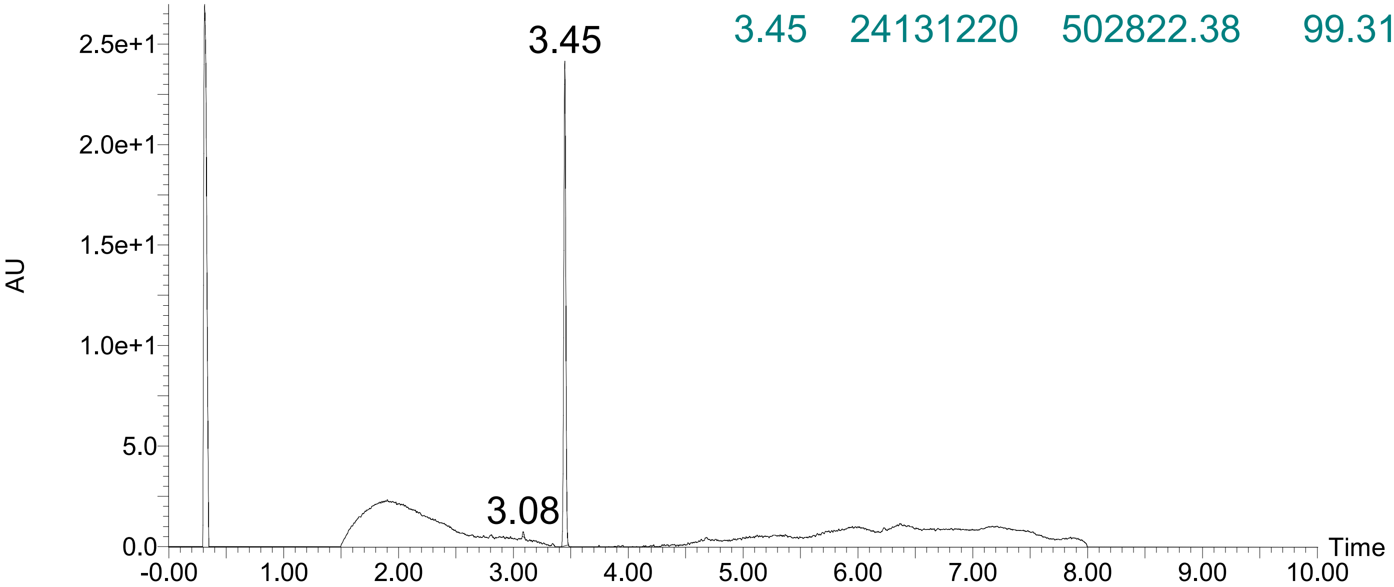

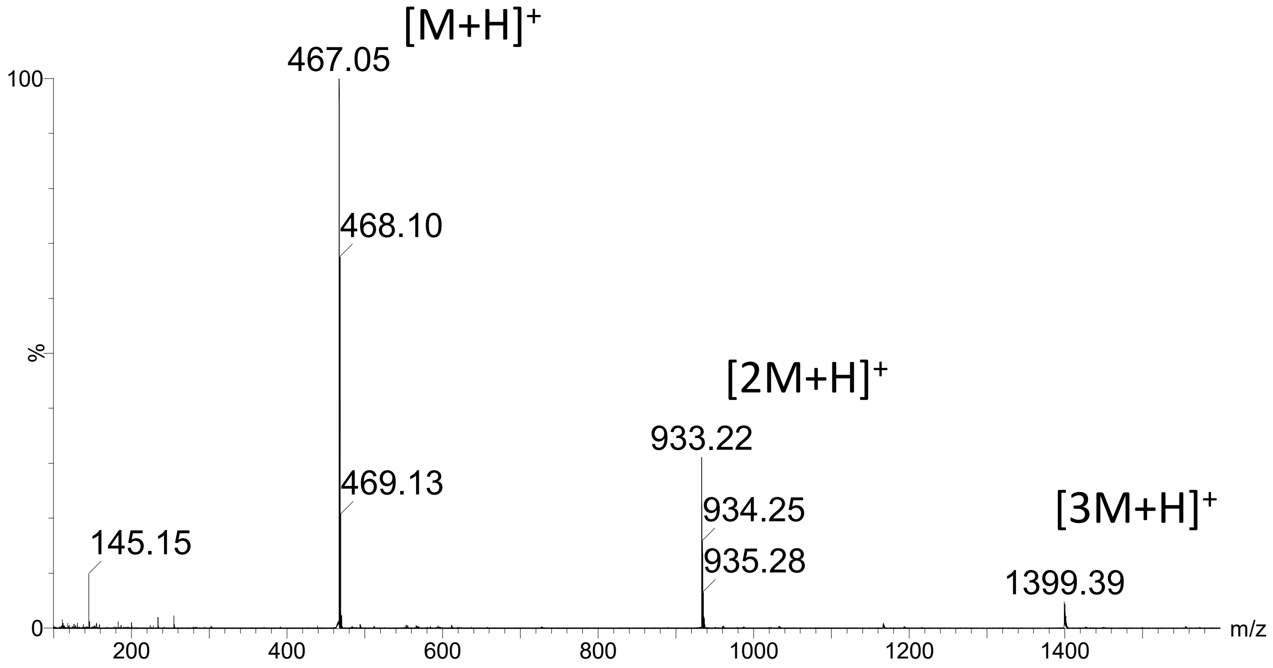

JYQ-191

Time	Height	Area	Area%
4.99	210089	3273.53	0.60
5.20	24621370	538179.19	99.40

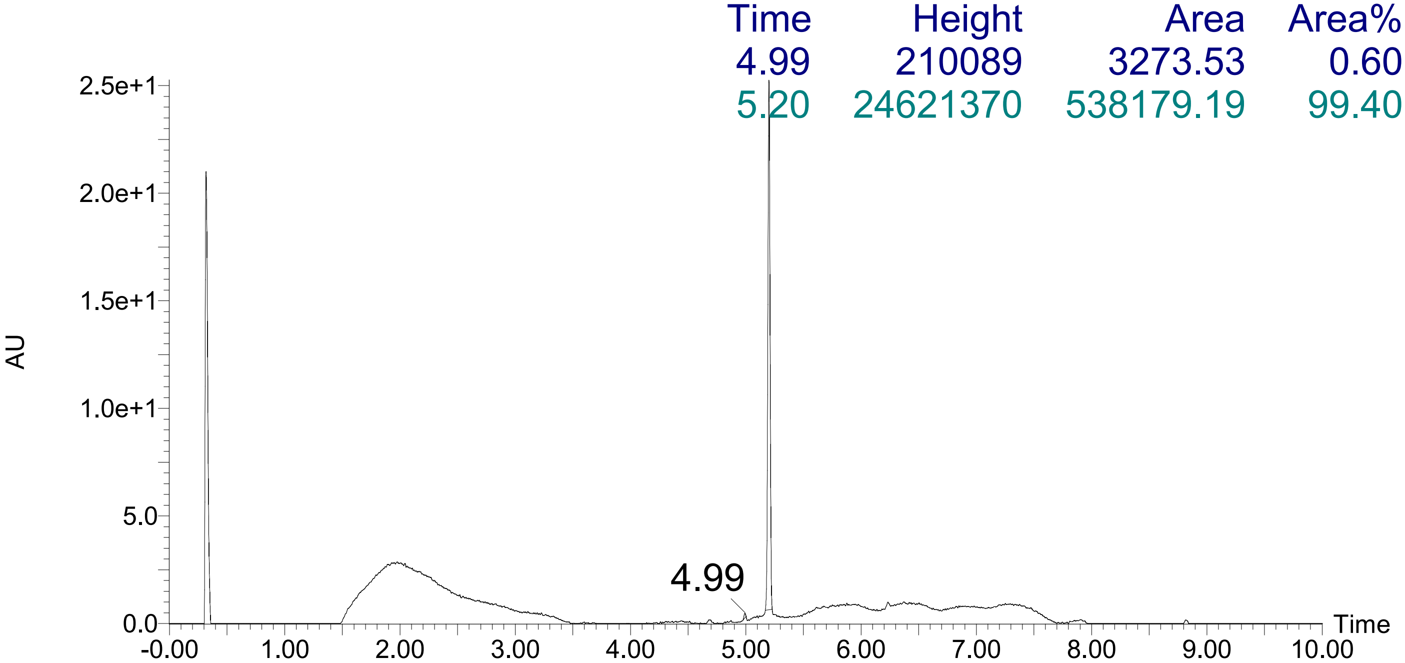

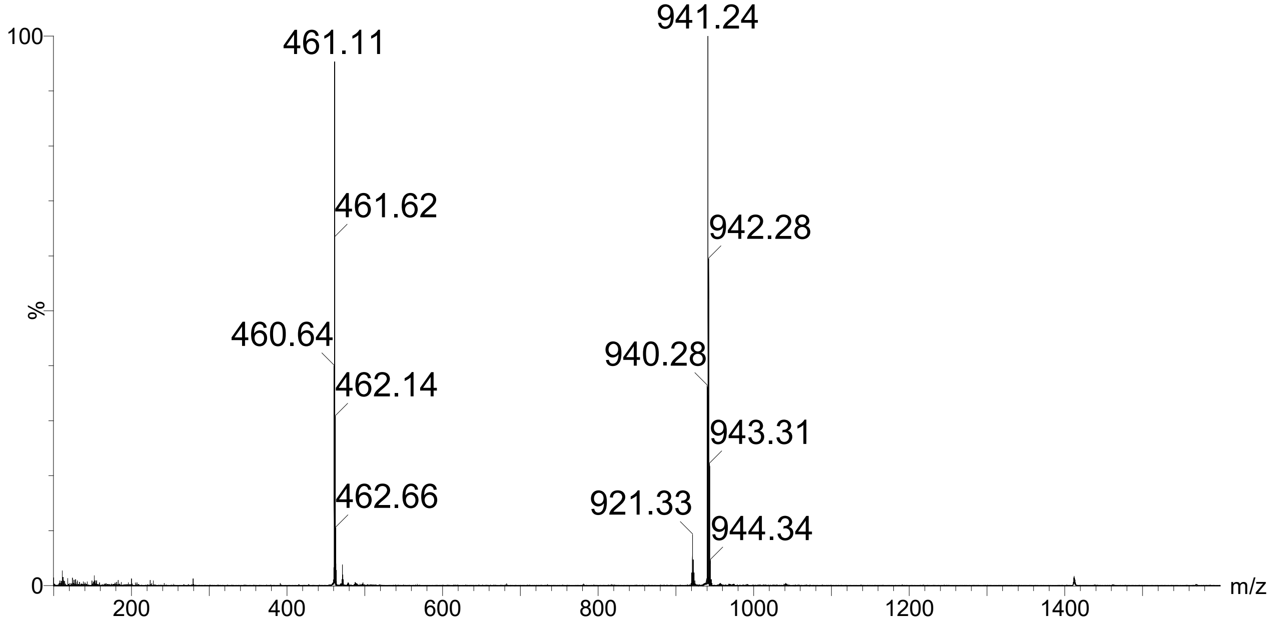

JYQ-192

Time	Height	Area	Area%
3.50	2158610	68878.09	1.99
3.70	94840592	3394834.25	98.01

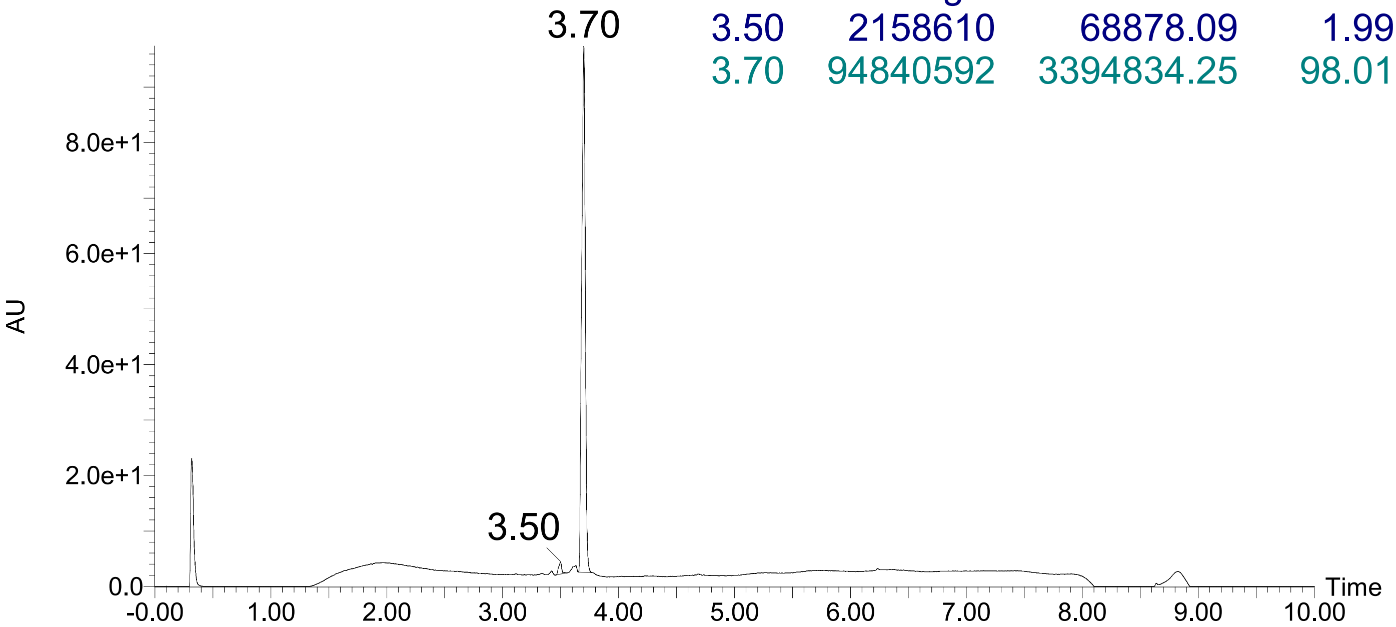

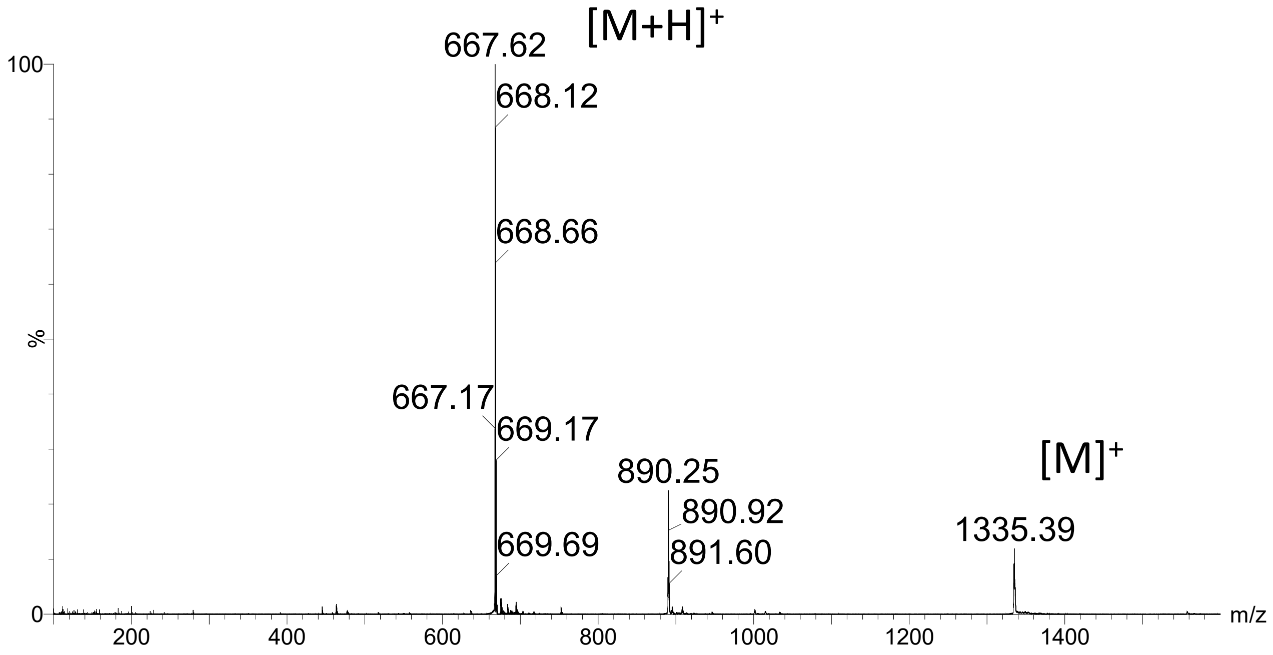

JYQ-196

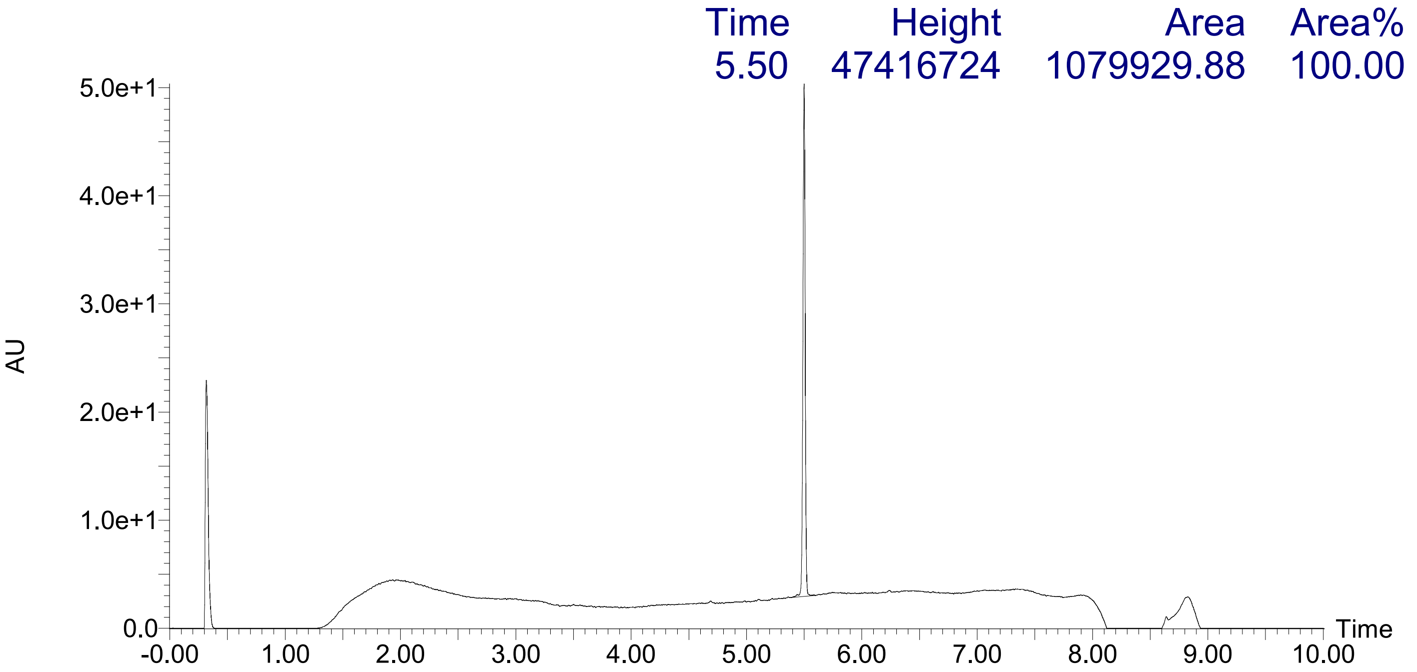

Time Height Area Area%
5.50 47416724 1079929.88 100.00
5.0e+1
4.0e+1
3.0e+1
2.0e+1
1.0e+1
0.0
AU
-0.00
1.00
2.00
3.00
4.00
5.00
6.00
7.00
8.00
9.00
10.00
Time

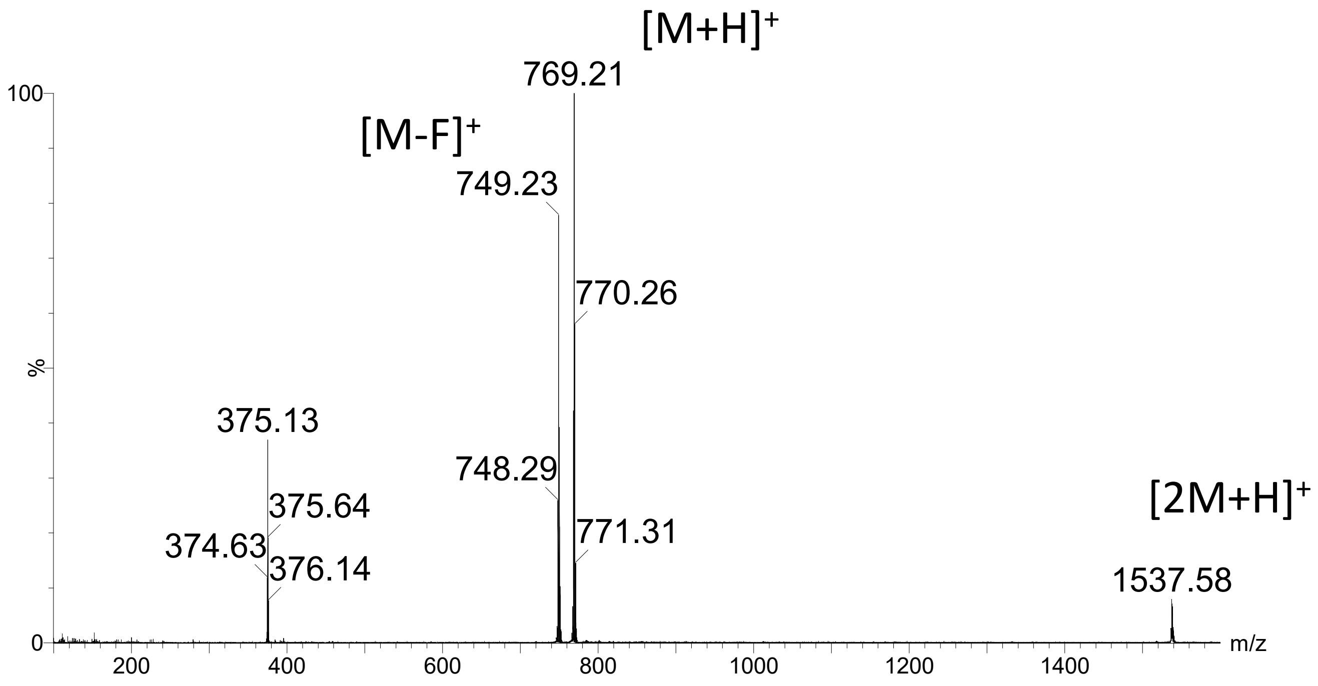

[M+H]+
769.21
[M-F]+
749.23
770.26
375.13
748.29
375.64
374.63
376.14
771.31
[2M+H]+
1537.58
100
%
0
200
400
600
800
1000
1200
1400
m/z

JYQ-197

Time	Height	Area	Area%
3.52	8077578	238015.20	22.87
3.75	24028996	802654.13	77.13

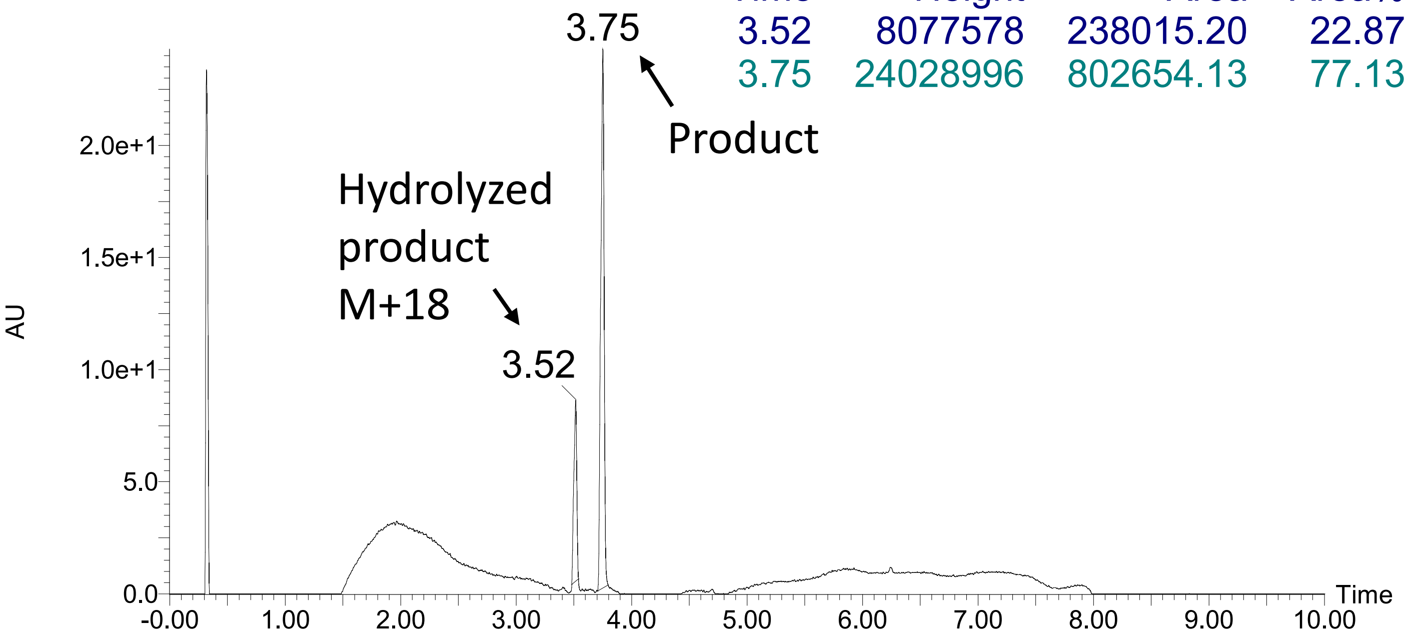

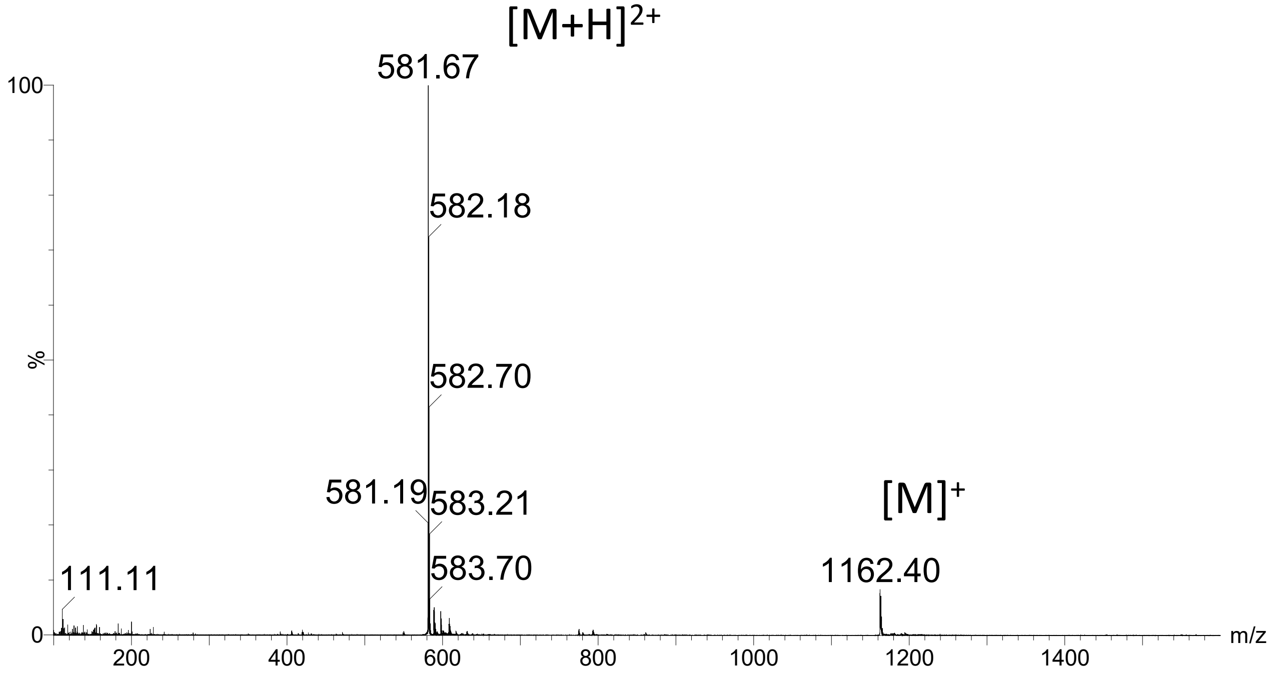

JYQ-187

Time	Height	Area	Area%
3.71	185865	2974.73	1.31
4.08	10335345	223768.63	98.69

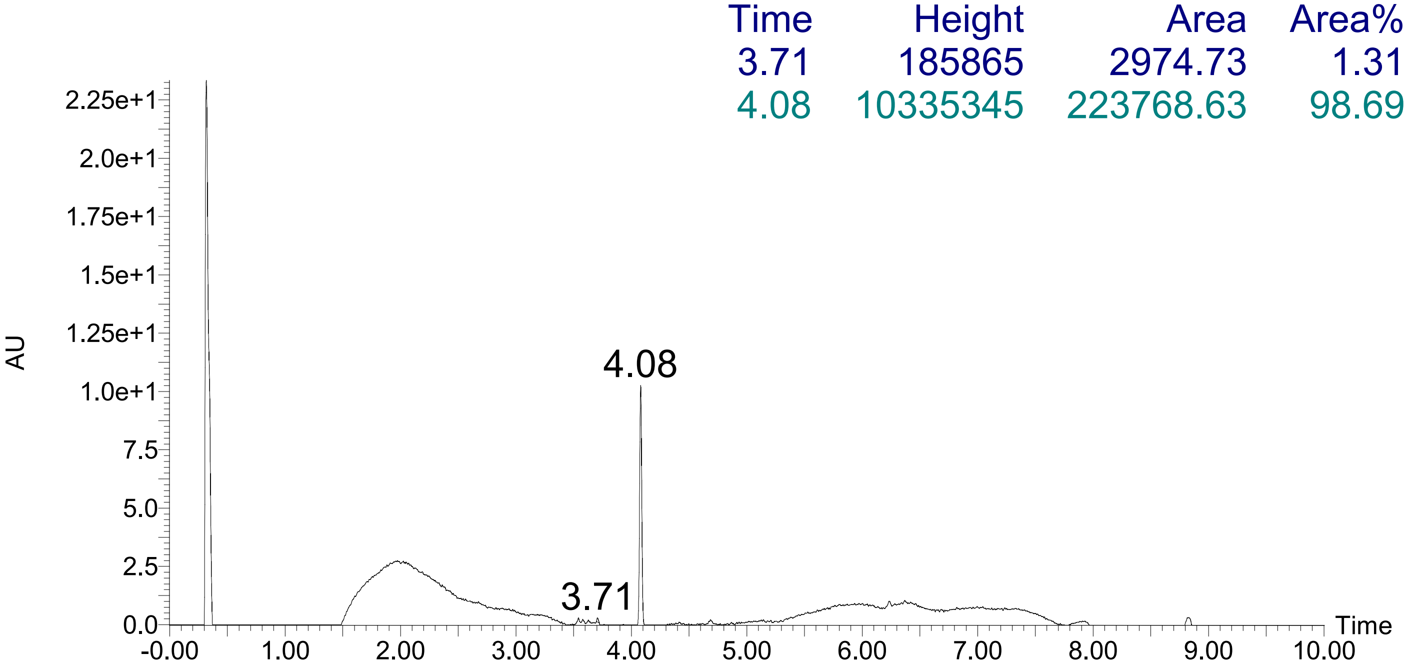

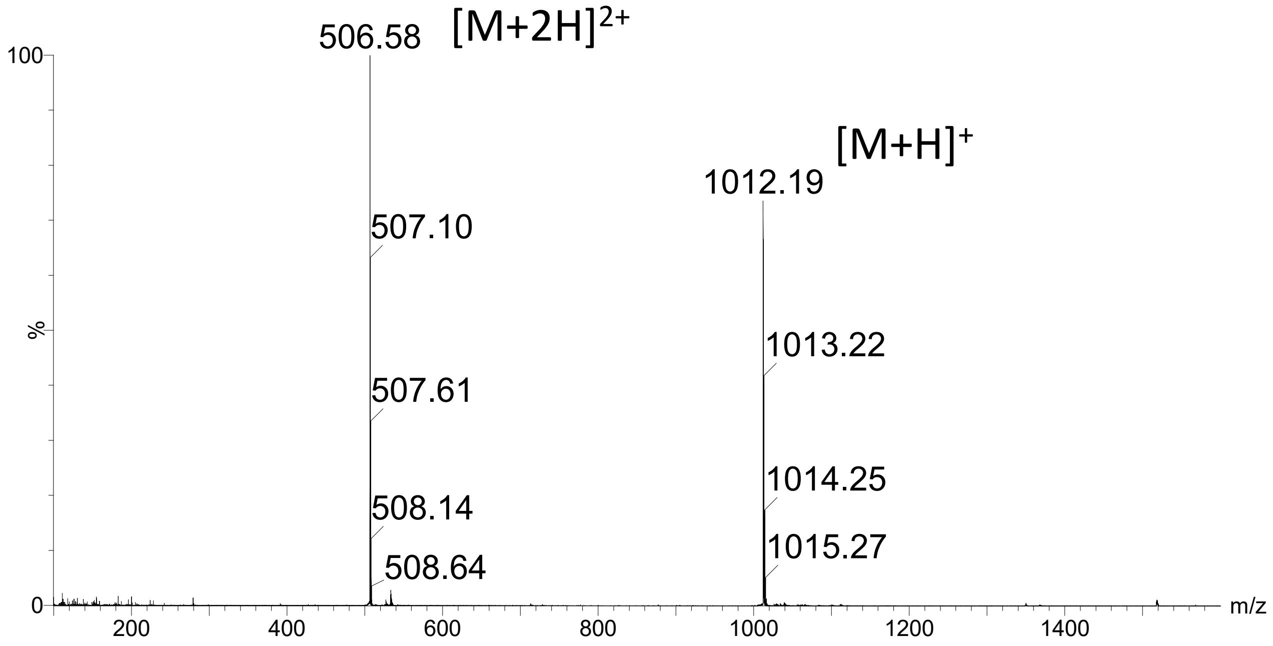

JYQ-188

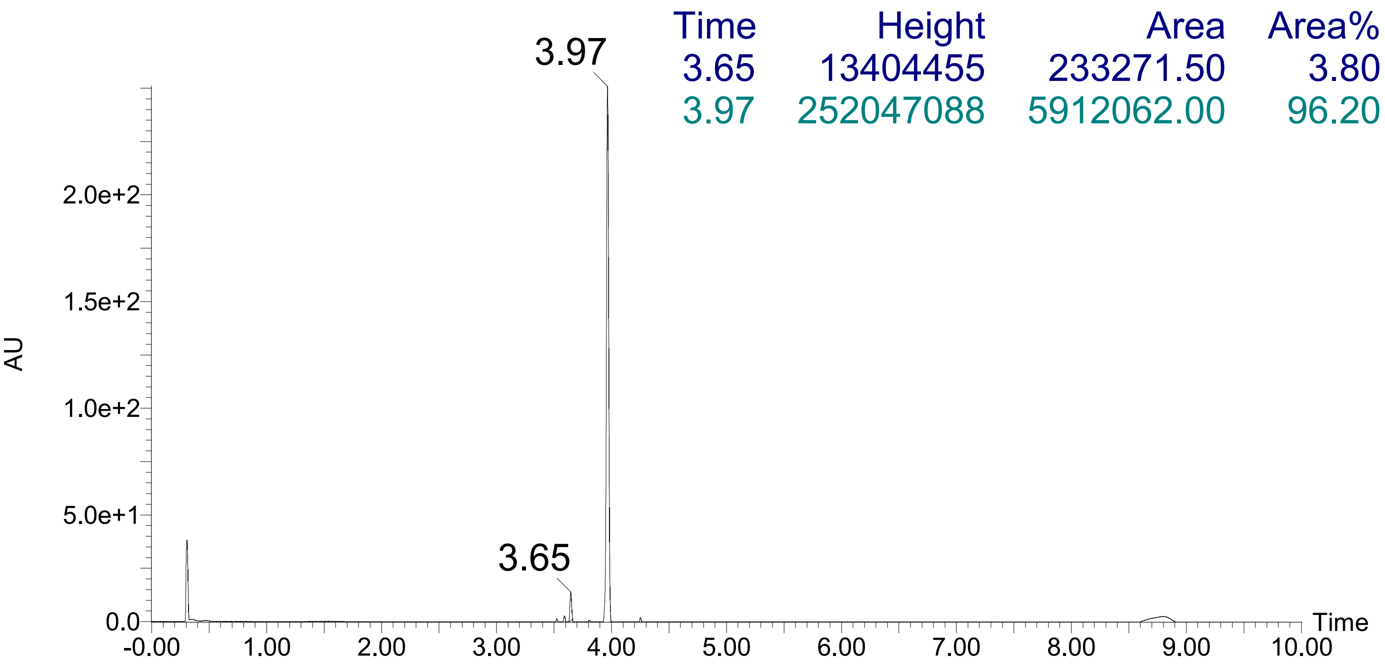

Time Height Area Area%
3.65 13404455 233271.50 3.80
3.97 252047088 5912062.00 96.20
3.97
3.65
AU
2.0e+2
1.5e+2
1.0e+2
5.0e+1
0.0
-0.00
1.00
2.00
3.00
4.00
5.00
6.00
7.00
8.00
9.00
10.00
Time

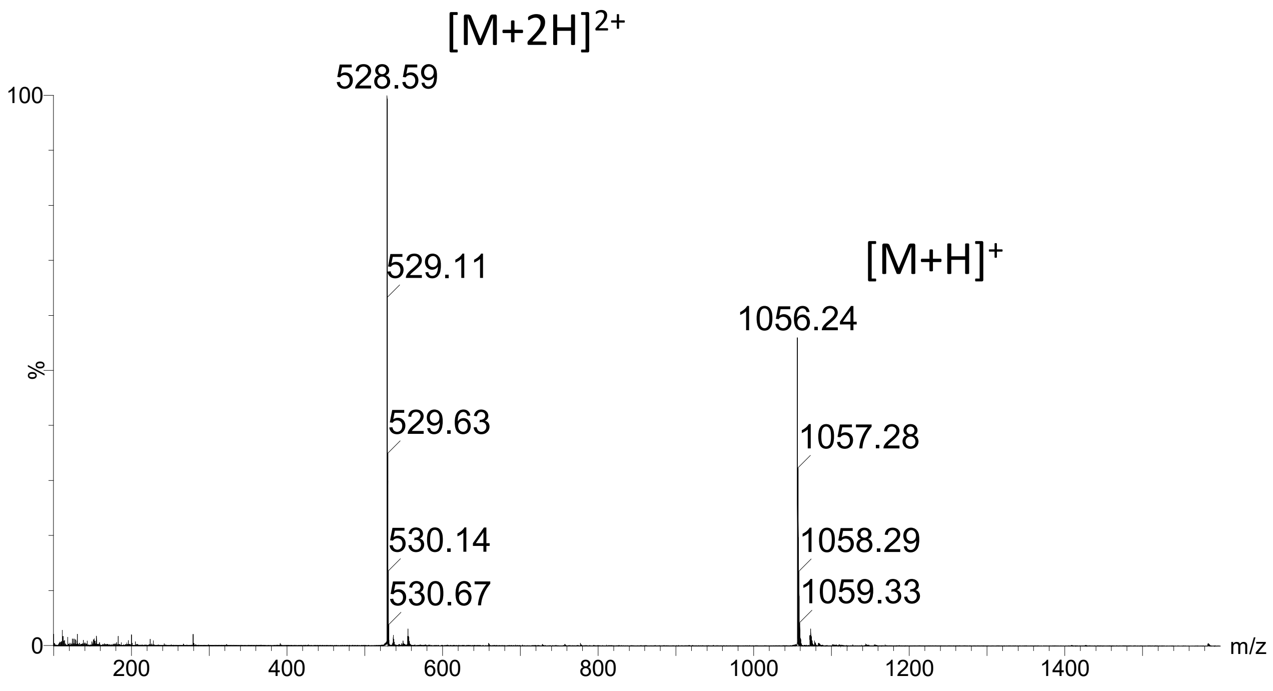

[M+2H]2+
528.59
529.11
529.63
530.14
530.67
[M+H]+
1056.24
1057.28
1058.29
1059.33
100
%
0
200
400
600
800
1000
1200
1400
m/z

JYQ-194

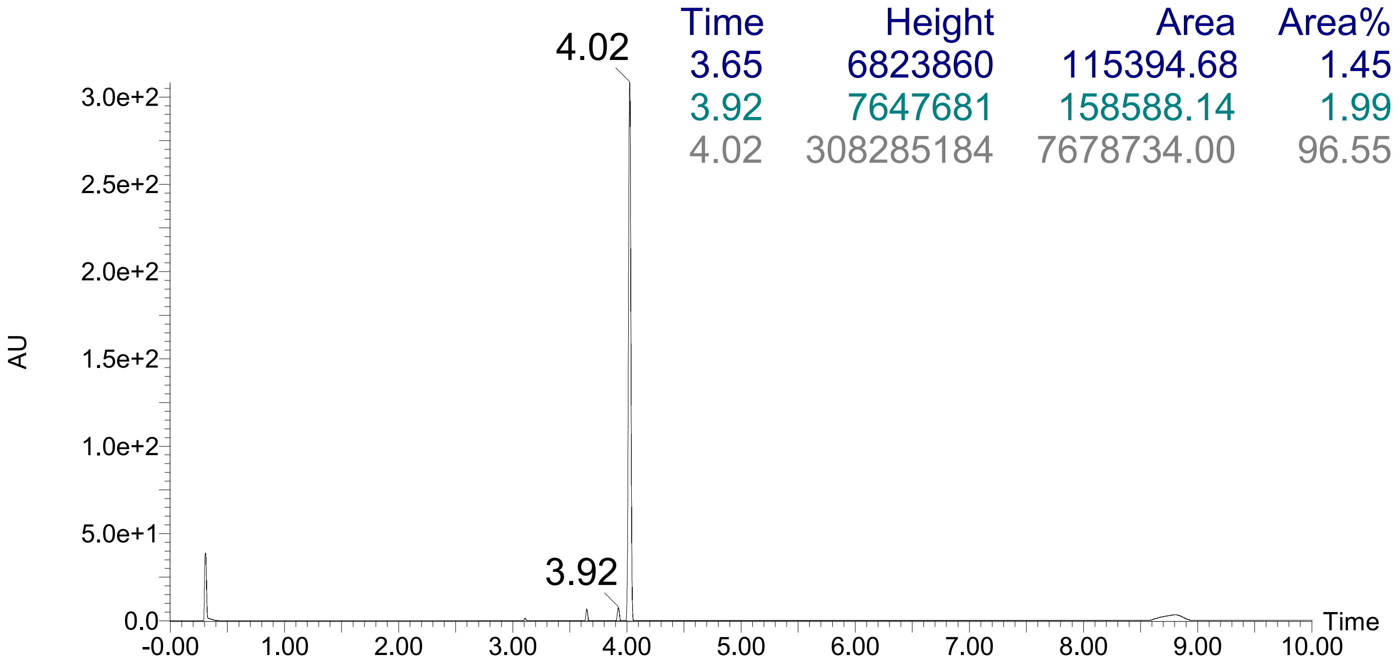
Time Height Area Area%
3.65 6823860 115394.68 1.45
3.92 7647681 158588.14 1.99
4.02 308285184 7678734.00 96.55
4.02
3.92
AU
3.0e+2
2.5e+2
2.0e+2
1.5e+2
1.0e+2
5.0e+1
0.0
Time
-0.00
1.00
2.00
3.00
4.00
5.00
6.00
7.00
8.00
9.00
10.00

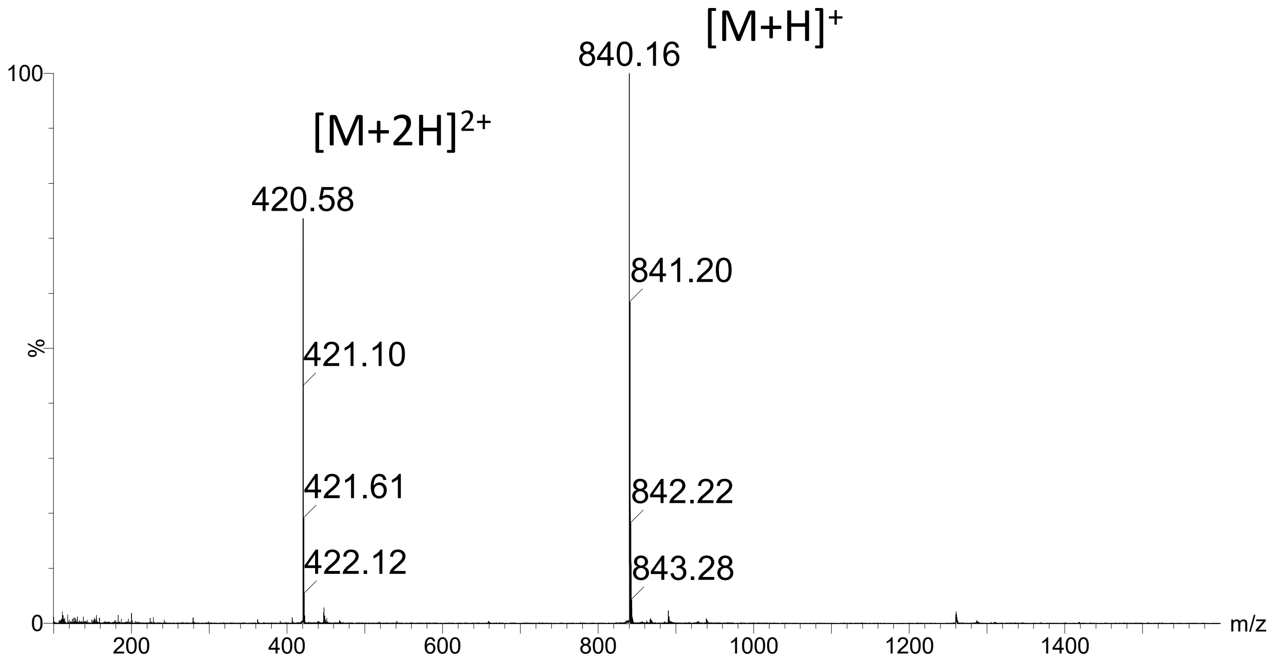
[M+H]+
840.16
[M+2H]2+
420.58
841.20
421.10
421.61
842.22
422.12
843.28
100
%
0
m/z
200
400
600
800
1000
1200
1400

JYQ-195

Time	Height	Area	Area%
3.66	17566310	191432.28	2.53
3.83	4728744	88709.19	1.17
4.03	298113696	7272005.50	96.29

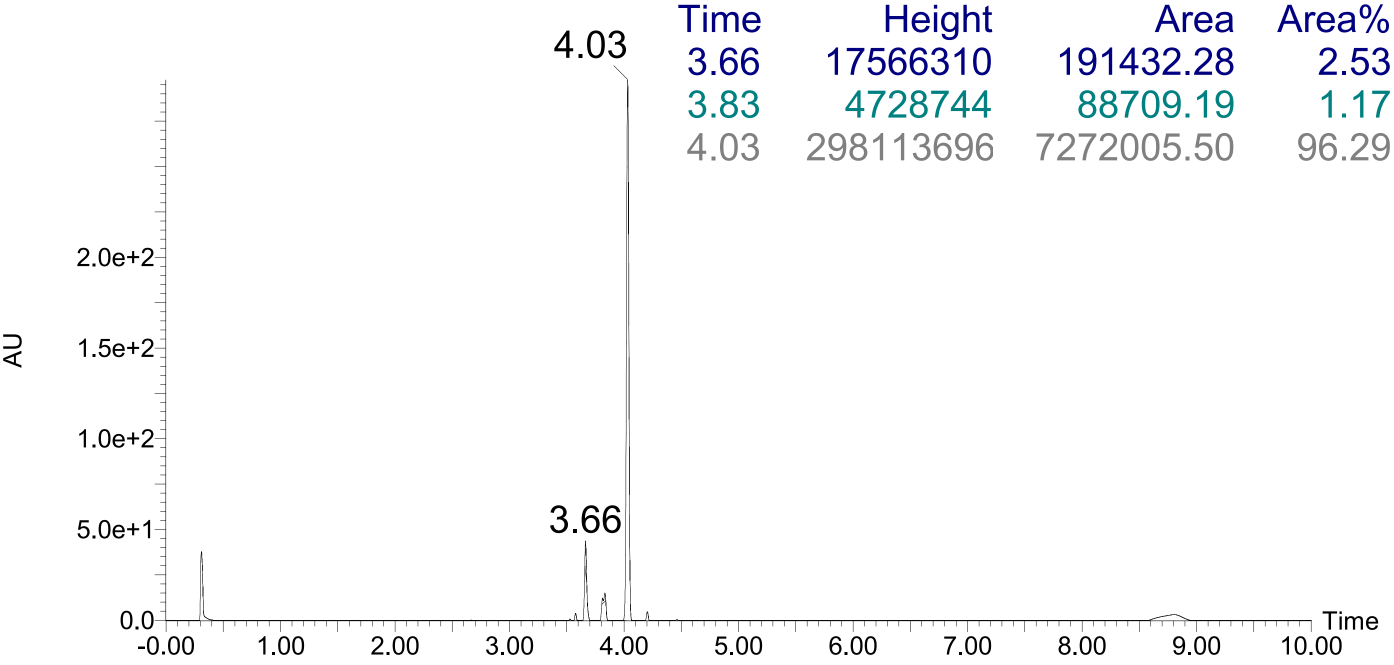

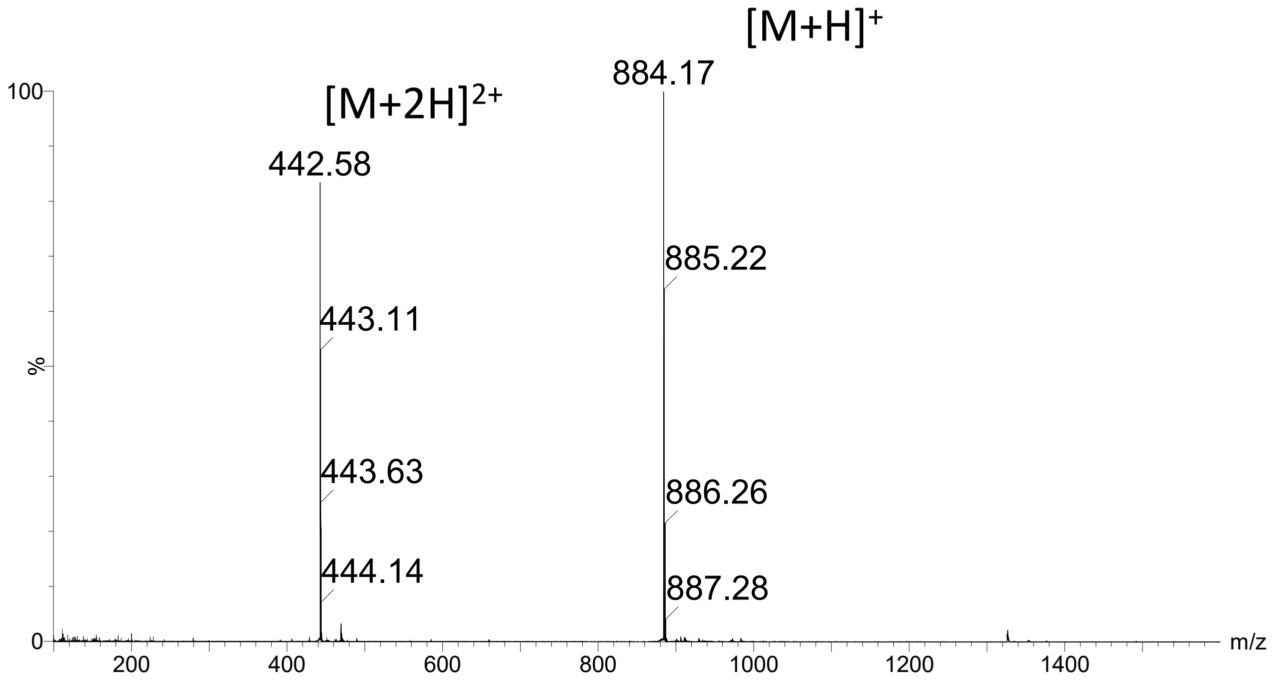

Supplement: Supplementary file 1 — jm3c02410_si_001.pdf [file jm3c02410_si_001.pdf]
